# Direct Regioselective C-H Cyanation of Purines

**DOI:** 10.3390/molecules28030914

**Published:** 2023-01-17

**Authors:** Luyong Li, Jie Hu, Yuqing Fu, Xiaolin Shi, Hongguang Du, Jiaxi Xu, Ning Chen

**Affiliations:** Department of Organic Chemistry, College of Chemistry, Beijing University of Chemical Technology, #15 Beisanhuan East Road, Chaoyang District, Beijing 100029, China

**Keywords:** purine, cyanation, nitrile, regioselective, transition metal-free

## Abstract

A direct regioselective C-H cyanation of purines was developed through a sequential triflic anhydride activation, nucleophilic cyanation with TMSCN, followed by a process of base-mediated elimination of triflous acid (CF_3_SO_2_H). In most cases, the direct C-H cyanation occurred on the electron-rich imidazole motif of purines, affording 8-cyanated purine derivatives in moderate to excellent yields. Various functional groups, including allyl, alkynyl, ketone, ester, nitro et al. were tolerated and acted as a C8 directing group. The electron-donating 6-diethylamino, as C2-directing group substituent, can switch the regioselectivity of purine from 8- to 2-position, enabling the synthesis of 8- and 2-cyano 6-dialkylaminopurines from corresponding 6-chloropurine in different reaction order. Further functional manipulations of the cyano group allow the conversions of 8-cyanopurines to corresponding purine amides, imidates, imidothioates, imidamides, oxazolines, and isothiazoles.

## 1. Introduction

**Scheme 1:**
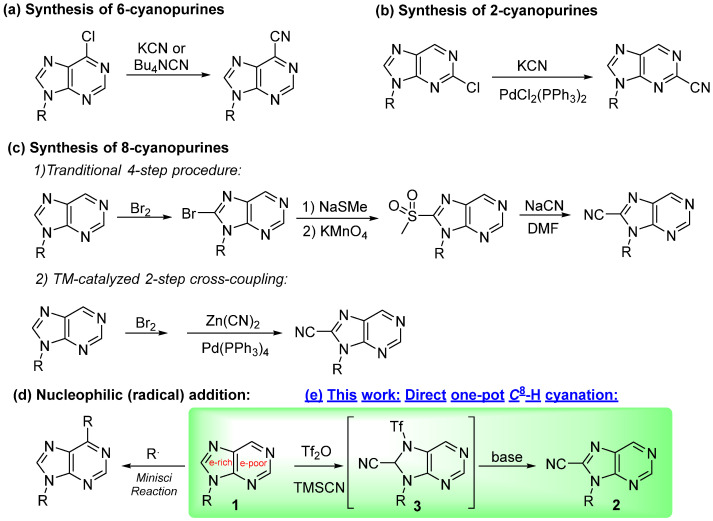
Cyanation of purines.

Purine is a fundamental motif in DNA and RNA nucleic acids, and a primary heterocyclic framework in pharmaceuticals and medicinal chemistry. Purine derivatives bearing a cyano group on their framework have received a great deal of attention due to their biological activities such as antimalarial activity [1]. In addition, they also serve as T. brucei’s cysteine protease inhibitors to cure the Human African trypanosomiasis [2]. The cyanation of purines is generally derived from the corresponding purine halides, with 6-chloropurines as the most useful one, via either an S_N_2Ar process with KCN [3]/Bu_4_NCN [4] (Scheme 1a) or palladium catalyzed cross-coupling with Zn(CN)_2_ [5,6,7]. The cyanation of less reactive 2-chloropurines required harsh reaction conditions [2,8,9] or a transition-metal (TM) catalysis (Scheme 1b) [10,11]. Similarly, 8-cyanopurines were prepared from the corresponding 8-halopurines through a 3/4-step traditional protocol involving swapping the bromine to a more electronegative fluorine [12] or sulfonyl group [13,14], or a transition metal (TM)-catalyzed cross coupling (Scheme 1c) [15,16]. Generally, the cyanation of purines required multiple-step synthesis, with extremely toxic agent (KCN or Bu_4_CN) or the TM catalysis. Owing to the fact that 6- and 2-chloropurines are commercially available and easily obtained from adenine and guanine, we thus planned to develop an expedient and highly regioselective *C*^8^-H cyanation protocol through the triflic anhydride activation on the electron-rich imidazole motif of purines.

As is generally known, purine consists of an electron-deficient pyrimidine and an electron-rich imidazole partner (Scheme 1d). In other previous work and our own work, when purines were exposed to nucleophilic reagents such as Grignard reagents [17,18,19,20] and nucleophilic radical agents (Minisci reaction) [21,22,23,24,25,26], the regioselectivity of the reaction predominantly lay at the electron-deficient 6-position [12,13,14,15,16]. In contrast, when the electrophilic bromine was introduced, a *C*^8^-brominated purine derivative was obtained (Scheme 1c). To facilitate cyanide-type nucleophilic attack at the 8-position, the polarity of electron-rich imidazole motif should be reversed. In our previous work, we found that trifluoroacetic acid was critical to activate the pyrimidine core of the purine via protonation, facilitating the C^6^-arylation of purines with arylboronic acid through a Minisci-type reaction [24]. The research also revealed that with the equivalents of trifluoroacetic acid increased, a small amount of 2,8-biarylated purines was found in the transformation [24]. We thus envisaged that with a suitable activator, the polarity of the purine core would be reversed, that is the electrophilic site might switch from the originally electron-deficient pyrimidine to the electron-rich imidazole motif. After a series of investigations on Lewis acids, triflic anhydride (Tf_2_O) was found to be suitable for this polarity inversion. This interesting development was succeeded in the application of the *C*^8^ or *C*^2^-cyanation with trimethylsilyl cyanide (TMSCN) through a one-pot procedure (Scheme 1e).

## 2. Results and Discussion

In our model reaction, 9-benzylpurine (**1a**), holding three reactive sites at 2-, 6-, and 8-positions of the purine skeletal, was explored first in the presence of TFA (trifluoroacetic acid), a reagent used in our previous work [24], but no reaction occurred. A strong acid TfOH and its alternative TMSOTf cannot promote the reaction either (Scheme 1, entry 2). A typical Lewis acid BF_3_^.^OEt_2_ was investigated but the reaction was intact (entry 3). Tf_2_O is a useful electrophile to activate 6-membered pyridine compounds, as reported by Corey’s [27], McNally’s [28], and Dixon’s groups [29]. We were pleased to discover that the reaction proceeded smoothly to afford the desired 8-cyanoproduct **2a**, whose structure was verified by both ^1^H NMR with the signal loss of 8-H at 8.0 ppm and single crystal XRD (CCDC: 2217776; Table 1). Further solvent evaluation showed that the reaction did not occur in THF or MeCN but reacted smoothly in toluene and haloalkane solvents (entries 5 and 6). Various bases, including pyridine (Py), Et_3_N, *N*-methylmorpholine (NMM), 1,5-diazabicyclo [4.3.0]non-5-ene (DBN), 1,8-diazabicyclo [5.4.0]undec-7-ene (DBU), and 1,4-diazabicyclo [2.2.2]octane (DABCO), were investigated, but DBU provided the best result (Table 1, entries 11~15). The reaction time, temperature, and the equivalents of reactants were finally screened, with the detailed information listed in Supplementary Materials (SI, page 3).

**Scheme 2:**
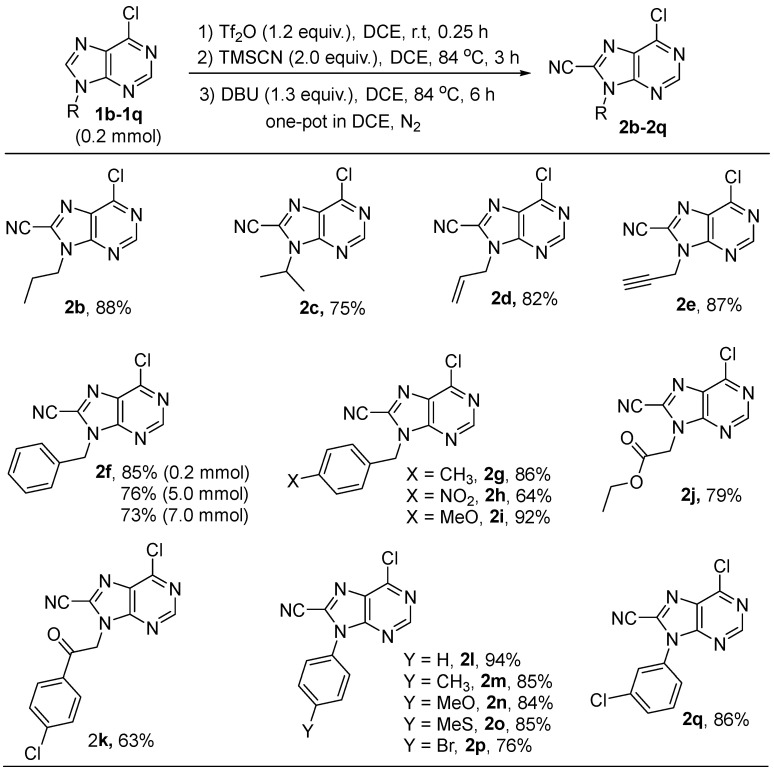
Substrates scope with 6-chloropurines.

Armed with the optimized conditions, we embarked on the task of investigating on the substrate scope. 6-Chloropurines are the most widely used substrates for the construction of various other substituted purine derivatives. With respect to this, various 9-alkyl 6-chloropurines (**1**) were investigated (Scheme 2). 9-Propyl and more sterically hindered isopropyl purines (**1b** and **1c**) provided the desired 8-cyano products **2b** and **2c** in 88% and 75% of yields, respectively. Substrates bearing allyl (**1d**) and propynal (**1e**), were tolerated under current conditions. 9-Benzyl (**1f**) and its derivatives with electron-donating methyl (**1g**), methoxy (**1i**), and electron-withdrawing nitro (**1h**) all caused the corresponding 8-cyano products in satisfactory to excellent yields, but electron-rich substrates obviously displayed better reactivity than the electron-deficient one (**2g**, **2h** vs. **2i**). Moreover, the electrophilic ketone and ester substituents (**1j** and **1k**) were also tolerated under current conditions, delivering the desired **2j** and **2k** in 79% and 63% of yields, respectively. *N*^9^-Arylpurines are highly suitable in this transformation. 6-Chloro-*N*-phenylpurine provided cyanated product **2l** in 94% of the yield, while substituted purines, including *N-para*-methyl **1m**, methoxy **1n**, methylthio **1o**, bromo **1p,** and *N*-*meta*-chloro **1q** all produced the desired products **2m**~**2q** in 76~94% of yields.

**Scheme 3:**
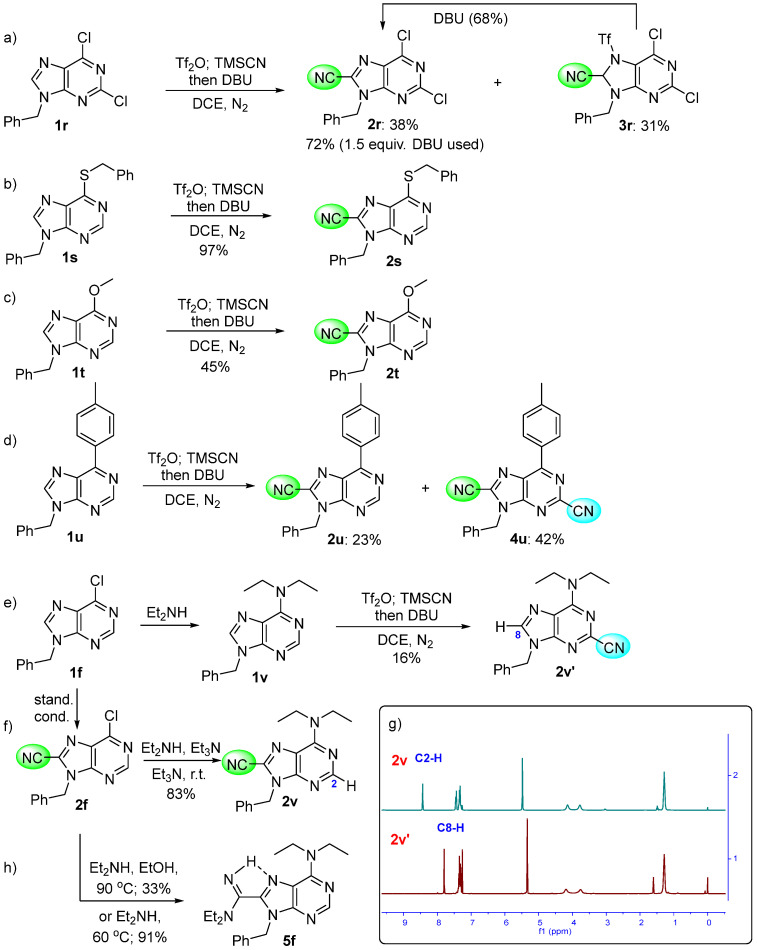
Substrates scope with other purines. The reactions in Scheme (**a**–**h**) were carried out in 0.2 mmol scale under standard condition. Scheme (**g**): the ^1^H NMR comparation of **2v** and **2v’**.

**Figure 1:**
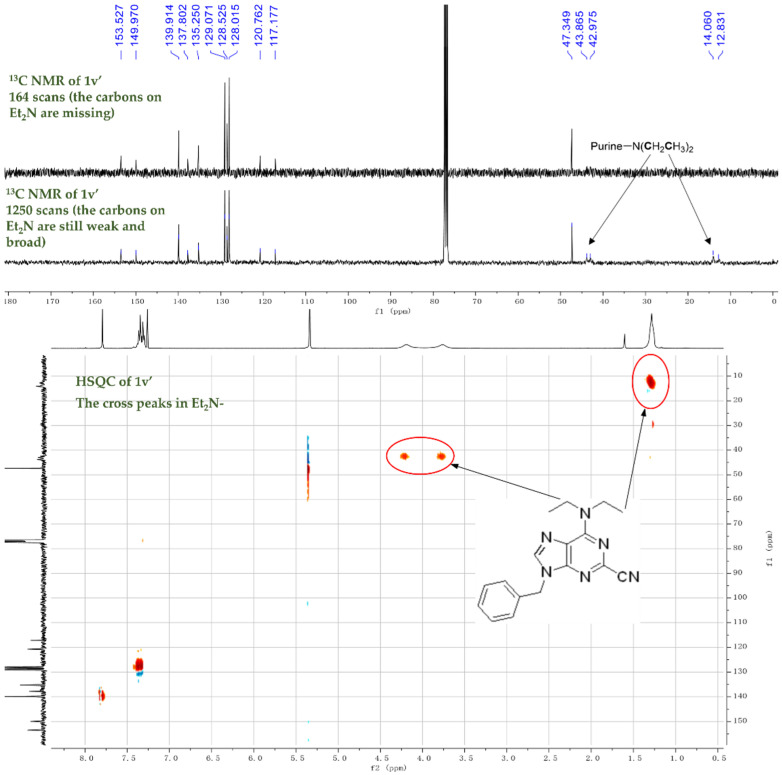
The ^13^C NMR (101 MHz; 164 scans and 1250 scans) and the HSQC of **2v’** (the structure identification of the broad and weak carbons in Et_2_N). The sample was prepared from 10 mg of **2v** in 0.5 mL CDCl_3_.

We also briefly investigated the purines bearing other substituents besides the chlorine (Scheme 3). It is worth noting that the reactive intermediates **3** were unstable, sensitive, and generally difficult to isolate. Nevertheless, the key intermediate **3r** was isolated when 2,6-dichloropurine (**1r**) was exploited. Moreover, **3r** was transformed into the desired **2r** in 68% of the yield by treatment with 1 equiv. of DBU (Scheme 3a). The yield of **2r** was increased to 72% when 1.5 equiv. of DBU was added. Moreover, 6-benzylthio purine **1s** afforded the desired 8-cyanated product **2s** in 97% of the yield, whereas 6-methoxy purine **1t** caused the corresponding **2t** in only 45% of the yield, with most of the substrate remaining unreactive (Scheme 3b,c). Another interesting result was obtained from the reaction of 6-*p-*tolyl purine **1u**, in which a mixture of 8-monocyanated product **2u** (23%) and 2,8-dicyanoated **4u** (42%) were generated (Scheme 3d). The results demonstrated that the electron-donating alkoxy and aryl substituents might enhance the electron density of the pyrimidine motif of the purines, causing the decreasing regioselectivity over the imidazole motif. To verify our hypothesis, we introduced a strong electron-donating diethylamino (Et_2_N) on the 6-position of purine (**1v**) but the reaction of **1v** was relatively complicated. After careful isolation and structural identification, a purine derivative **2v’** bearing the nitrile on its 2-position, rather than common 8-position, was obtained albeit in only 16% of the yield (Scheme 3e). None of the desired 8-cyanopurine **2v** was traced by the TLC and ^1^H NMR of the reaction system. Obviously, the strong donating diethylamino completely switched the reactivity of the purine skeletal, enabling the pyrimidine motif to be more reactive than the imidazole counterpart. To obtain the desired 8-cyano-6-diethylaminopurine **2v**, the direct amination reaction of 6-chloro-8-cyanopurine **2f** and diethylamine (Scheme 3f) was performed in the presence of Et_3_N in EtOH, providing the desired **2v** in 83% of the yield. The ^1^H NMR of 8-cyanopurine **2v** and 2-cyanopurine **2v’** are totally different (Scheme 3g), as well as the ^13^C NMR shown in SI (page S25–S28). It is worth mentioning that the alkyl carbons in Et_2_N in both **2v** and **2v’** are hard to emerge especially the **2v’** in which 1250 scans (~80 min) were tested while the peak appearances were broad and weak (much weaker than the *ipso* aromatic carbons). The HSQC test of **2v’** was thus performed for further verification of the alkyl carbons in Et_2_N (Figure 1). Interestingly, when heating the reaction system, an imidaminopurine derivative **5f** was obtained in 33% of the yield from a Et_2_NH/EtOH solution at 90 °C through a base-mediated Pinner-type reaction [30]. This yield was further optimized to 91% with pure Et_2_NH as solution at 60 °C (Scheme 3h). The results demonstrated that besides the 6-chloro reactive site of purine, the nitrile group is also a highly potential electrophilic reactive position.

**Scheme 4:**
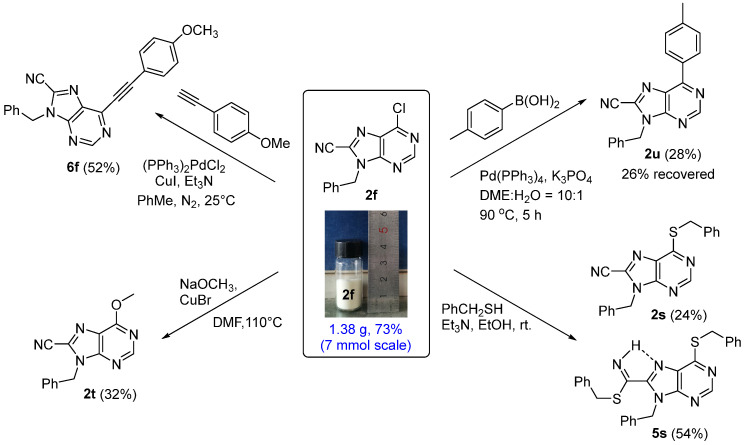
Gram-scale synthesis of **2f** and its derivation.

As building blocks in purine chemistry, 6-chloropurines can be converted into various purine derivatives (Scheme 4). Gram-scale reactions with **1f** were carried out, affording **2f** in 1.03 g (76%, 5 mmol scale) and 1.38 g (73%, 7 mmol scale) (Scheme 2-**2f**). A Sonogashira coupling product **6f** was obtained in 52% of the yield from the reaction of **2f** with *p*-methoxyphenylacetylene under the palladium catalysis [31]. Similarly, a Suzuki–Miyaura coupling with *p-*tolylboronic acid delivered **2u** in 28% of the yield, along with **2f** recovered in 26% of the yield [32]. The reaction of **1f** with sodium methoxide provided a complicated mixture, but it reacted smoothly to cause the desired 6-methoxypurine **2t** in the presence of CuBr (10 mol%) [33]. Moving forward, the alkylthiolation with benzyl thiol produced the desired 6-benzylthio purine **2s** in 24% of the yield and an extra thiol to nitrile adduct, imidothioate **5s**, similar to the adduct from Et_2_NH (Scheme 4) in 54% of the yield. The results are similar to our previous discovery, in which an interesting adduct was formed from the addition of potassium thioacetate (KSAc) to acetonitrile (MeCN) [34].

**Scheme 5:**
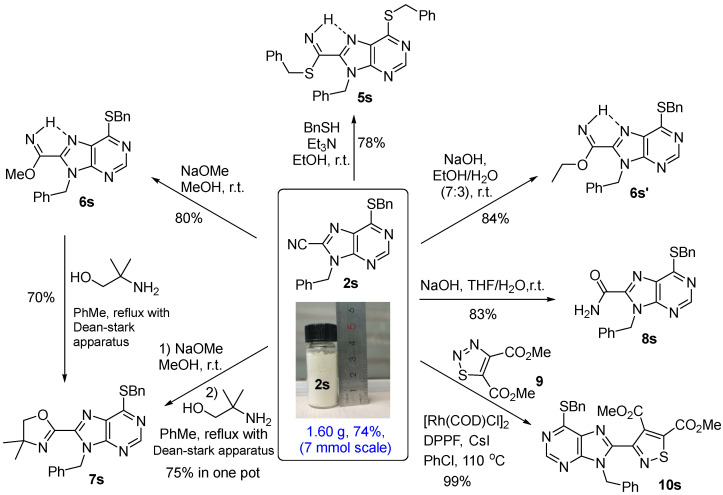
Gram-scale synthesis of **2s** and its synthetical application.

The further derivation of the nitrile group was performed with 9-benzyl-6-benzylthio-8-cyano purine (**1s**) as an example. A 6 mmol Gram-scaled reaction was carried out, from which 1.60 g of desired **2s** was obtained in 74% of the yield (Scheme 5). Treatment of **1s** with benzylthiol (BnSH) at r.t. afforded the desired adduct **5s** in 78% of the yield (Scheme 5). The result indicated that the cyano group on the purine skeleton was more reactive than the common arylnitriles. We thus explored its reaction with sodium methoxide (NaOMe), which produced the corresponding adduct methyl carbimidate **6s** in 80% of the yield. Generally, carbimidate was commonly unstable but herein **6s** along with **5s** were generated and could be separated through the column chromatography, probably due to the stabilization effect by the intramolecular hydrogen bonding with the nitrogen in the purine scaffold. As that carbimidate was also a general intermediate in the synthesis of oxazoline [35], we thus reacted **6s** with 2-amino-2-methylpropan-1-ol in toluene. After an overnight Dean–Stark reflux (~10 h), oxazoline **7s** was obtained in 70% of the yield. The transformation of **2s** to **7s** was also carried out through a one-pot process, delivering a total yield of 75%. The hydrolysis of a nitrile to an amide is a classic transformation. However, the direct hydrolysis of **2s** in aqueous NaOH solution failed due to its poor solubility in water, with all starting materials floating on the water surface. The addition of EtOH can disperse **2s** into the solution, but only ethyl carbimidate homolog **6s’** was obtained with NaOH in EtOH/H_2_O (7:3). Finally, the desired amide **8s** was attained in a solution of NaOH in THF/H_2_O. It is worth noting that the isolation of **5s**, **6s**, **6s’**, and **8s** were quite convenient because all adducts directly precipitated from their solutions and only filtration and drying were required in the work-up process. Very recently, Yang and coworkers reported a highly effective diastereoselective (3 + 2) transannulations of 1,2,3-thiadiazoles with cyanoepoxides to afford the corresponding isothiazoles under the catalysis of [Rh(COD)Cl]_2_ and DPPF [36]. We also developed the transannulation of 1,2,3-thiazoles with α,β-unsaturated nitriles [37]. Finally, referring to Yang’s conditions, the reaction of **2s** with thiadiazole **9** caused the desired isothiazole **10s** in a quantitative yield.

**Scheme 6:**
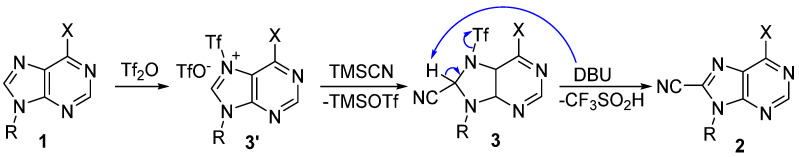
Reaction mechanism of the 8-cyanation of purines.

The proposed reaction mechanism is portrayed in Scheme 6. Triflylation of the most nucleophilic nitrogen (N-7) of the purines **1** activates the electron-rich imidazole motif and enables it to be subjected to the subsequent nucleophilic attack by TMSCN, in this way the sensitive and unstable key adducts **3** are formed. We believe that the base-mediated elimination of triflous acid (CF_3_SO_2_H) from **3** furnishes the formal C-H cyanation products **2**, and the transformation of intermediate **3r** in Scheme 3 solidly supports this suggested mechanistic insight.

## 3. Materials and Methods

### 3.1. General Information

Moisture sensitive reactions were carried out under the nitrogen atmosphere with flame-dried apparatus. The solvents, THF, PhCl, PhMe, CHCl_3_, used in Table 1 and the DCE used under the standard conditions, were freshly distilled by referring the standard dehydration process. Other solvents used in Scheme 4, Scheme 5 and Scheme 6, besides DCE, were used directly from the commercial source. Melting points were obtained on a MP-500 melting point apparatus and are uncorrected. TLC were performed on silica gel GF254 plates, the plates of which were visualized under UV light. ^1^H and ^13^C-NMR spectra were recorded on a 400 MHz Bruker spectrometer in CDCl_3_ with TMS as an internal standard and the chemical shifts (δ) are reported in parts per million (ppm). HRMS measurements were carried out on an Agilent LC/MSD ESI-TOF mass spectrometer. Petroleum ether (PE, 60–90 °C) and ethyl acetate (EtOAc) were used for column chromatography.

All purine substrates were synthesized according to our previous work [24,38].

### 3.2. General Procedure for the 8-Cyanation of N-Benzylpurine, 6-Chloropurines and 6-Alkoxy, Alkylthio, and Aryl Purines 2

To a frame-dried 10 mL vial was added solid purine (0.2 mmol), and the vial was capped with a septum followed by the N_2_ protection. Anhydrous 1,2-dichloroethane (2.0 mL) was then injected and the reaction was cooled in an ice-water bath. Triflic anhydride (0.24 mmol, 40 μL) was then injected dropwise, and the reaction system was stirred at 0 °C for 15 min followed by the injection of trimethylsilyl cyanide (TMSCN; 0.4 mmol, 50 μL). The system was stirred at 84 °C for 3 h. After addition of 1,8-diazabicyclo [5.4.0]undec-7-ene (DBU, 39 μL, 1.3 mmol) the mixture was stirred at 84 °C for another 6 h. After cooling to r.t., the reaction was quenched with 1.0 mL of saturated NaHCO_3_ solution and the organic phase was separated. The aqueous phase was then extracted with 3.0 mL of DCE. The combined organic phase was dried over anhydrous Na_2_SO_4_. After removing the solvent in vacuo, the residue was purified by silica gel column chromatography with a mixture PE and EtOAc as the eluent.

The Gram-scale reactions of **2f** and **2s** were scaled up proportionally.

#### 3.2.1. 9-Benzyl-9H-purine-8-carbonitrile (**2a**)

Colorless crystals, mp: 178–180 °C. [CCDC: 2217776] yield: 26 mg, 54%. R*_f_* = 0.12 (PE/EtOAc = 5/1, *v*/*v*). ^1^H NMR (400 MHz, CDCl_3_) *δ* 9.26 (s, 1H, C6-H in purine), 9.17 (s, 2H, C2-H in purine), 7.48–7.45 (m, 2H, ArH), 7.37–7.32 (m, 3H, ArH), and 5.61 (s, 2H, NCH_2_). ^13^C NMR (101 MHz, CDCl_3_) *δ* 155.4, 151.3, 150.2, 133.8, 133.0, 129.2, 129.1, 128.7, 128.4, 110.2, and 47.8. HRMS (ESI): *m*/*z* [M + H]^+^ calcd. for C_13_H_10_N_5_^+^, 236.0931; found: 236.0923.

#### 3.2.2. 6-Chloro-9-propyl-9H-purine-8-carbonitrile (**2b**)

Colorless crystals, mp: 81–83 °C, yield: 39 mg, 88%. R*_f_* = 0.75 (PE/EtOAc = 1/1, *v*/*v*). ^1^H NMR (400 MHz, CDCl_3_) *δ* 8.89 (s, 1H, C2-H in purine), 4.46 (t, *J* = 7.2 Hz, 2H, NCH_2_), 2.03 (sext, *J* = 7.2 Hz, 2H, CH_2_), and 1.00 (t, *J* = 7.2 Hz, 3H, CH_3_). ^13^C NMR (101 MHz, CDCl_3_) *δ* 154.4, 153.9, 150.9, 131.1, 129.1, 109.7, 46.9, 23.2, and 11.0. HRMS (ESI): *m*/*z* [M + H]^+^ calcd. for C_9_H_9_ClN_5_^+^, 222.0541; found: 222.0548.

#### 3.2.3. 6-Chloro-9-isopropyl-9H-purine-8-carbonitrile (**2c**)

Colorless crystals, mp: 112–113 °C, yield: 33 mg, 75%, R*_f_* = 0.65 (PE/EtOAc = 3/1, *v*/*v*). ^1^H NMR (400 MHz, CDCl_3_) δ 8.86 (s, 1H, C2-H in purine), 5.15 (sept, *J* = 6.8 Hz, 1H, NCH_2_), and 1.80 (d, *J* = 6.8 Hz, 6H, 2CH_3_). ^13^C NMR (101 MHz, CDCl_3_) δ 153.9, 153.8, 150.6, 131.4, 127.9, 110.2, 51.3, and 21.6. HRMS (ESI): *m/z* [M + H]^+^ calcd. for C_9_H_9_ClN_5_^+^, 222.0541; found: 222.0537.

#### 3.2.4. 9-Allyl-6-chloro-9h-purine-8-carbonitrile (**2d**)

Colorless crystals, mp: 108–110 °C, yield: 36 mg, 82%. R*_f_* = 0.50 (PE/EtOAc = 3/1, *v*/*v*). ^1^H NMR (400 MHz, CDCl_3_) *δ* 8.90 (s, 1H, C2-H in purine), 6.03 (ddt, *J* = 17.2, 10.4, 6.0, 17.2 Hz, 1H, CH), 5.42 (d, *J* = 10.0 Hz, 1H, 1H in CH_2_), 5.32 (d, *J* = 17.2 Hz, 1H, 1H in CH_2_), and 5.09 (d, *J* = 6.0 Hz, 2H, NCH_2_). ^13^C NMR (101 MHz, CDCl_3_) *δ* 154.6, 153.9, 150.7, 131.0, 129.4, 128.8, 121.2, 109.5, and 46.9. HRMS (ESI): *m*/*z* [M + H]^+^ calcd. for C_9_H_7_ClN_5_^+^, 220.0384; found: 222.0368.

#### 3.2.5. 6-Chloro-9-(propargyl)-9H-purine-8-carbonitrile (**2e**)

Colorless crystals, mp: 134–136 °C, yield: 38 mg, 87%, R*_f_* = 0.77 (PE/EtOAc = 1/1, *v*/*v*). ^1^H NMR (400 MHz, CDCl_3_) *δ* 8.93 (s, 1H, C2-H in purine), 5.25 (d, *J* = 2.4 Hz, 2H, NCH_2_), and 2.57 (t, *J* = 2.4 Hz, 1H, CH). ^13^C NMR (101 MHz, CDCl_3_) *δ* 154.9, 154.1, 150.3, 130.8, 128.3, 109.1, 76.1, 74.0, and 33.9. HRMS (ESI): *m*/*z* [M + H]^+^ calcd. for C_9_H_5_ClN_5_^+^, 218.0228; found: 218.0235.

#### 3.2.6. 9-Benzyl-6-chloro-9H-purine-8-carbonitrile (**2f**)

Colorless crystals, mp: 102–104 °C, yield: 46 mg, 85%. R*_f_* = 0.51 (PE/EtOAc = 3/1, *v*/*v*). ^1^H NMR (400 MHz, CDCl_3_) *δ* 8.94 (s, 1H, C2-H in purine), 7.49–7.46 (m, 2H, ArH), 7.39–7.36 (m, 3H, ArH), and 5.62 (s, 2H, NCH_2_). ^13^C NMR (101 MHz, CDCl_3_) *δ* 154.7, 153.9, 150.8, 133.4, 131.0, 129.35, 129.33, 128.6, 128.5, 109.9, and 48.6. HRMS (ESI): *m*/*z* [M + H]^+^ calcd. for C_13_H_9_ClN_5_^+^, 270.0541; found: 270.0550.

#### 3.2.7. 6-Chloro-9-(4-methylbenzyl)-9h-purine-8-carbonitrile (**2g**)

Colorless crystals, mp: 91–92 °C. Yield: 49 mg, 86%. R*_f_* = 0.62 (PE/EtOAc = 3/1, *v*/*v*). ^1^H NMR (400 MHz, CDCl_3_) *δ* 8.93 (s, 1H, C2-H in purine), 7.36 (d, *J* = 8.0 Hz, 2H, ArH), 7.16 (d, *J* = 8.0 Hz, 2H, ArH), 5.58 (s, 2H, NCH_2_), and 2.32 (s, 3H, CH_3_). ^13^C NMR (101 MHz, CDCl_3_) *δ* 154.6, 153.8, 150.7, 139.4, 131.0, 130.5, 129.9, 128.6, 128.5, 109.9, 48.4, and 21.1. HRMS (ESI): *m*/*z* [M + H]^+^ calcd. for C_14_H_11_ClN_5_^+^, 284.0697; found: 284.0707.

#### 3.2.8. 6-Chloro-9-(4-nitrobenzyl)-9H-purine-8-carbonitrile (**2h**)

Light yellow crystals, mp: 217–220 °C. Yield: 40 mg, 64%. R*_f_* = 0.60 (PE/EtOAc = 1/1, *v*/*v*). ^1^H NMR (400 MHz, DMSO-*d*_6_) *δ* 9.04 (s, 1H, C2-H in purine), 8.22 (d, *J* = 8.8 Hz, 2H, ArH), 7.65 (d, *J* = 8.8 Hz, 2H, ArH), and 5.85 (s, 2H, NCH_2_). ^13^C NMR (101 MHz, DMSO-*d*_6_) *δ* 154.7, 152.0, 151.1, 147.4, 141.7, 130.4 129.4, 129.0, 123.9, 110.2, and 47.1. HRMS (ESI): *m*/*z* [M + H]^+^ calcd. for C_13_H_8_ClN_5_^+^, 315.0392; found: 315.0401.

#### 3.2.9. 6-Chloro-9-(4-methoxybenzyl)-9H-purine-8-carbonitrile (**2i**)

Colorless crystals, mp: 158–160 °C. Yield: 55 mg, 92%, R*_f_* = 0.81 (PE/EtOAc = 1/1, *v*/*v*). ^1^H NMR (400 MHz, CDCl_3_) *δ* 8.94 (s, 1H, C2-H in purine), 7.44 (d, *J* = 8.8 Hz, 2H, ArH), 6.88 (d, *J* = 8.8 Hz, 2H, ArH), 5.56 (s, 2H, NCH_2_), and 3.78 (s, 3H, OCH_3_). ^13^C NMR (101 MHz, CDCl_3_) *δ* 160.3, 154.6, 153.9, 150.7, 131.1, 130.2, 128.6, 125.6, 114.6, 110.0, 55.3, and 48.2. HRMS (ESI): *m*/*z* [M + H]^+^ calcd. for C_14_H_11_ClN_5_O^+^, 300.0647; found: 300.0640.

#### 3.2.10. Ethyl-2-(6-chloro-8-cyano-9H-purin-9-yl)acetate (**2j**)

Colorless crystals, mp: 108–109 °C. Yield: 42 mg, 79%. R*_f_* = 0.40 (PE/EtOAc = 3/1, *v*/*v*). ^1^H NMR (400 MHz, CDCl_3_) *δ* 8.88 (s, 1H, C2-H in purine), 5.20 (s, 2H, NCH_2_), 4.31 (q, *J* = 7.2 Hz, 2H, OCH_2_), and 1.33 (t, *J* = 7.2 Hz, 3H, CH_3_). ^13^C NMR (101 MHz, CDCl_3_) *δ* 165.0, 154.8, 154.0, 150.9, 130.8, 129.3, 109.2, 63.3, 44.7, and 14.0. HRMS (ESI): *m*/*z* [M + H]^+^ calcd. for C_10_H_9_ClN_5_O_2_^+^, 266.0439; found: 266.0432.

#### 3.2.11. 6-Chloro-9-(2-(4-chlorophenyl)-2-oxoethyl)-9H-purine-8-carbonitrile (**2k**)

Colorless crystals, mp: 204–207 °C. Yield: 42 mg, 63%. R*_f_* = 0.65 (PE/EtOAc = 1/1, *v*/*v*). ^1^H NMR (400 MHz, CDCl_3_) *δ* 8.85 (s, 1H, C2-H in purine), 8.01 (d, *J* = 8.8 Hz, 2H, ArH), 7.59 (d, *J* = 8.8 Hz, 2H, ArH), and 5.87 (s, 2H, NCH_2_). ^13^C NMR (101 MHz, CDCl_3_) *δ* 187.5, 154.7, 154.1, 151.1, 142.0, 131.5, 131.0, 129.8, 129.7, 109.4, and 49.6. HRMS (ESI): *m*/*z* [M + H]^+^ calcd. for C_14_H_8_Cl_2_N_5_O^+^, 332.0100; found: 332.0097.

#### 3.2.12. 6-Chloro-9-phenyl-9H-purine-8-carbonitrile (**2l**)

Colorless crystals, mp: 158–161 °C. Yield: 48 mg, 94%. R*_f_* = 0.85 (PE/EtOAc = 1/1, *v*/*v*). ^1^H NMR (400 MHz, CDCl_3_) *δ* 8.91 (s, 1H, C2-H in purine), 7.65–7.70 (m, 3H, ArH), and 7.59–7.62 (m, 2H, ArH). ^13^C NMR (101 MHz, CDCl_3_) *δ* 155.2, 154.2, 151.1, 131.4, 131.1, 130.9, 130.3, 128.7, 125.9, and 109.7. HRMS (ESI): *m*/*z* [M+H]^+^ calcd. for C_12_H_7_ClN_5_^+^, 256.0384; found: 256.0371.

#### 3.2.13. 6-Chloro-9-(p-tolyl)-9H-purine-8-carbonitrile (**2m**)

Colorless crystals, mp: 120–123 °C, yield: 46 mg, 85%. R*_f_* = 0.80 (PE/EtOAc = 1/1, *v*/*v*). ^1^H NMR (400 MHz, CDCl_3_) *δ* 8.90 (s, 1H, C2-H in purine), 7.47 (s, 4H, ArH), and 2.50 (s, 3H, CH_3_). ^13^C NMR (101 MHz, CDCl_3_) *δ* 155.2, 154.2, 151.2, 141.4, 131.1, 130.9, 128.9, 128.8, 125.7, 109.7, and 21.3. HRMS (ESI): *m*/*z* [M + H]^+^ calcd. for C_13_H_9_ClN_5_^+^, 270.0541; found: 270.0544.

#### 3.2.14. 6-Chloro-9-(4-methoxyphenyl)-9H-purine-8-carbonitrile (**2n**)

Colorless crystals, mp: 122–125 °C. Yield: 48 mg, 84%. R*_f_* = 0.75 (PE/EtOAc = 1/1, *v*/*v*). ^1^H NMR (400 MHz, CDCl_3_) *δ* 8.88 (s, 1H, C2-H in purine), 7.49 (d, *J* = 8.8 Hz, 2H, ArH), 7.14 (d, *J* = 8.8 Hz, 2H, ArH), and 3.91 (s, 3H, CH_3_). ^13^C NMR (101 MHz, CDCl_3_) *δ* 161.2, 155.1, 154.1, 151.3, 130.9, 129.0, 127.3, 123.8, 115.4, 109.7, and 55.7. HRMS (ESI): *m*/*z* [M+H]^+^ calcd. for C_13_H_9_ClN_5_O^+^, 286.0490; found: 286.0499.

#### 3.2.15. 6-Chloro-9-(4-(methylthio) phenyl)-9H-purine-8-carbonitrile (**2o**)

Yellow crystals, mp: 149–151 °C. Yield: 51 mg, 85%. R*_f_* = 0.80 (PE/EtOAc = 1/1, *v*/*v*). ^1^H NMR (400 MHz, CDCl_3_) *δ* 8.90 (s, 1H, C2-H in purine), 7.51 (d, *J* = 8.8 Hz, 2H, ArH), 7.47 (d, *J* = 8.8 Hz, 2H, ArH), and 2.57 (s, 3H, CH_3_). ^13^C NMR (101 MHz, CDCl_3_) *δ* 155.2, 154.3, 151.1, 143.3, 131.1, 128.7, 127.7, 127.0, 126.0, 109.7, and 15.2. HRMS (ESI): *m*/*z* [M + H]^+^ calcd. for C_13_H_9_ClN_5_S^+^, 302.0262; found: 302.0281.

#### 3.2.16. 9-(4-bromophenyl)-6-chloro-9H-purine-8-carbonitrile (**2p**)

Colorless crystals, mp: 165–167 °C, yield: 51 mg, 76%. R*_f_* = 0.85 (PE/EtOAc = 1/1, *v*/*v*). ^1^H NMR (400 MHz, CDCl_3_) *δ* 8.92 (s, 1H, C2-H in purine), 7.84–7.81 (m, 2H, ArH), and 7.52–7.26 (m, 2H, ArH). ^13^C NMR (101 MHz, CDCl_3_) *δ* 155.4, 154.5, 150.9, 133.6, 131.1, 130.3, 128.3, 127.3, 125.2, and 109.5. HRMS (ESI): *m*/*z* [M + H]^+^ calcd. for C_12_H_6_BrClN_5_^+^, 333.9490; found: 333.9504.

#### 3.2.17. 6-Chloro-9-(3-chlorophenyl)-9H-purine-8-carbonitrile (**2q**)

Colorless crystals, mp: 139–141 °C. Yield: 48 mg, 83%. R*_f_* = 0.85 (PE/EtOAc = 1/1, *v*/*v*). ^1^H NMR (400 MHz, CDCl_3_) *δ* 8.93 (s, 1H, C2-H in purine), 7.65–7.61 (m, 3H, ArH), and 7.54–7.51 (m, 1H, ArH). ^13^C NMR (101 MHz, CDCl_3_) *δ* 155.4, 154.5, 151.0, 136.1, 132.3, 131.3, 131.2, 131.1, 128.3, 126.3, 124.1, and 109.5. HRMS (ESI): *m*/*z* [M + H]^+^ calcd. for C_12_H_6_Cl_2_N_5_^+^, 289.9995; found: 290.0001.

#### 3.2.18. 9-Benzyl-2,6-dichloro-9h-purine-8-carbonitrile (**2r**) and 9-Benzyl-2,6-dichloro-7-((trifluoromethyl)sulfonyl)-8,9-dihydro-7H-purine-8-carbonitrile (**3r**)

**2r**: Colorless crystals, mp: 141–143 °C. Yield: 116 mg, 38%. R*_f_* = 0.65 (PE/EtOAc = 5/1, *v*/*v*). ^1^H NMR (400 MHz, CDCl_3_) *δ* 7.49–7.38 (m, 5H, ArH), and 5.58 (s, 2H, NCH_2_). ^13^C NMR (101 MHz, CDCl_3_) *δ* 156.3, 154.8, 151.9, 133.0, 130.1, 129.5, 129.4, 129.1, 128.6, 109.6, and 48.8. HRMS (ESI): *m*/*z* [M+H]^+^ calcd. for C_13_H_8_Cl_2_N_5_^+^, [M + H]^+^ 304.0152, found: 304.0155.

**3r**: Colorless oil, yield: 93 mg, 31%. R*_f_* = 0.72 (PE/EtOAc = 5/1, *v*/*v*). ^1^H NMR (400 MHz, CDCl_3_) *δ* 7.50–7.33 (m, 5H, ArH), 6.12 (s, 1H, CH), 5.32 (d, *J* = 15.2 Hz, 1H, NCH), and 4.29 (d, *J* = 15.2 Hz, 1H, NCH). ^13^C NMR (101 MHz, CDCl_3_) *δ* 161.2, 158.1, 145.7, 131.3, 129.74, 129.67, 128.47, 118.9 (q, *J* = 319.7 Hz, CF_3_), 117.3, 110.6, 68.4, and 47.1. ^19^F NMR (376 MHz, CDCl_3_) *δ* -74.13. HRMS (ESI) calcd. for C_14_H_9_Cl_2_F_3_N_5_O_2_S^+^ [M + H]^+^ 437.9801, found: 437.9796.

#### 3.2.19. 9-Benzyl-6-(benzylthio)-9H-purine-8-carbonitrile (**2s**)

Colorless crystals, mp: 111–113 °C. Yield: 69 mg, 97%. R*_f_* = 0.90 (PE/EtOAc = 1/1, *v*/*v*). ^1^H NMR (400 MHz, CDCl_3_) *δ* 8.87 (s, 1H, C2-H in purine), 7.45–7.43 (m, 4H, ArH), 7.27–7.35 (m, 6H, ArH), 5.55 (s, 2H, NCH_2_), and 4.66 (s, 2H, SCH_2_). ^13^C NMR (101 MHz, CDCl_3_) *δ* 164.3, 154.4, 148.0, 136.7, 134.0, 130.6, 129.2, 129.1, 129.0, 128.6, 128.4, 127.5, 125.9, 110.4, 48.0, and 33.0. HRMS (ESI) calcd. for C_20_H_16_N_5_S^+^ [M+H]^+^, 358.1121, found: 358.1131.

#### 3.2.20. 9-Benzyl-6-methoxy-9H-purine-8-carbonitrile (**2t**)

Colorless crystals, mp: 138–141 °C. Yield: 24 mg, 45%. R*_f_* = 0.80 (PE/EtOAc = 1/1, *v*/*v*). ^1^H NMR (400 MHz, CDCl_3_) *δ* 8.70 (s, 1H, C2-H in purine), 7.46–7.44 (m, 2H, ArH), 7.38–7.33 (m, 3H, ArH), 5.57 (s, 2H, NCH_2_), and 4.22 (s, 3H, OCH_3_). ^13^C NMR (101 MHz, CDCl_3_) *δ* 162.2, 155.2, 151.2, 134.1, 129.2, 129.0, 128.4, 125.8, 121.8, 110.5, 54.8, and 48.1. HRMS (ESI) calcd. for C_14_H_12_N_5_O^+^ [M+H]^+^, 266.1036, found: 266.1039.

#### 3.2.21. 9-Benzyl-6-(p-tolyl)-9H-purine-8-carbonitrile (**2u**) and 9-Benzyl-6-(p-tolyl)-9H-purine-2,8-dicarbonitrile (**4u**)

**2u**: Colorless crystals, mp: 138–140 °C. Yield: 25 mg, 23%. R*_f_* = 0.19 (PE/EtOAc = 30/1, *v*/*v*). ^1^H NMR (400 MHz, CDCl_3_) *δ* 9.14 (s, 1H, C2-H in purine), 8.70 (d, *J* = 8.0 Hz, 2H, ArH), 7.51–7.48 (m, 2H, ArH), 7.38–7.35 (m, 5H, ArH), 5.63 (s, 2H, NCH_2_), and 2.46 (s, 3H, CH_3_). ^13^C NMR (101 MHz, CDCl_3_) *δ* 157.5, 155.2, 151.4, 142.8, 134.2, 131.9, 130.2, 130.1, 129.6, 129.2, 129.0, 128.5, 127.5, 110.7, 47.8, and 21.7. HRMS (ESI) calcd. for C_20_H_16_N_5_^+^ [M + H]^+^ 326.1400, found: 326.1397.

**4u**: Colorless crystals, mp: 38–40 °C. Yield: 46 mg, 42%. R*_f_* = 0.25 (PE/EtOAc = 30/1, *v*/*v*). ^1^H NMR (400 MHz, CDCl_3_) *δ* 8.73 (d, *J* = 8.4 Hz, 2H, ArH), 7.53–7.51 (m, 2H, ArH), 7.41–7.36 (m, 5H, PhH), and 5.63 (s, 2H, NCH_2_), 2.47 (s, 3H, CH_3_). ^13^C NMR (101 MHz, CDCl_3_) *δ* 158.4, 151.2, 144.4, 140.0, 133.4, 130.9, 130.6, 130.3, 129.9, 129.8, 129.5, 129.4, 128.7, 116.1, 110.0, 48.5, and 21.8. HRMS (ESI) calcd. for C_21_H_15_N_6_^+^ [M + H]^+^ 351.1353, found 351.1349.

#### 3.2.22. 9-Benzyl-6-(diethylamino)-9H-purine-2-carbonitrile (**2v’**)

**2v’**: Colorless crystals, mp: 88–90 °C. Yield: 10 mg, 16%, R*_f_* = 0.25 (PE/EtOAc = 5/1, *v*/*v*). ^1^H NMR (400 MHz, CDCl_3_) *δ* 7.79 (s, 1H, C8-H in purine), 7.39–7.32 (m, 5H, ArH), 5.34 (s, 2H, ArCH_2_), 4.19 (br s, 2H, NCH_2_), 3.76 (br s, 2H, NCH_2_), and 1.27 (t, *J* = 7.2 Hz, 6H, CH_3_). ^13^C NMR (101 MHz, CDCl_3_) *δ* 153.5, 150.0, 139.9, 137.8, 135.2, 129.1, 128.5, 128.0, 120.8, 117.2, 47.3, 43.9, 43.0, 14.1, and 12.8. HRMS (ESI) calcd. for C_17_H_19_N_6_^+^ [M + H]^+^ 307.1666, found 307.1670. [Note: The CH_2_ near the nitrogen atom was shown as a broad singlet in ^1^H NMR and ^13^C NMR because of both part double bond character in the amidine functionality and thus restricted rotation and the electric quadrupole of nitrogen-14. This structure was further verified by HSQC in Figure 1].

### 3.3. Synthesis of 9-Benzyl-6-(diethylamino)-9H-purine-8-carbonitrile (***2v***) and 9-Benzyl-6-(diethylamino)-N,N-diethyl-9H-purine-8-carboximidamide (***5f***)

Synthesis of **2v**: To a 10 mL vial were added purine **2f** (53.9 mg, 0.2 mmol), ethanol (2.0 mL), Et_2_NH (62 μL, 0.6 mmol), and Et_3_N (82 μL, 0.6 mmol) in order. The reaction mixture was stirred at room temperature for 2 h. The reaction system was filtered, and the filter residue was washed with EtOH/H_2_O followed by the infrared lamp drying to provide **2v** directly as white solids (51 mg, 83%). Mp: 94–96 °C. R*_f_* = 0.60 (PE/EtOAc = 3/1, *v*/*v*). ^1^H NMR (400 MHz, CDCl_3_) *δ* 8.43 (s, 1H, C2-H in purine), 7.45–7.31 (m, 5H, PhH), 5.48 (s, 2H, NCH_2_), 4.15 (br s, 2H, 1 CH_2_ in NEt_2_), 3.78 (br s, 2H, 1 CH_2_ in NEt_2_), and 1.28 (t, *J* = 6.9 Hz, 6H, 2CH_3_).^13^C NMR (101 MHz, CDCl_3_) *δ* 155.4, 154.1, 150.4, 134.8, 129.0, 128.6, 128.3, 121.7, 120.2, 111.4, 47.3, 44.1, 43.0, 13.8, and 12.6. ^1^H NMR (400 MHz, DMSO-*d*_6_) *δ* 8.39 (s, 1H, C2-H in purine), 7.36–7.30 (m, 5H, ArH), 5.52 (s, 2H, ArCH_2_), 4.09 (br s, 2H, NCH_2_), 3.73 (br s, 2H, NCH_2_), and 1.20 (br s, 6H, CH_3_). ^13^C NMR (101 MHz, DMSO-*d*_6_) *δ* 155.7, 153.9, 150.7, 135.9, 129.4, 128.7, 128.0, 122.4, 119.8, 111.9, 47.2, 44.00, 42.8, 14.2, and 12.9. HRMS (ESI) calcd. for C_17_H_19_N_6_^+^ [M + H]^+^ 307.1666, found: 367.1687. [Note: The CH_2_ near the nitrogen atom was shown as a broad singlet in ^1^H NMR and ^13^C NMR because of both part double bond character in the amidine functionality and thus restricted rotation and the electric quadrupole of nitrogen-14].

Synthesis of **5f**: To a 10 mL vial were added purine **2f** (53.9 mg, 0.2 mmol) and Et_2_NH (1 mL). The reaction mixture was then heated at 60 °C for 3 h under stirring. After cooling to room temperature, the solvent was removed in vacuo and the residue was purified through column chromatography with DCM/MeOH (25:1, *v*/*v*) as the eluent to obtain **5f** as colorless crystals (69 mg, 91%). Mp: 138–140 °C. R*_f_* = 0.20 (DCM/MeOH = 25/1, *v*/*v*). ^1^H NMR (400 MHz, CDCl_3_) *δ* 8.42 (s, 1H, C2-H in purine), 7.28–6.92 (m, 5H, ArH), 6.92 (br s, 1H, NH), 5.46 (s, 2H, NCH_2_), 3.97 (br s, 4H, NCH_2_), 3.09 (br s, 4H, NCH_2_), 1.28 (t, *J* = 7.2 Hz, 6H, CH_3_), and 1.08 (t, *J* = 7.2 Hz, 6H, CH_3_). ^13^C NMR (101 MHz, CDCl_3_) *δ* 157.2, 153.9, 153.3, 151.0, 141.6, 136.3, 128.6, 128.2, 128.0, 118.4, 46.3, 43.0, 42.3, 13.5, and 12.8. HRMS (ESI) calcd. for C_21_H_30_N_7_^+^ [M + H]^+^ 380.2557, found: 380.2590. [Note: The CH_2_ near the nitrogen atom was shown as a broad singlet in ^1^H NMR and ^13^C NMR because of both part double bond character in the amidine functionality and thus restricted rotation and the electric quadrupole of nitrogen-14].

### 3.4. Synthesis of 9-Benzyl-6-((4-methoxyphenyl)ethynyl)-9H-purine-8-carbonitrile (***6f***)

To a 10 mL vial were added **2f** (53.94 mg, 0.2 mmol), CuI (3.44 mg, 0.018 mmol), and (PPh_3_)_2_PdCl_2_ (4.2 mg, 0.006 mmol) and the whole system was exchanged with N_2_. Toluene (1 mL) and Et_3_N (0.028 mL, 0.2 mmol) were then injected in 5 min at below 25 °C. The whole system was then stirred overnight (~11 h) at room temperature. After filtering with the diatomite, the filtrate was dissolved in 5 mL of EtOAc, then the resulting solution was washed with saturated NH_4_Cl and NaCl solution. The organic phase was collected, dried over anhydrous Na_2_SO_4_, and filtered. After removing the solvent in vacuo, the residue was purified by silica gel column chromatography with PE and EtOAc (5:1, *v*/*v*) as the eluent to afford the product **6f** as yellow crystals. Yield: 38 mg, 52%, mp: 141–143 °C. R*_f_* = 0.41 (PE/EtOAc = 3/1, *v*/*v*). ^1^H NMR (400 MHz, CDCl_3_) *δ* 9.10 (s, 1H, C2-H in purine), 7.70 (d, *J* = 8.8 Hz, 2H, ArH), 7.47 (dd, *J* = 7.5, 2.2 Hz, 2H, ArH), 7.41–7.33 (m, 3H, ArH), 6.93 (d, *J* = 8.8 Hz, 2H, ArH), 5.62 (s, 2H, NCH_2_), and 3.86 (s, 3H, OCH_3_). ^13^C NMR (101 MHz, CDCl_3_) *δ* 161.5, 155.4, 150.4, 145.1, 134.8, 133.9, 133.1, 129.3, 129.1, 128.4, 128.3, 114.3, 112.7, 110.3, 102.2, 83.8, 55.4, and 48.0. HRMS (ESI) calcd. for C_22_H_16_N_5_O^+^ [M + H]^+^ 366.1350, found: 366.1356.

### 3.5. Synthesis of ***2u*** from ***2f***

To a 10 mL reaction tube were added **2f** (53.94 mg, 0.2 mmol), *p-*tolylboronic acid (33 mg, 0.24 mmol), Pd(PPh_3_)_4_ (15 mg, 0.01 mmol) and K_3_PO_4_ (212 mg, 1 mmol) and then a mixed solvent of DME:H_2_O = 10:1 (2 mL) was injected. The reaction system was stirred at 90 °C for 5 h. After cooling to room temperature, the reaction system was filtered, and the filtrate was dried over anhydrous Na_2_SO_4_. After removing the solvent in vacuo, the residue was purified by silica gel column chromatography with PE and EtOAc (10:1, *v*/*v*) as the eluent, to yield the **2u** as colorless crystals, 19 mg, 28%.

### 3.6. Synthesis of ***2t*** from ***2f***

To a 10 mL vial were added **2f** (53.9 mg, 0.2 mmol), NaOMe (8.0 mg, 0.3 mmol), and CuBr (3.0 mg, 0.02 mmol) in DMF (2 mL). The vial was capped and heated at 110 °C for 5 h. After cooling to room temperature, EtOAc (5 mL) was added and the whole system was washed with brine (5 mL × 3). The aqueous phase was then extracted with EtOAc (5 mL). The organic phase was collected and dried over anhydrous Na_2_SO_4_. After removing the solvent in vacuo, the residue was purified by silica gel column chromatography with PE and EtOAc (5:1, *v*/*v*) as the eluent to afford the **2t** as colorless crystals, 17 mg, 32%.

### 3.7. Synthesis of ***2s*** and Benzyl 9-Benzyl-6-(benzylthio)-2-cyano-9H-purine-8-carbimidothioate (***5s***) from ***2f***

In a 10 mL vial, **2f** (53.94 mg, 0.2 mmol) was dissolved into 2 mL of ethanol, and then benzylthiol (35 μL, 0.3 mmol) and Et_3_N (22 μL, 0.22 mol) were added in order. The resulting mixture was stirred at room temperature for 30 min. After removal of the solvent in vacuo, the residue was separated by column chromatography with PE and EtOAc (10:1, *v*/*v*) as the eluent to provide **2s** as colorless crystals (18 mg, 24%) and **5s** as colorless crystals (39 mg, 54%). **5s:** R*_f_* = 0.45 (PE/EtOAc = 5/1, *v*/*v*), mp: 110–112 °C. ^1^H NMR (400 MHz, CDCl_3_) *δ* 10.23 (s, 1H, NH), 8.78 (s, 1H, C2-H in purine), 7.46–7.44 (m, 2H, ArH), 7.37–7.17 (m, 13H, ArH), 5.94 (s, 2H, NCH_2_), 4.67 (s, 2H, SCH_2_), and 4.04 (s, 2H, SCH_2_). ^13^C NMR (101 MHz, CDCl_3_) *δ* 163.8, 162.0, 153.0, 150.4, 146.5, 137.2, 136.5, 134.1, 130.0, 129.1, 128.9, 128.6, 128.4, 127.8, 127.6, 127.5, 127.2, 47.3, 34.0, and 33.0. HRMS (ESI) calcd. for C_27_H_24_N_5_S_2_^+^ [M + H]^+^ 482.1468, found: 482.1468.

### 3.8. Synthesis of ***5s*** from ***2s***

To a 10 mL vial were added purine **2s** (71.49 mg, 0.2 mmol), ethanol (2.0 mL), benzyl thiol (27 μL, 0.24 mmol), and Et_3_N (23 μL, 0.24 mmol) in order. The reaction mixture was then stirred at room temperature for 1 h. After filtration and washing with H_2_O, the residue was dried under infrared lamp to provide **5s** (75 mg, 78%) as a white solid.

### 3.9. Synthesis of Methyl 9-Benzyl-6-(benzylthio)-9H-purine-8-carbimidate (***6s***) from ***2s***

In a dry flask **2s** (178.73 mg, 0.5 mmol) and NaOMe (2.7 mg, 0.05 mmol) were dissolved in anhydrous methanol (5 mL). The reaction mixture was stirred at room temperature for 4 h. The reaction mixture was filtered, and the filter residue was washed with H_2_O followed by the infrared lamp drying to provide **6s** directly as white solids (155 mg, 80%). R*_f_* = 0.15 (PE/EtOAc = 5/1, *v*/*v*), mp: 105–106 °C. ^1^H NMR (400 MHz, CDCl_3_) *δ* 9.14 (s, 1H, NH), 8.81 (s, 1H, C2-H in purine), 7.48–7.46 (m, 2H, ArH), 7.33–7.14 (m, 8H, ArH), 5.74 (s, 2H, NCH_2_), 4.68 (s, 2H, SCH_2_), and 3.95 (s, 3H, Ome). ^13^C NMR (101 MHz, CDCl_3_) *δ* 161.9, 159.1, 153.1, 150.1, 142.8, 137.1, 136.4, 129.9, 129.1, 128.7, 128.5, 127.9, 127.4, 127.0, 53.8, 48.0, and 32.9. HRMS (ESI) calcd. for C_21_H_20_N_5_OS^+^ [M + H]^+^ 390.1383, found: 390.1383.

### 3.10. Synthesis of 2-(9-Benzyl-6-(benzylthio)-9H-purin-8-yl)-4,4-dimethyl-4,5-dihydrooxazole (***7s***) from ***6s***, and One-Pot Procedure from ***2s***

Procedure from **6s**: **6s** (155 mg,0.4 mmol), 2-amino-2-methylpropan-1-ol (38 μL, 0.4 mmol), TsOH·H_2_O (7.6 mg, 0.04 mmol) were dissolved in toluene (5 mL). The reaction mixture was refluxed with a Dean–Stark apparatus for 12 h. After the reaction was cooled to room temperature, the solvent was removed in vacuo and the residue was purified through a silica gel flash column chromatography (PE/EtOAc = 6/1, *v*/*v*) to afford **7s** as a white solid (120 mg, 70%). R*_f_* = 0.35 (PE/EtOAc = 5/1, *v*/*v*), mp: 105–107 °C. ^1^H NMR (400 MHz, CDCl_3_) *δ* 8.81 (s, 1H, C2-H in purine), 7.46–7.44 (m, 2H, ArH), 7.33–7.23 (m, 8H, ArH), 6.01 (s, 2H, NCH_2_), 4.65 (s, 2H, SCH_2_), 4.12 (s, 2H, OCH_2_), and 1.36 (s, 6H, 2CH_3_). ^13^C NMR (101 MHz, CDCl_3_) *δ* 162.4, 153.8, 153.0, 150.0, 140.3, 137.3, 136.4, 130.3, 129.1, 128.43, 128.37, 128.1, 127.8, 127.2, 78.7, 69.2, 47.2, 32.8, and 28.2. HRMS (ESI) calcd. for C_24_H_24_N_5_OS^+^ [M + H]^+^ 430.1696, found: 430.1696.

Procedure from **2s**: **2s** (178.73 mg, 0.5 mmol) was dissolved in anhydrous methanol (5 mL) in a flame-dried flask equipped with a magnetic stir bar. NaOMe (2.7 mg, 0.05 mmol) was added, and the reaction mixture was stirred at room temperature for 4 h. The solvent was removed in vacuo and toluene (5 mL) was added along with 2-amino-2-methylpropan-1-ol (47.5 μL, 0.5 mmol) and TsOH·H_2_O (9.5 mg, 0.05 mmol). The reaction system was refluxed with a Dean–Stark apparatus overnight (~12 h). After cooling to r. t., the solvent was removed in vacuo and the residue was purified through silica gel column chromatography (PE/EtOAc = 6/1, *v*/*v*) to afford **7s** as a white solid (162 mg, 75%).

### 3.11. Synthesis of Ethyl 9-Benzyl-6-(benzylthio)-9H-purine-8-carbimidate (***6s’***) from ***2s***

In a vial **2s** (71.49 mg, 0.2 mmol) and NaOH (1.0 mg, 0.02 mmol) were dissolved in a mixture of EtOH/H_2_O = 7/3 (2 mL). The resulting solution was stirred at room temperature for 4 h. After filtration and washing with water, the solid was dried under the infrared lamp to afford **6s’** as a white solid (68 mg, 84%). R*_f_* = 0.2 (PE/EtOAc = 5/1, *v*/*v*), mp: 56–58 °C. ^1^H NMR (400 MHz, CDCl_3_) *δ* 9.13 (s, 1H, NH), 8.79 (s, 1H, C2-H in purine), 7.48–7.46 (m, 2H, ArH), 7.33–7.09 (m, 8H, ArH), 5.78 (s, 2H, NCH_2_), 4.68 (s, 2H, SCH_2_), 4.38 (q, *J* = 7.2 Hz, 2H, OCH_2_), and 1.30 (t, *J* = 7.2 Hz, 3H, CH_3_). ^13^C NMR (101 MHz, CDCl_3_) *δ* 161.8, 159.4, 153.0, 150.2, 143.0, 137.1, 136.4, 129.9, 129.1, 128.7, 128.5, 127.8, 127.3, 126.6, 63.0, 47.9, 33.0, and 13.9. HRMS (ESI) calcd. for C_22_H_22_N_5_OS^+^ [M + H]^+^ 404.1540, found: 404.1546.

### 3.12. Synthesis of 9-Benzyl-6-(benzylthio)-9H-purine-8-carboxamide (***8s***) from ***2s***

In a vial **2s** (71.49 mg, 0.2 mmol) and NaOH (1.0 mg, 0.02 mmol) were dissolved in a mixture of THF/H_2_O = 7/3 (2 mL) and the reaction mixture was stirred at room temperature for 4 h. The reaction system was filtered, and filter residue was washed with H_2_O followed by the drying under infrared lamp to provide **8s** as white solids (62 mg, 83%). R*_f_* = 0.15 (PE/EtOAc = 5/1, *v*/*v*), mp: 184–186 °C. ^1^H NMR (400 MHz, DMSO-*d*_6_) *δ* 8.86 (s, 1H, C2-H in purine), 8.37 (s, 1H in NH_2_), 8.04 (s, 1H in NH_2_), 7.48–7.46 (m, 2H, ArH), 7.33–7.23 (m, 8H, ArH), 5.86 (s, 2H, NCH_2_), and 4.70 (s, 2H, SCH_2_). ^13^C NMR (101 MHz, DMSO-*d*_6_) *δ* 161.4, 160.7, 153.3, 150.3, 144.3, 138.1, 137.4, 129.5, 129.4, 129.00, 128.97, 128.1, 127.8, 127.7, 47.4, and 32.34. HRMS (ESI) calcd. for C_20_H_18_N_5_OS^+^ [M + H]^+^ 376.1227, found: 376.1237.

### 3.13. Synthesis of Dimethyl 3-(9-Benzyl-6-(benzylthio)-9H-purin-8-yl)isothiazole-4,5-dicarboxylate (***10s***) from ***2s***

To a pre-dried reaction tube were sequentially added **2s** (143 mg, 0.4 mmol), dimethyl 1,2,3-thiadiazole-4,5-dicarboxylate (40.4 mg, 0.2 mmol), [Rh(COD)Cl]_2_ (5 mg, 0.01 mmol), DPPF (13 mg, 0.024 mmol), and CsI (6 mg, 0.01 mmol). The reaction tube was evacuated (<1 mmHg) and refilled with nitrogen three times. Anhydrous chlorobenzene (2 mL) was injected via a syringe. The reaction tube was placed in a metal module pre-heated to 130 °C and stirred at 130 °C for 2 h. After cooling to r. t., the solution was purified by column chromatography on silica gel (PE/EtOAc = 8/1, *v*/*v*) to provide **10s** as colorless crystals (106 mg, 99%). R*_f_* = 0.35 (PE/EtOAc = 5/1, *v*/*v*), mp: 119–120 °C. ^1^H NMR (400 MHz, CDCl_3_) *δ* 8.79 (s, 1H, C2-H in purine), 7.48–7.46 (m, 2H, ArH), 7.34–7.20 (m, 8H, ArH), 6.07 (s, 2H, NCH_2_), 4.67 (s, 2H, SCH_2_), 4.09 (s, 3H, CH_3_), and 3.95 (s, 3H, CH_3_). ^13^C NMR (101 MHz, CDCl_3_) *δ* 163.8, 161.7, 158.7, 155.6, 154.5, 152.8, 150.2, 143.7, 137.2, 136.1, 136.0, 130.3, 129.2, 128.5, 127.8, 127.4, 127.3, 53.7, 53.3, 47.4, and 32.9. HRMS (ESI) calcd. for C_26_H_22_N_5_O_4_S_2_^+^ [M + H]^+^ 532.1108, found: 532.1108.

## 4. Conclusions

A direct C-H cyanation of purines was developed from corresponding purines with TMSCN via sequential Tf_2_O activation, nucleophilic addition, and a base-mediated detrifluoromethanesulfination. The current transformation showed a good tolerance of variety of functional groups, including allyl, alkynyl, ketone, ester, nitro, etc. The cyanation occurred highly regioselectively at the 8-position of purines with various substituents, including electron-withdrawing 6-chloro, aryl, and electron-donating alkoxy and alkylthio groups. Strong electron-donating diethylamino can reverse the regioselectivity of purine, and 2-cyano-6-diethylamino purine is obtained. Therefore, both 8- and 2-cyano 6-diethylaminopurines can be obtained from corresponding 6-chloropurine from differently substituted purines. Moreover, 6-chloro-8-cyanopurines are also versatile substrates for the further transformations because both of 6-chloro and nitrile are good electrophilic sites for various nucleophiles. For the further synthetic applications of nitriles, cyanopurines were easily converted into numerous derivatives, such as amide, oxazolines, isothiazoles, imidates, imidothioates, and imidamides.

## Figures and Tables

**Table 1:** Optimization of the cyanation of purine **1a**.

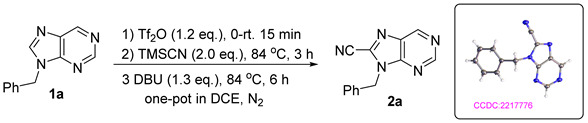		
Entry	Variation from the Standard Conditions	2a (%) ^a^
1	-	54
2	TFA, TfOH, or TMSOTf instead of Tf_2_O	0
3	BF_3_^.^OEt_2_ or HBF_4_ instead of Tf_2_O	0
4	Ac_2_O, Ts_2_O, or Boc_2_o instead of Tf_2_O	0
5	THF or MeCN as the solvent	0
6	DCM as the solvent	30
7	PhCl as the solvent	47
8	PhMe as the solvent	49
9	CHCl_3_ as the solvent	50
10	Py instead of DBU	40
11	Et_3_N instead of DBU	40
12	DABCO instead of DBU	26
13	*N*-Methylmorpholine (NMM) instead of DBU	47
14	Quinuclidine instead of DBU	26
15	DBN instead of DBU	41

^a^ Yield of isolated **2a**.

## Data Availability

The data presented in this study are available on request from the corresponding author.

## Supplementary Materials

The following supporting information can be downloaded at: https://www.mdpi.com/article/10.3390/molecules28030914/s1, X-ray crystal data, and copies of ^1^H-NMR and ^13^C-NMR spectra of unknown compounds are included in the Supporting Information.
