# Supplementary material for: Direct Regioselective C-H Cyanation of Purines
Source: Molecules. 2023 Jan 17;28(3):914. doi: 10.3390/molecules28030914 (PMC9920237; doi:10.3390/molecules28030914)

Electronic Supporting Information

Direct Regioselective C-H Cyanation of Purines

Luyong Li †, Jie Hu †, Yuqing Fu, Xiaolin Shi, Hongguang Du and Jiaxi Xu *, Ning Chen *

Department of Organic Chemistry, College of Chemistry, Beijing University of Chemical Technology, #15 Beisanhuan East Road, Chaoyang District, Beijing 100029, China

* Correspondence: jxxu@mail.buct.edu.cn (J.X.); chenning@mail.buct.edu.cn (N.C.)

† These authors contributed equally to this work.

Contents

1. The single crystal data of **2a** [CCDC: 2217776]……………………………………….2

2. Optimization of the reactions……………………………………………………………3

3. Copies of ^{1}H and ^{13}C-NMR spectra of cyanopurines **2** ……………………………4

4. The structural identification of **2v** and **2v′**……………………………………………25

5. Copies of ^{1}H and ^{13}C-NMR spectra of **3r, 4u, 5f, 5s & 6f** ……………………………29

6. Copies of ^{1}H and ^{13}C-NMR spectra of **6s, 6s′, 7s, 8s &10s.** …………………………34

1. The single crystal data of **2a** [CCDC: 2217776]

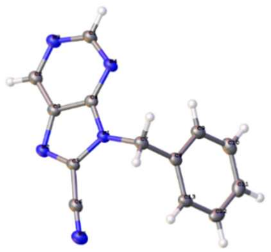

Crystal Data.

$C_{13}H_9N_5$, *M* =235.25, triclinic, *a* = 7.2138(13) Å, *b* = 7.7046(14) Å, *c* = 11.7853(19) Å, *α* = 104.439(15)°, *β* = 93.907(14)°, *γ* = 116.014(17)°, *U* = 558.08(17) Å^3, *T* = 113.50(14), space group P-1 (no. 2), *Z* = 2, μ(Mo K*α*) = 0.091, 3968 reflections measured, 2193 unique (R_{int} = 0.0299) which were used in all calculations. The final $wR(F_2)$ was 0.1088 (all data).

Table S1: Structure refinement for **2a**

Identification code	**2a**
Empirical formula	$C_{13}H_9N_5$
Formula weight	235.25
Temperature / K	113.50(14)
Crystal system	triclinic
Space group	P-1
a / Å, b / Å, c / Å	7.2138(13), 7.7046(14), 11.7853(19)
α/°, β/°, γ/°	104.439(15), 93.907(14), 116.014(17)
Volume / Å^3	558.08(17)
Z	2
ρ_{calc} / mg mm^{-3}	1.400
μ / mm^{-1}	0.091
F(000)	244
Crystal size / mm^3	0.54 × 0.28 × 0.21
2Θ range for data collection	6.12 to 51.98°
Index ranges	-8 ≤ h ≤ 8, -9 ≤ k ≤ 9, -14 ≤ l ≤ 14
Reflections collected	3968
Independent reflections	2193[R(int) = 0.0299 (inf-0.9Å)]
Data/restraints/parameters	2193/0/163
Goodness-of-fit on F^2	1.072
Final R indexes [I>2σ (I) i.e. F_o>4σ (F_o)]	R_1 = 0.0452, wR_2 = 0.0993
Final R indexes [all data]	R_1 = 0.0571, wR_2 = 0.1088
Largest diff. peak/hole / e Å^{-3}	0.240/-0.281
Flack Parameters	N
Completeness	0.9991

2. Optimization of the reactions

2.1 The screening of reactant loading

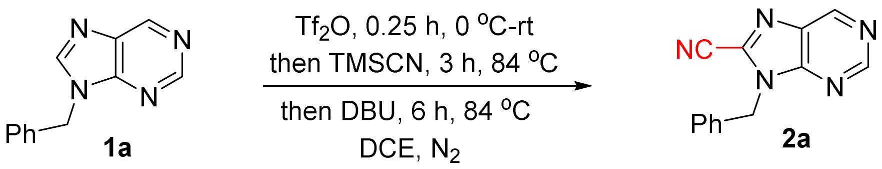

entry	Tf_2O (equiv)	TMSCN (equiv)	DBU (equiv)	**2a** (%)[(a)]
1	1.2	2.0	1.3	53
1	1.5	2.0	1.7	43
2	2.0	2.0	2.2	43
3	1.2	3.0	1.3	38

Reaction conditions: Tf_2O added to substrate **1a** (0.2 mmol) in DCE (2 mL) at 0 °C under N_2, stirred 1 h, then TMSCN added warmed to 84 °C for 3 h, and DBU added, warmed to 84 °C for 6 h before quench with $NaHCO_3$. [a.] All yields are obtained from isolated compounds.

2.2 The screening of the reaction time

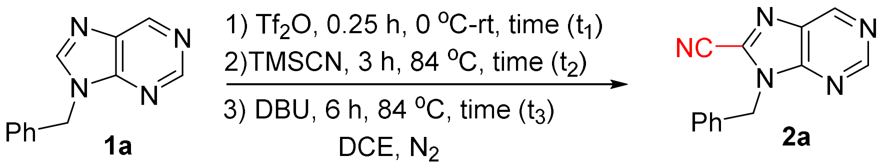

entry	t_1/h	t_2/h	t_3/h	**2a**%[(a)]
1	0.25	3	6	54
2	0.5	3	6	47
3	0.75	3	6	50
4	1	3	6	53
5	1.25	3	6	45
6	1.5	3	6	45
7	0.25	0	6	41
8	0.25	1	6	47
9	0.25	2	6	48
10	0.25	4	6	43
11	0.25	3	2	43
12	0.25	3	4	47
13	0.25	3	6	54
14	0.25	3	8	36
15	0.25	3	10	43

Reaction conditions: Tf_2O (1.2 eq) added to **1a** (0.2 mmol) in DCE (2 mL) at 0 °C under N_2, stirred t_1 (h), then TMSCN (2.0 eq) added warmed to 84 °C for t_2 (h), and DBU (1.3 eq) added, warmed to 84 °C for t_3 (h) before quench with $NaHCO_3$. [a.] All yields are obtained from isolated compounds.

3. Copies of ¹H and ¹³C-NMR spectra of cyanopurines 2

9-Benzyl-9H-purine-8-carbonitrile (**2a**)

^{1}H NMR (400 MHz, $CDCl_3$)

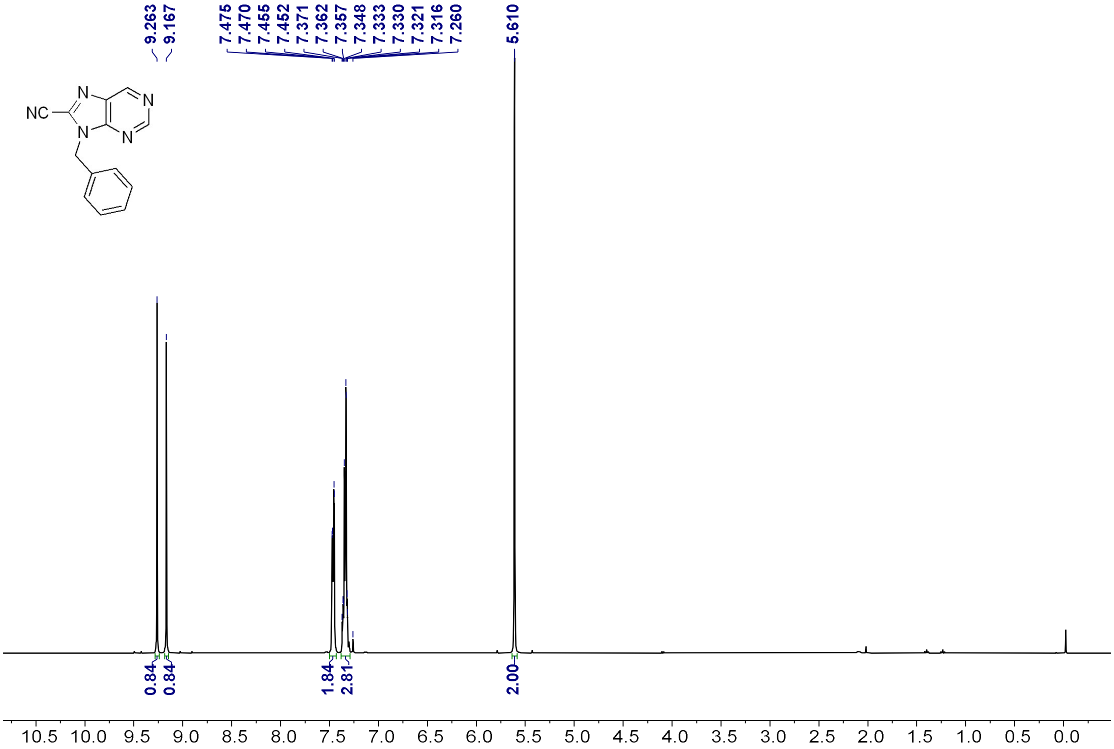

^{13}C NMR (101 MHz, $CDCl_3$)

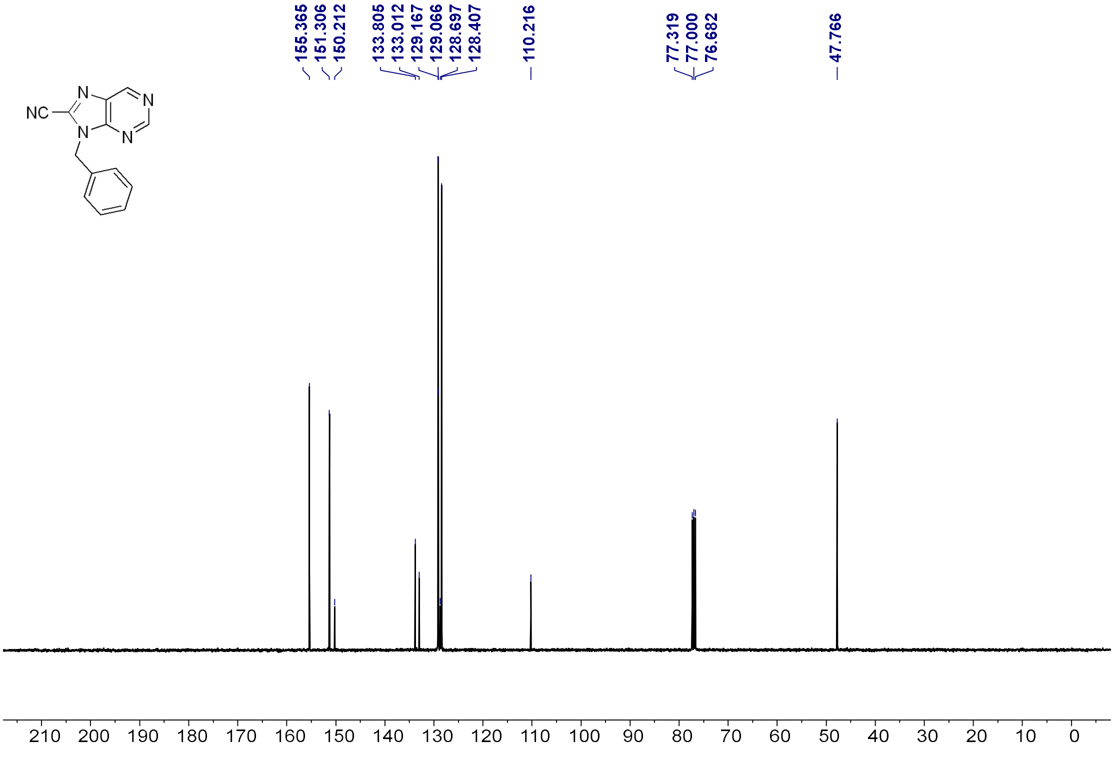

6-chloro-9-propyl-9H-purine-8-carbonitrile (**2b**)

^{1}H NMR (400 MHz, $CDCl_3$)

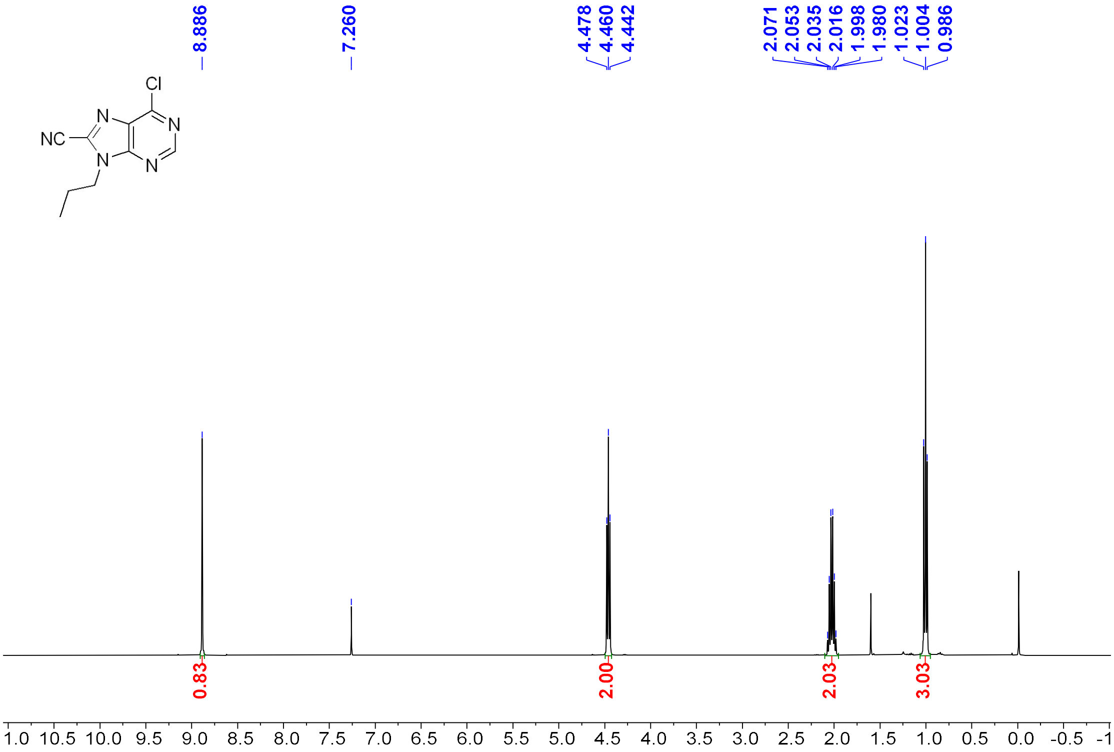

^{13}C NMR (101 MHz, $CDCl_3$)

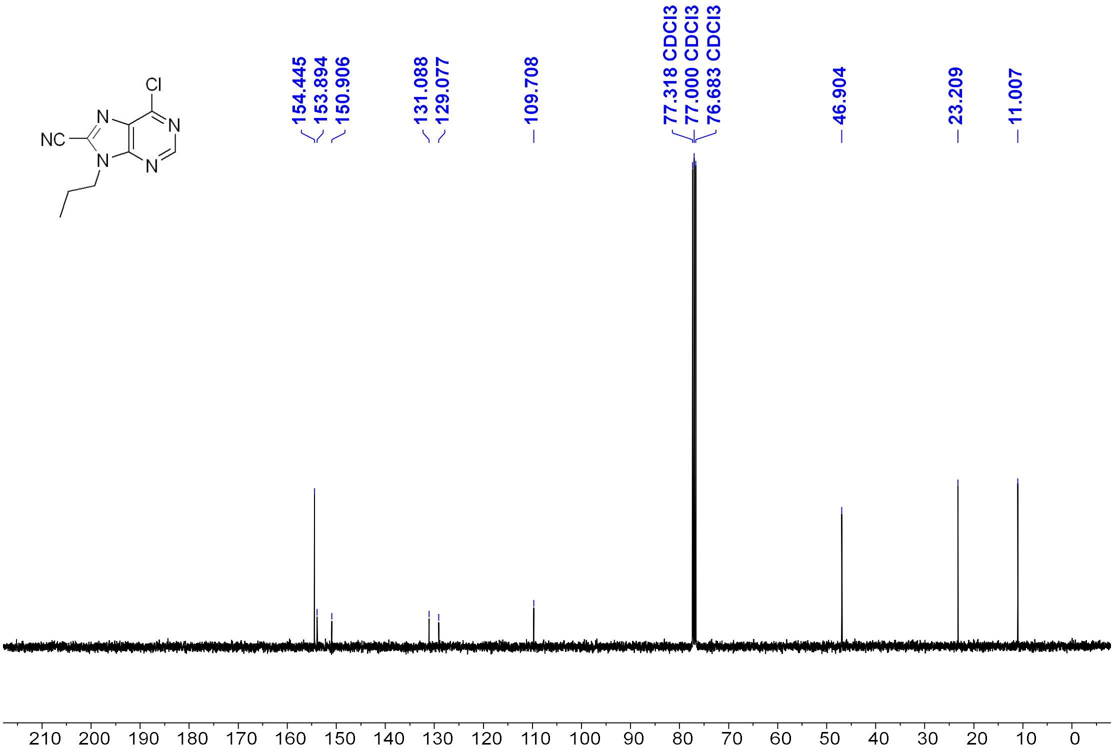

6-chloro-9-isopropyl-9H-purine-8-carbonitrile (**2c**)

^{1}H NMR (400 MHz, $CDCl_3$)

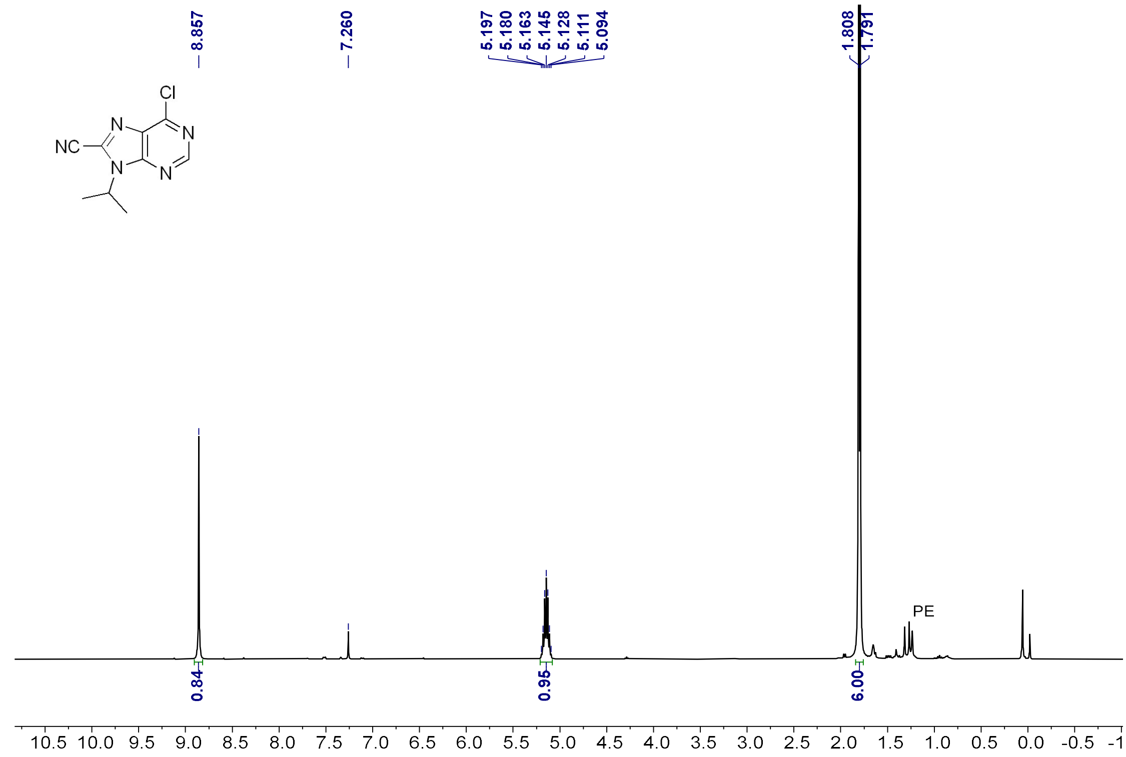

^{13}C NMR (101 MHz, $CDCl_3$)

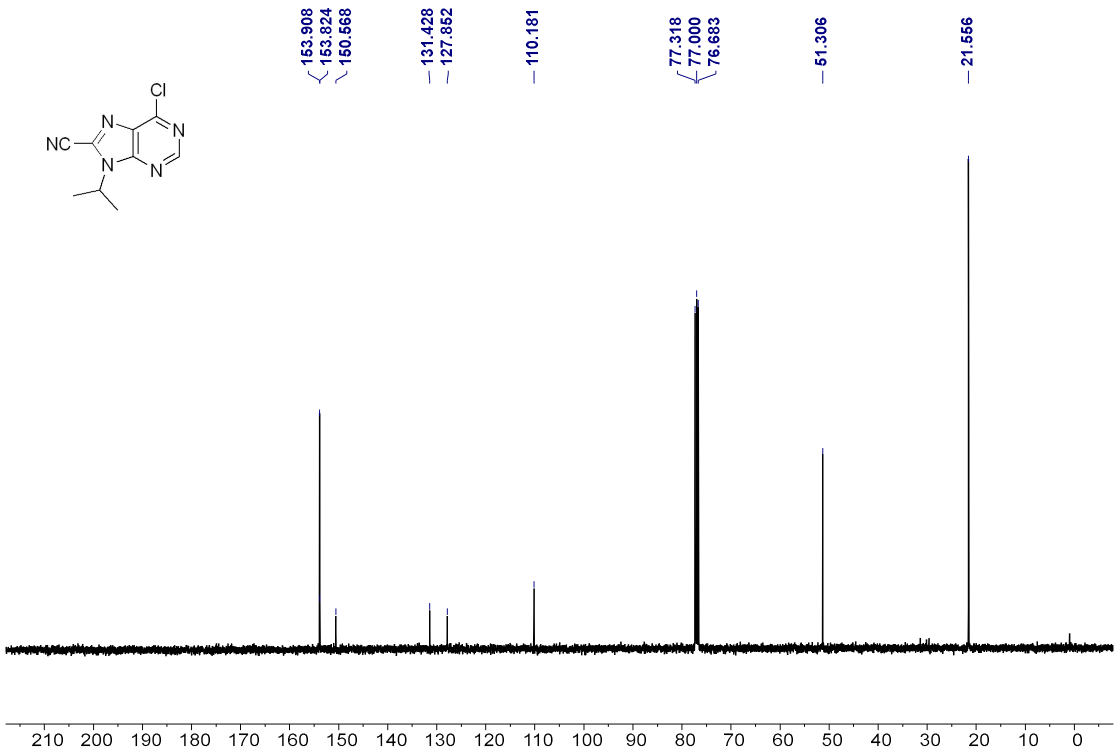

9-allyl-6-chloro-9H-purine-8-carbonitrile (**2d**)

^{1}H NMR (400 MHz, CDCl$_3$

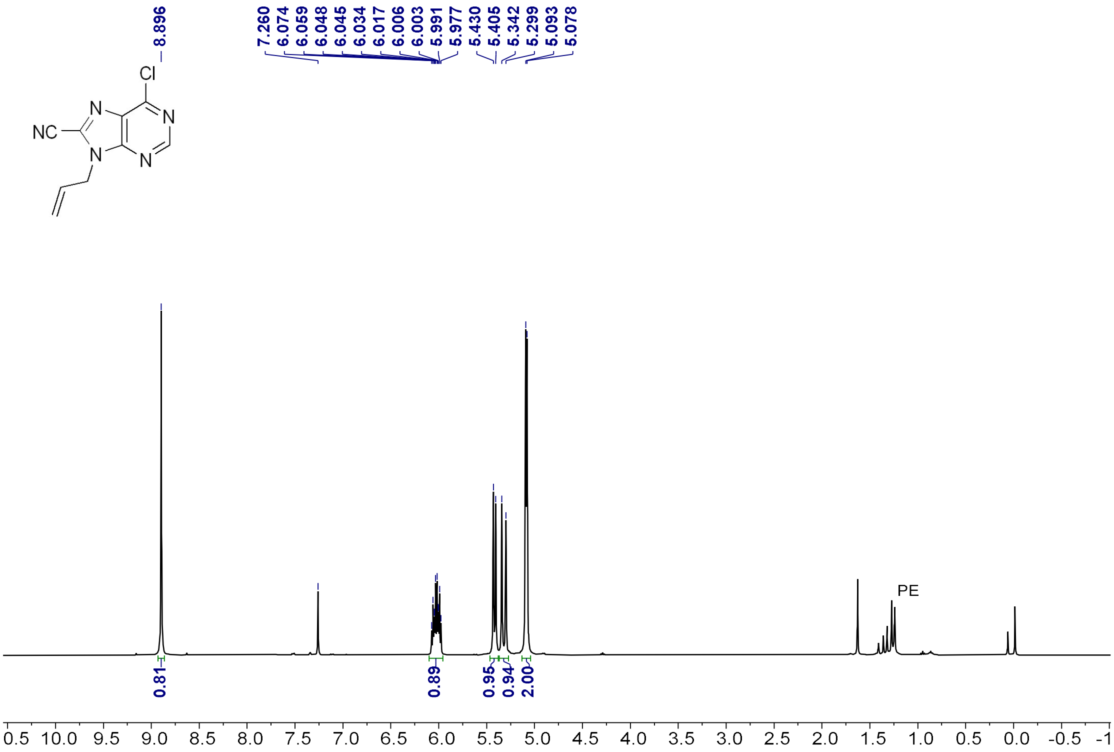

^{13}C NMR (101 MHz, CDCl$_3$)

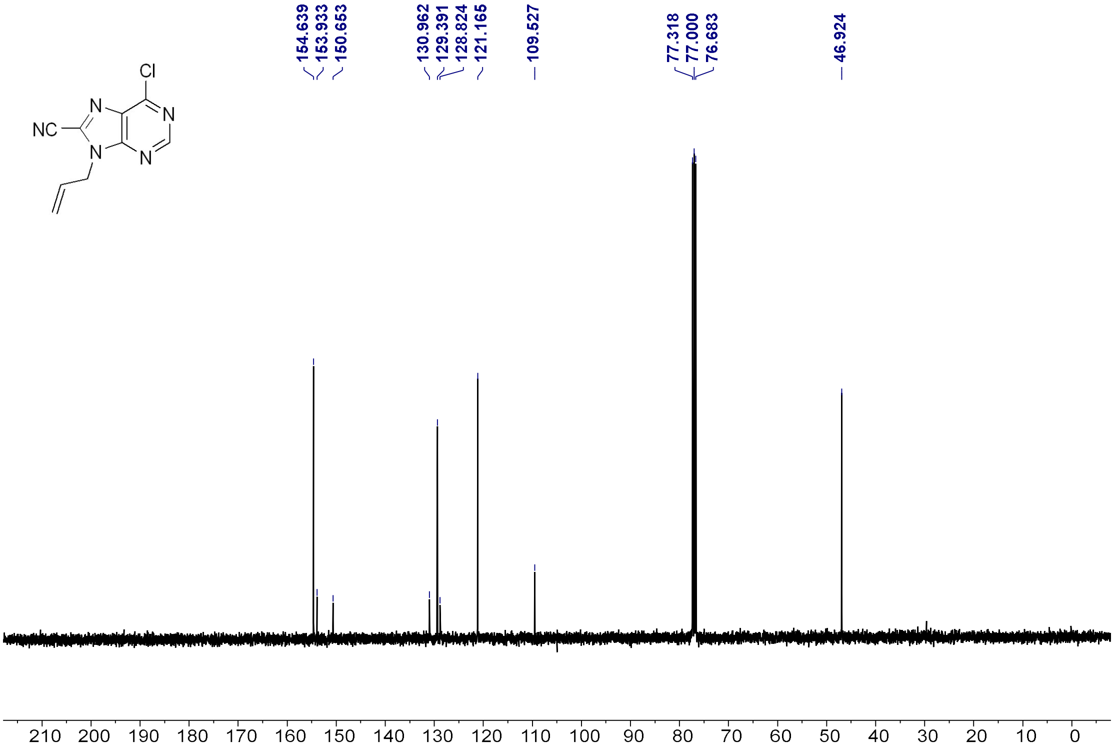

6-Chloro-9-(propargyl)-9H-purine-8-carbonitrile (**2e**)

^{1}H NMR (400 MHz, $CDCl_3$)

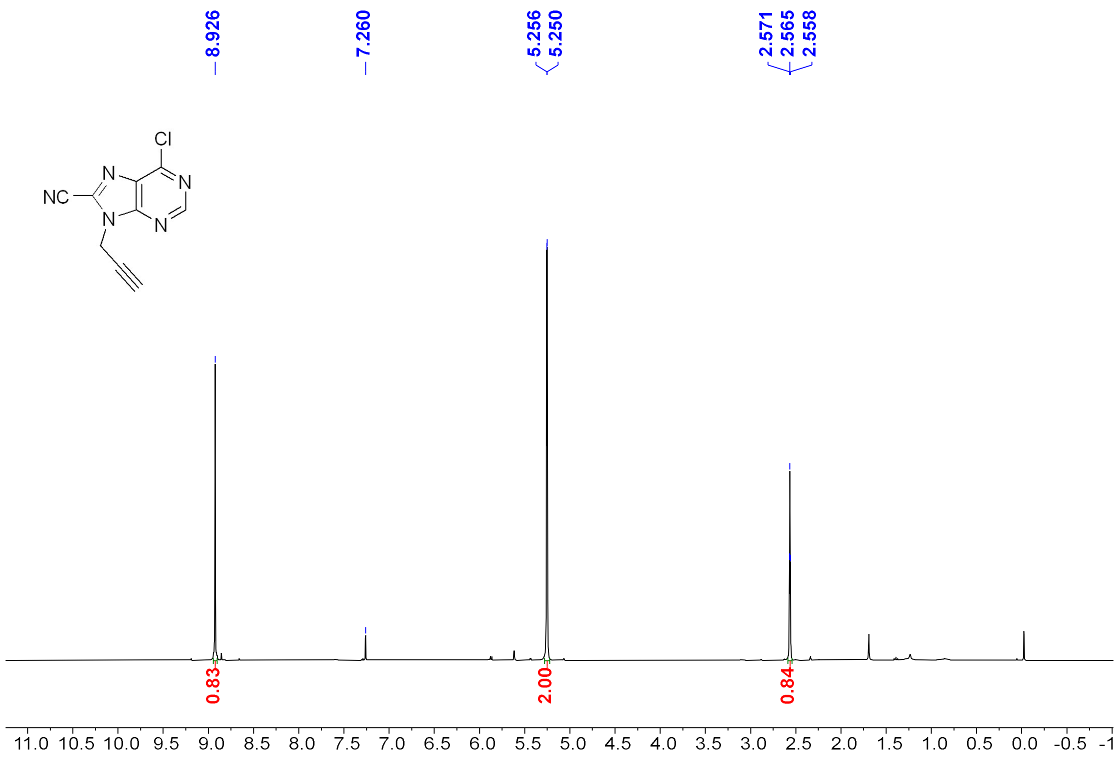

^{13}C NMR (101 MHz, $CDCl_3$)

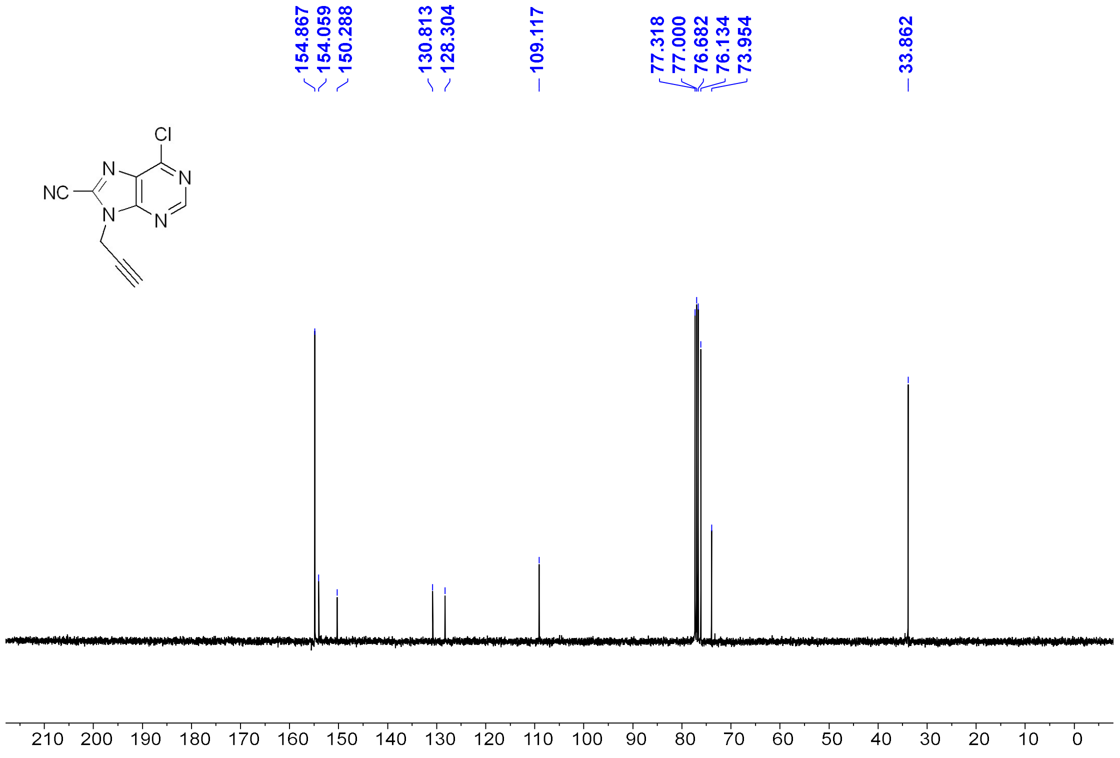

9-benzyl-6-chloro-9H-purine-8-carbonitrile (**2f**)

^{1}H NMR (400 MHz, $CDCl_3$)

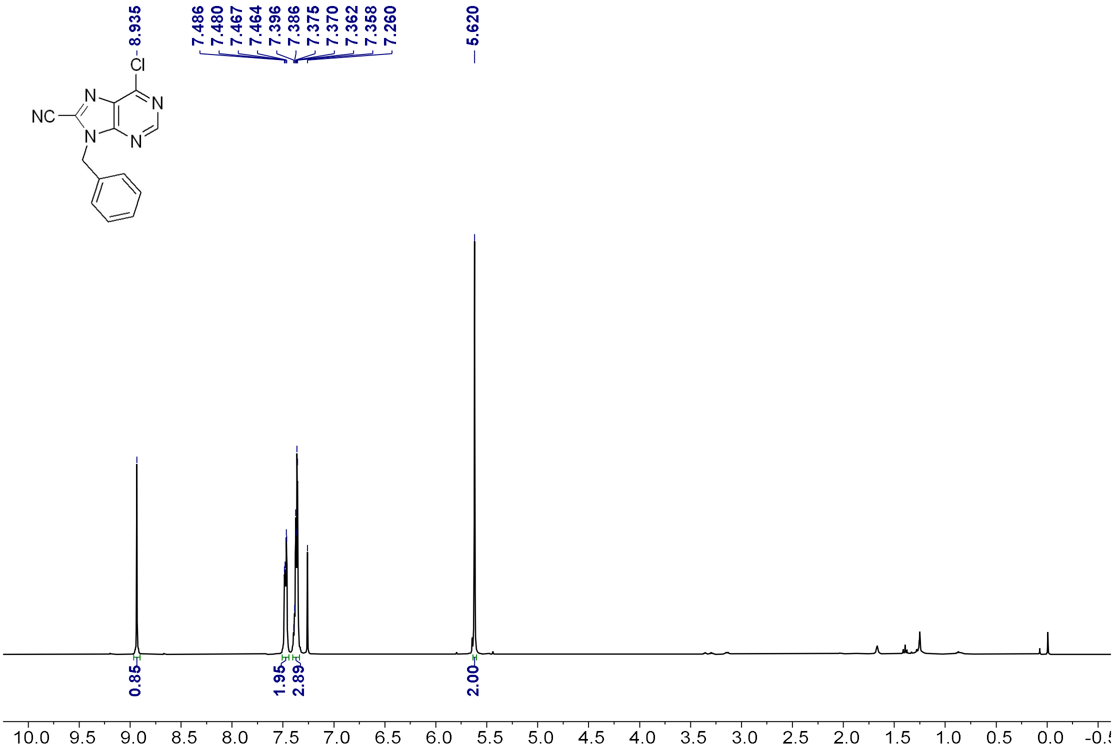

^{13}C NMR (101 MHz, $CDCl_3$)

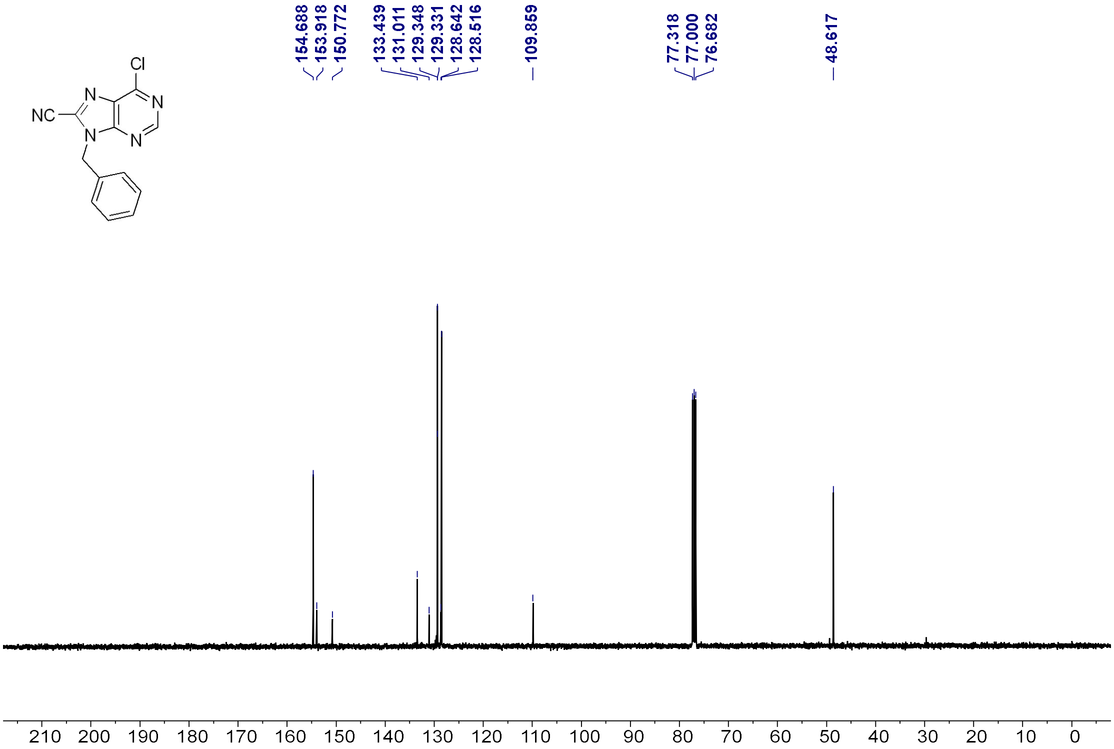

6-chloro-9-(4-methylbenzyl)-9H-purine-8-carbonitrile (**2g**)

^{1}H NMR (400 MHz, $CDCl_3$)

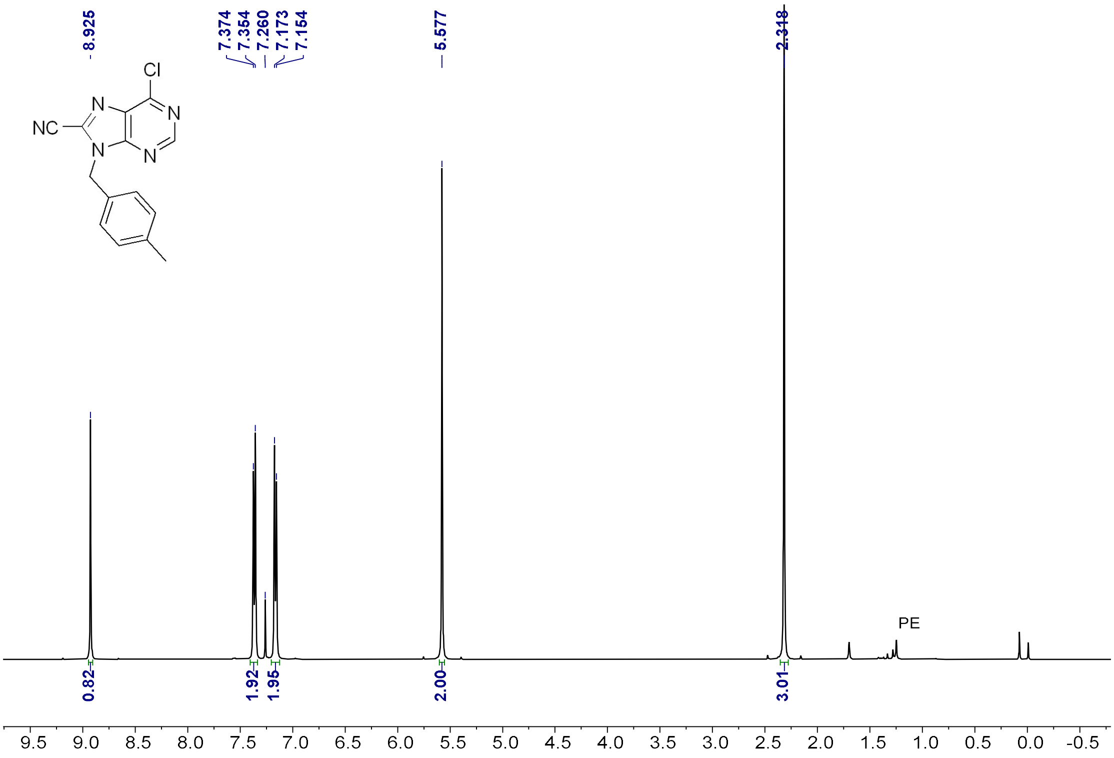

^{13}C NMR (101 MHz, $CDCl_3$)

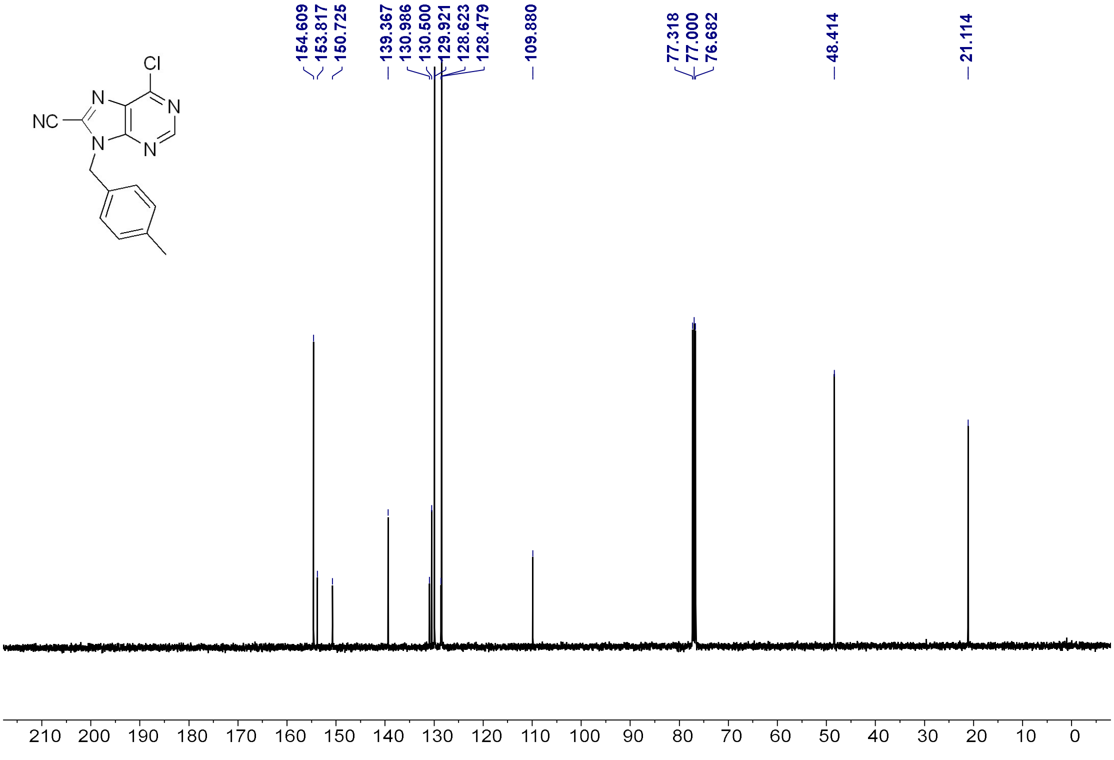

6-chloro-9-(4-nitrobenzyl)-9H-purine-8-carbonitrile (**2h**)

^{1}H NMR (400 MHz, DMSO-*d*$_6$)

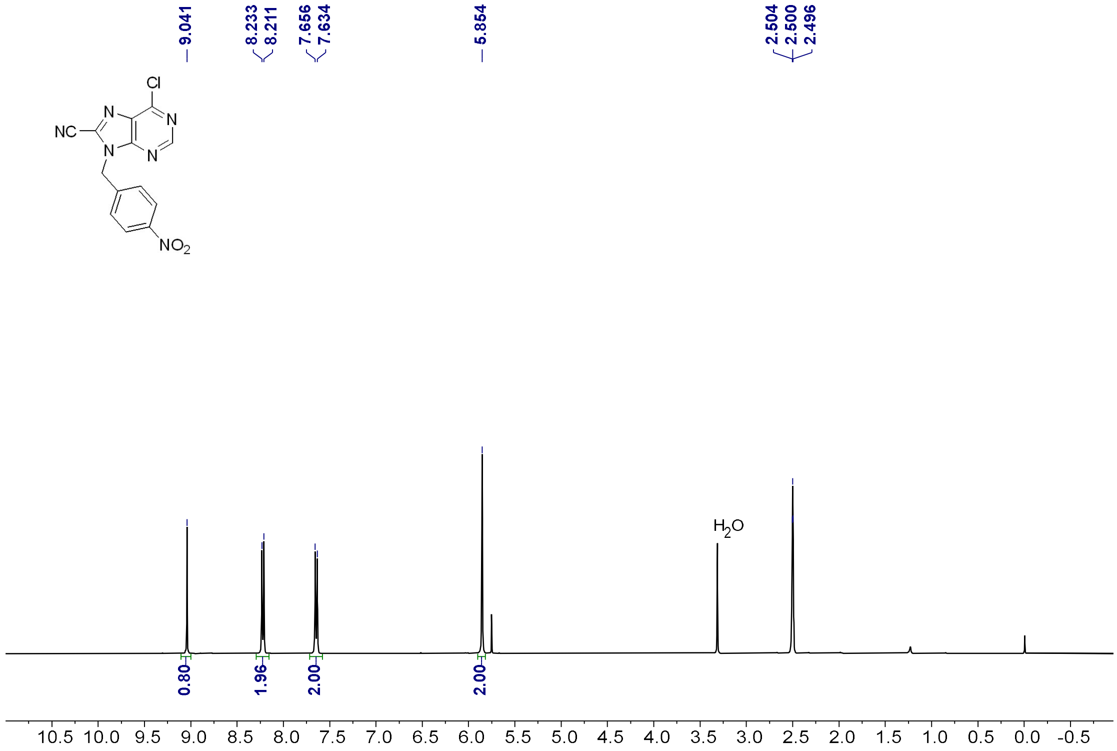

^{13}C NMR (101 MHz, DMSO-*d*$_6$)

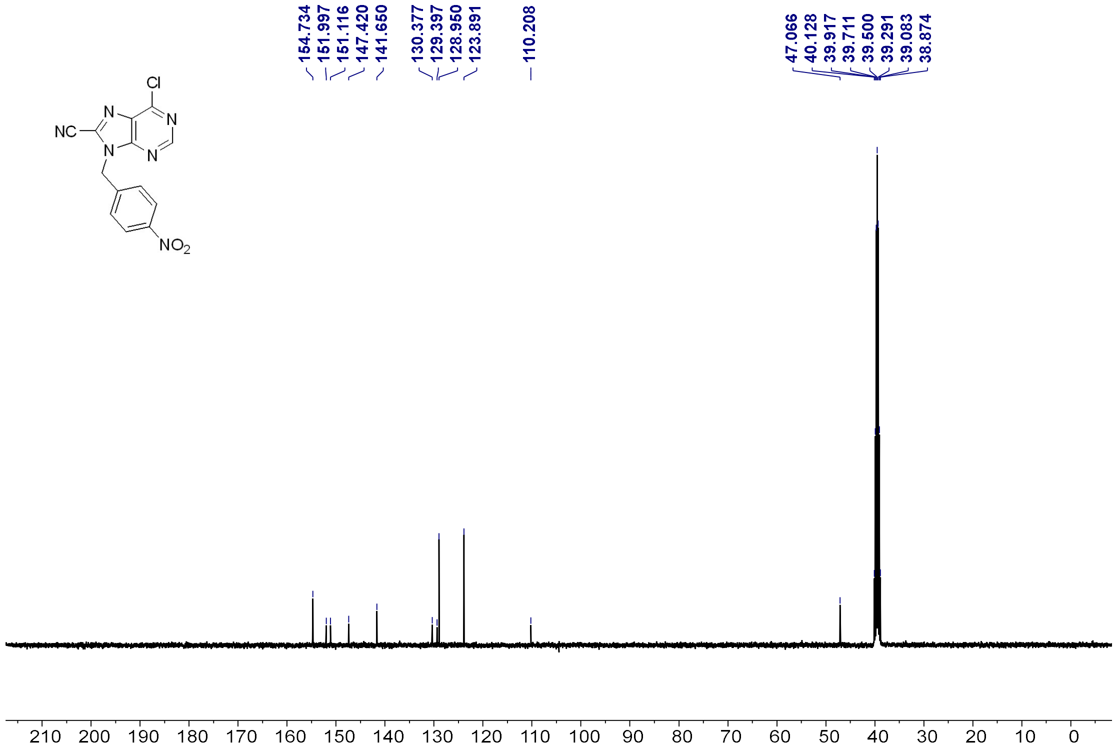

6-chloro-9-(4-methoxybenzyl)-9H-purine-8-carbonitrile (**2i**)

^{1}H NMR (400 MHz, $CDCl_3$)

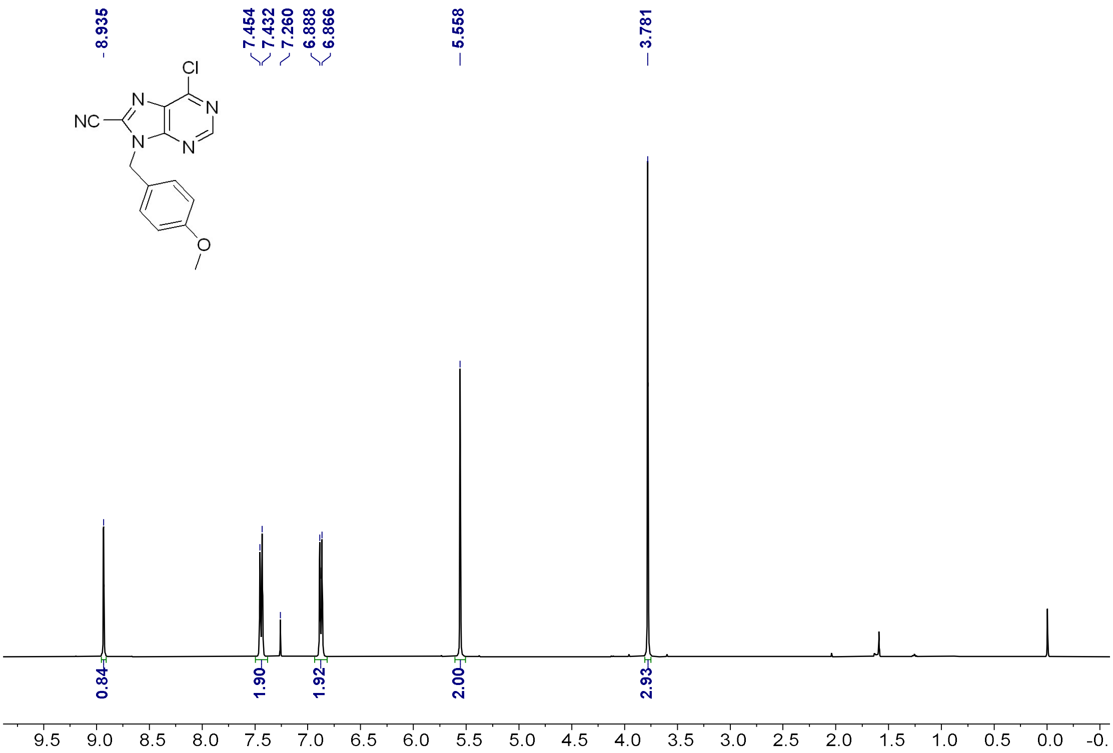

^{13}C NMR (101 MHz, $CDCl_3$)

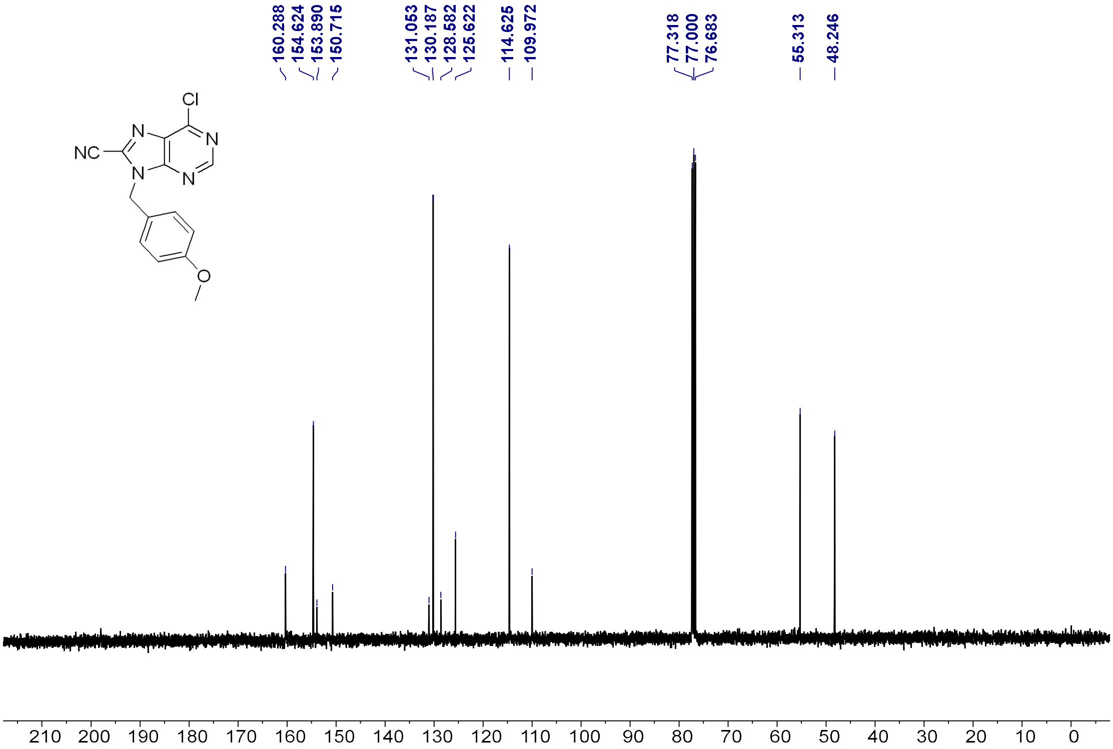

Ethyl-2-(6-chloro-8-cyano-9H-purin-9-yl) acetate (**2j**)

^{1}H NMR (400 MHz, $CDCl_3$)

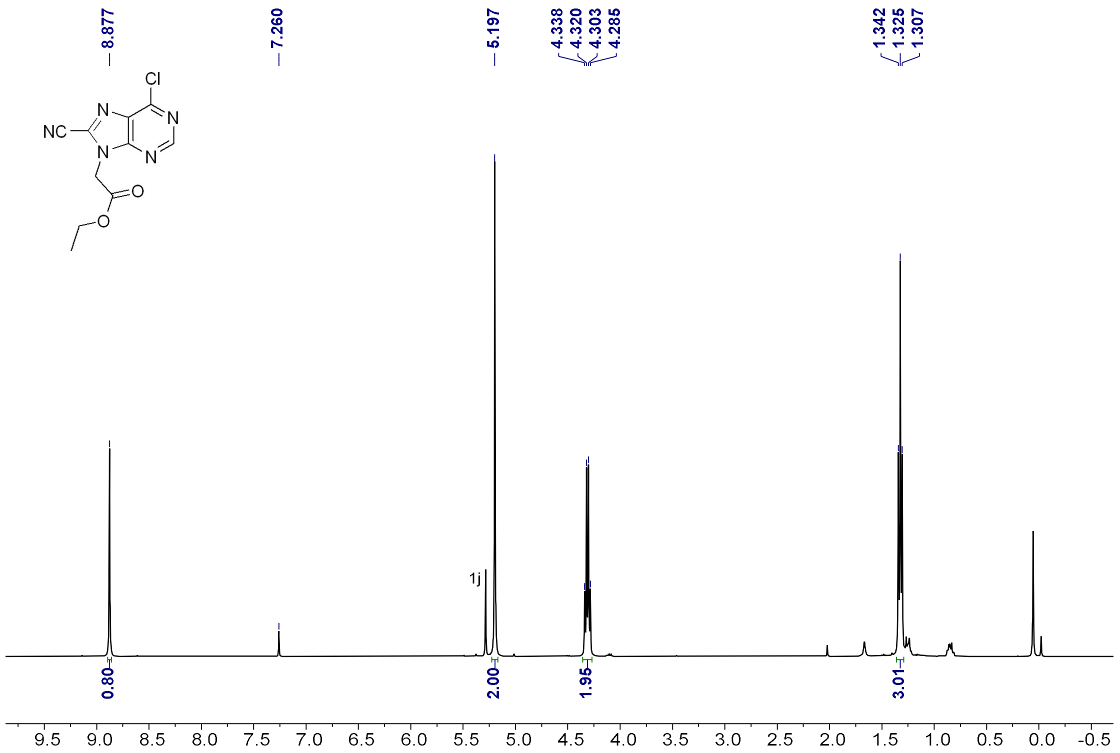

^{13}C NMR (101 MHz, $CDCl_3$)

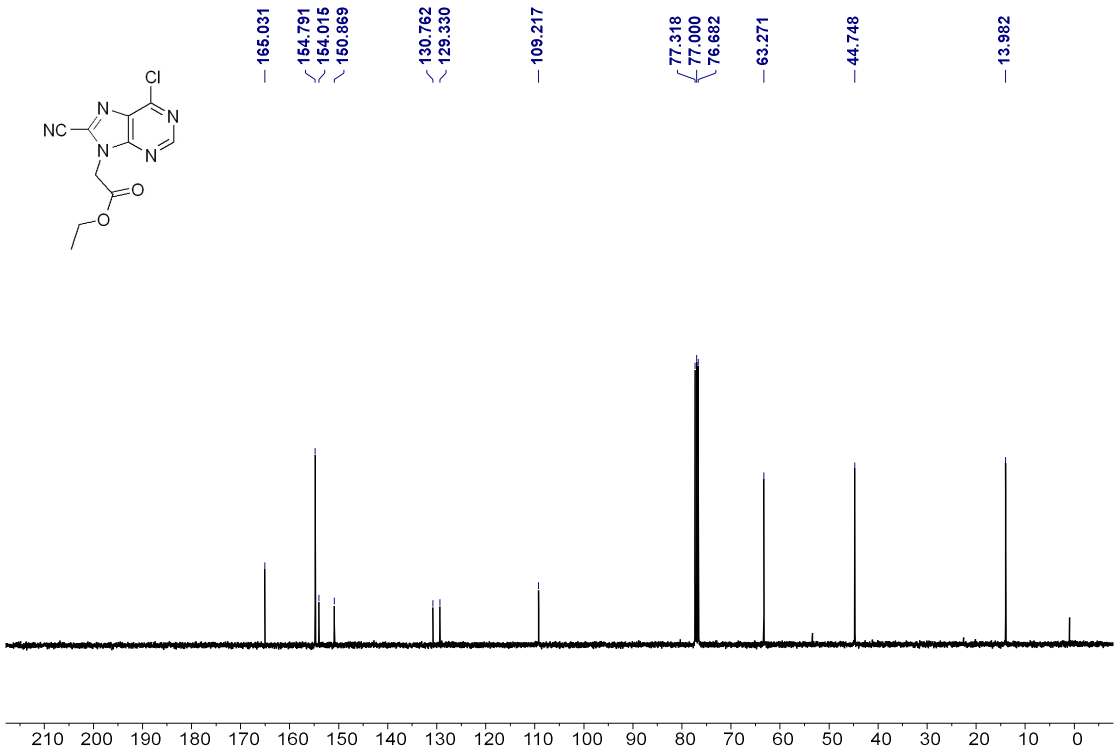

6-chloro-9-(2-(4-chlorophenyl)-2-oxoethyl)-9H-purine-8-carbonitrile (**2k**)

^{1}H NMR (400 MHz, $CDCl_3$)

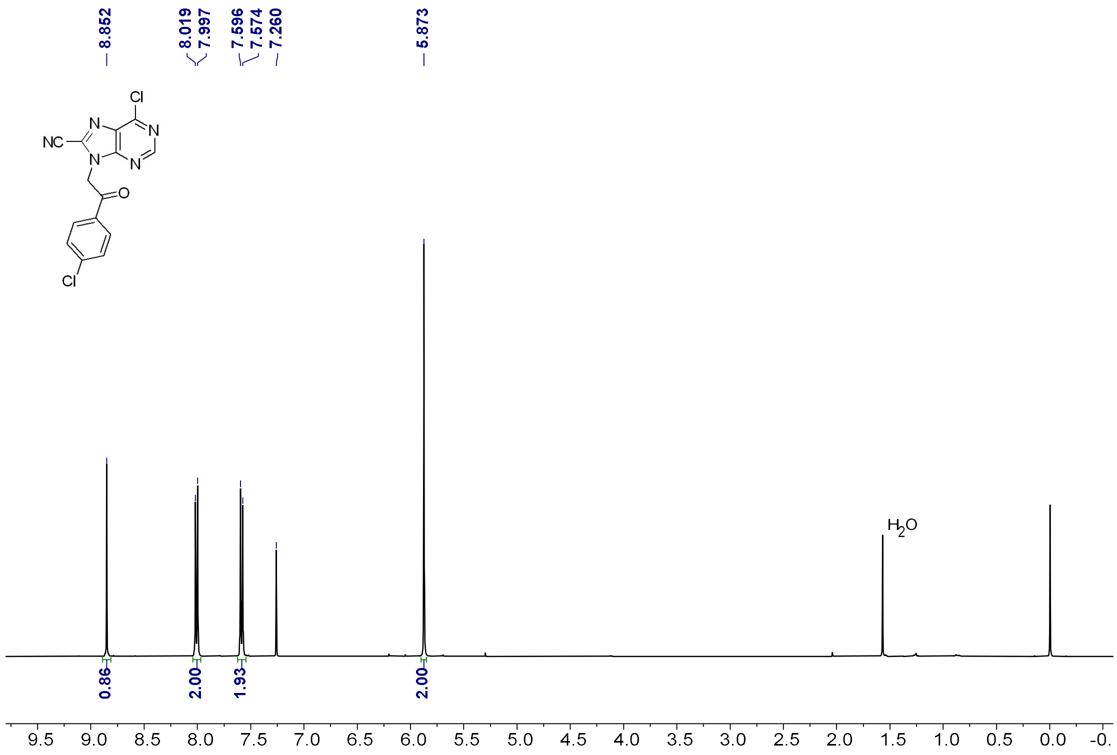

^{13}C NMR (101 MHz, $CDCl_3$)

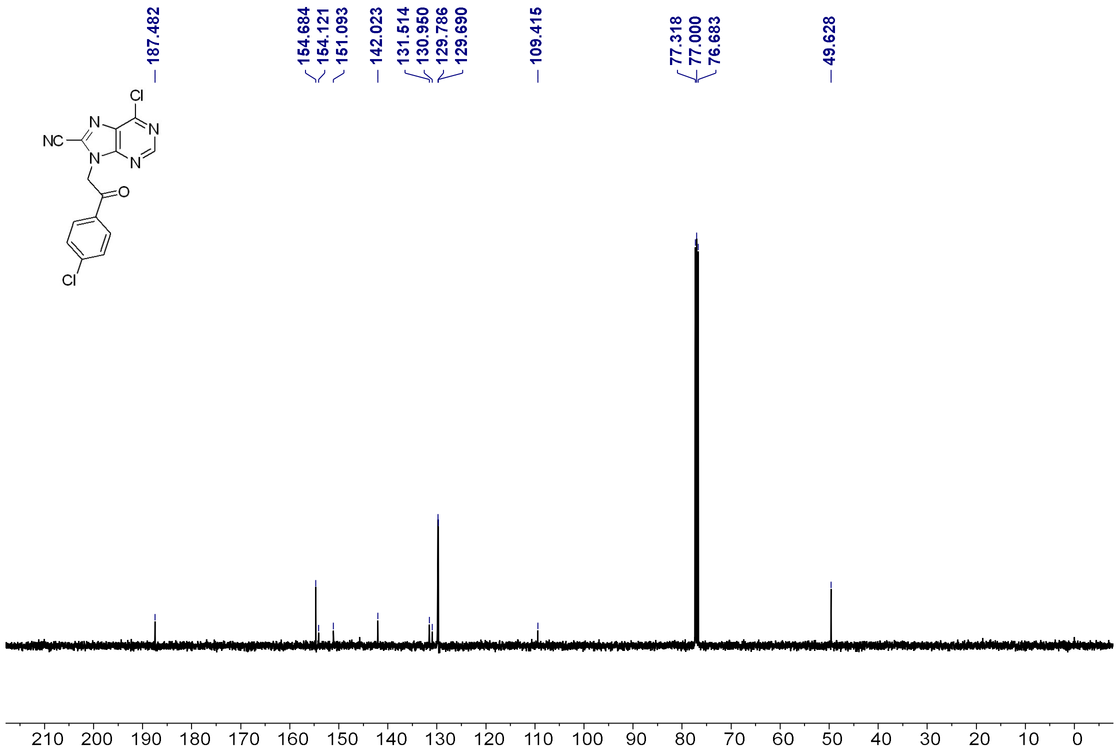

6-chloro-9-phenyl-9H-purine-8-carbonitrile (**2l**)

^{1}H NMR (400 MHz, $CDCl_3$)

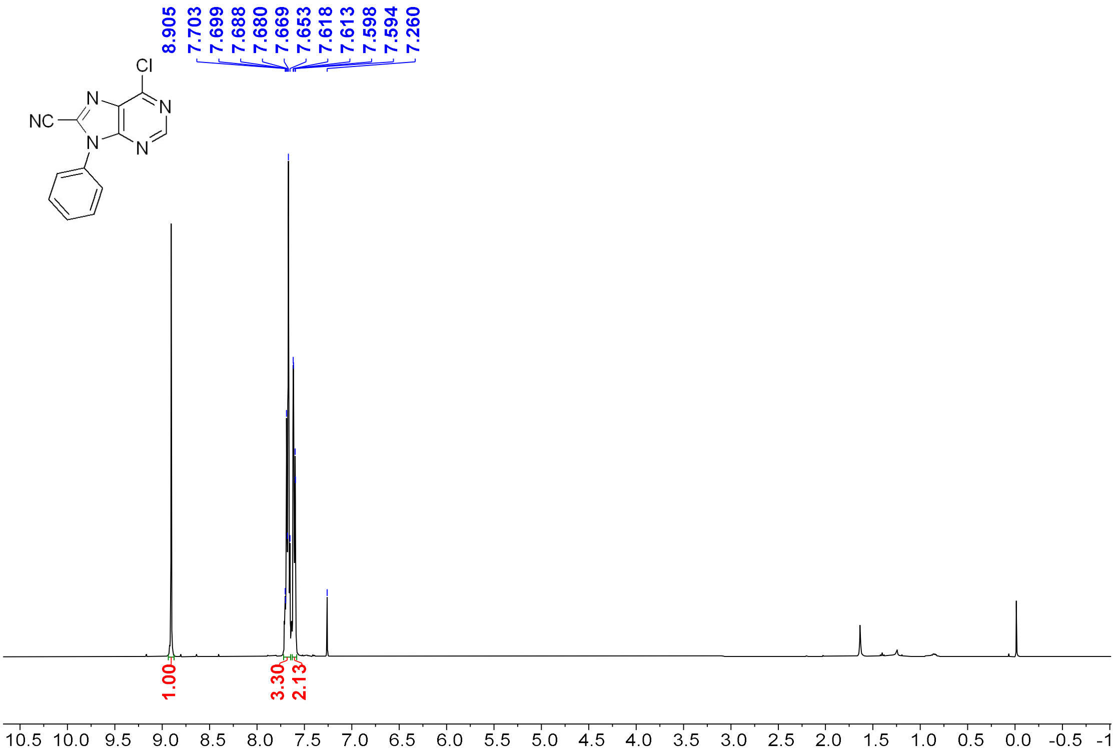

^{13}C NMR (101 MHz, $CDCl_3$)

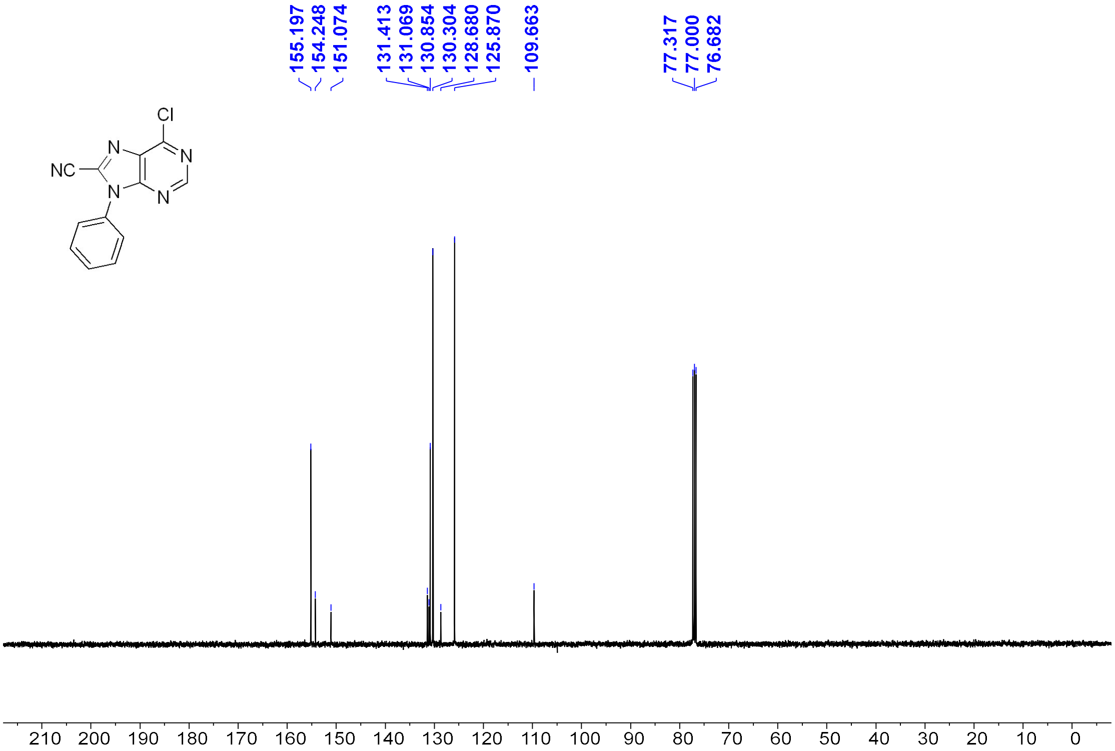

6-chloro-9-(p-tolyl)-9H-purine-8-carbonitrile (**2m**)

^{1}H NMR (400 MHz, $CDCl_3$)

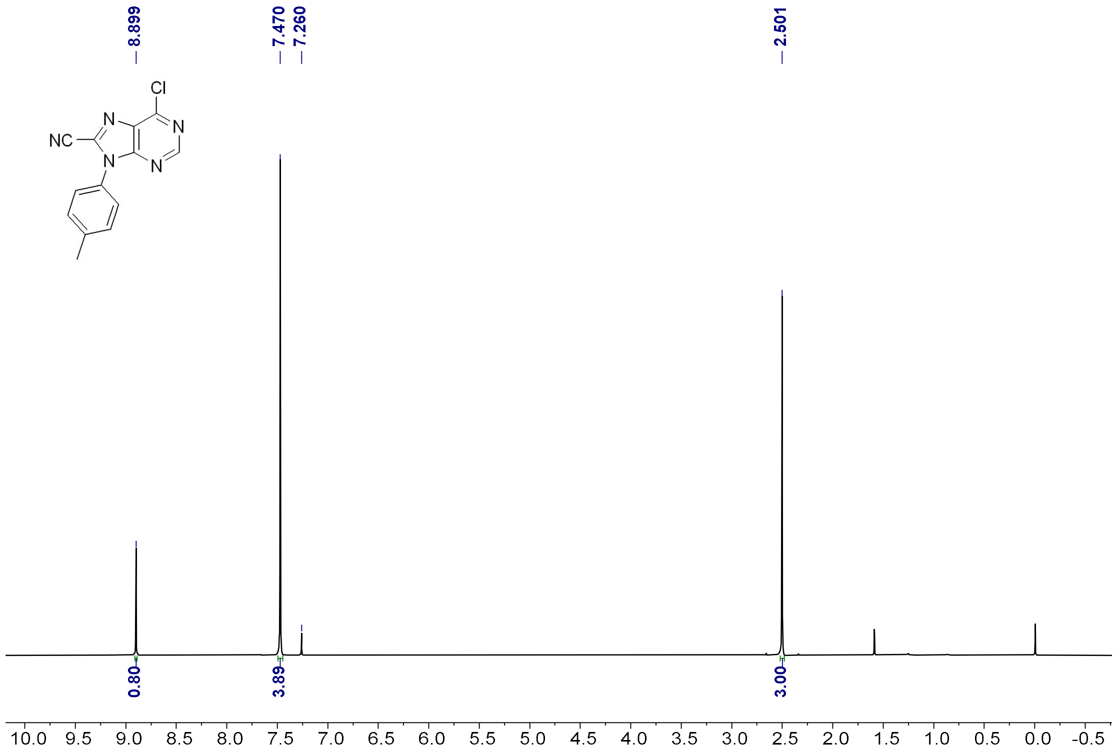

^{13}C NMR (101 MHz, $CDCl_3$)

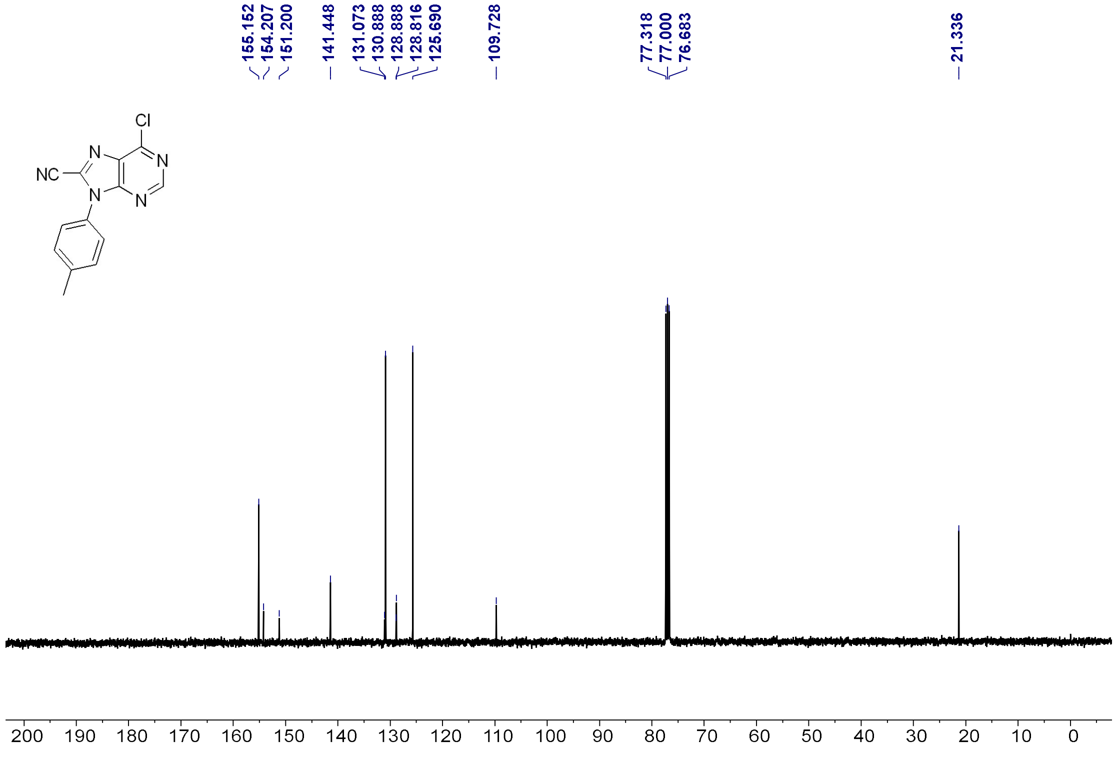

6-chloro-9-(4-methoxyphenyl)-9H-purine-8-carbonitrile (***2n***)

^{1}H NMR (400 MHz, $CDCl_3$)

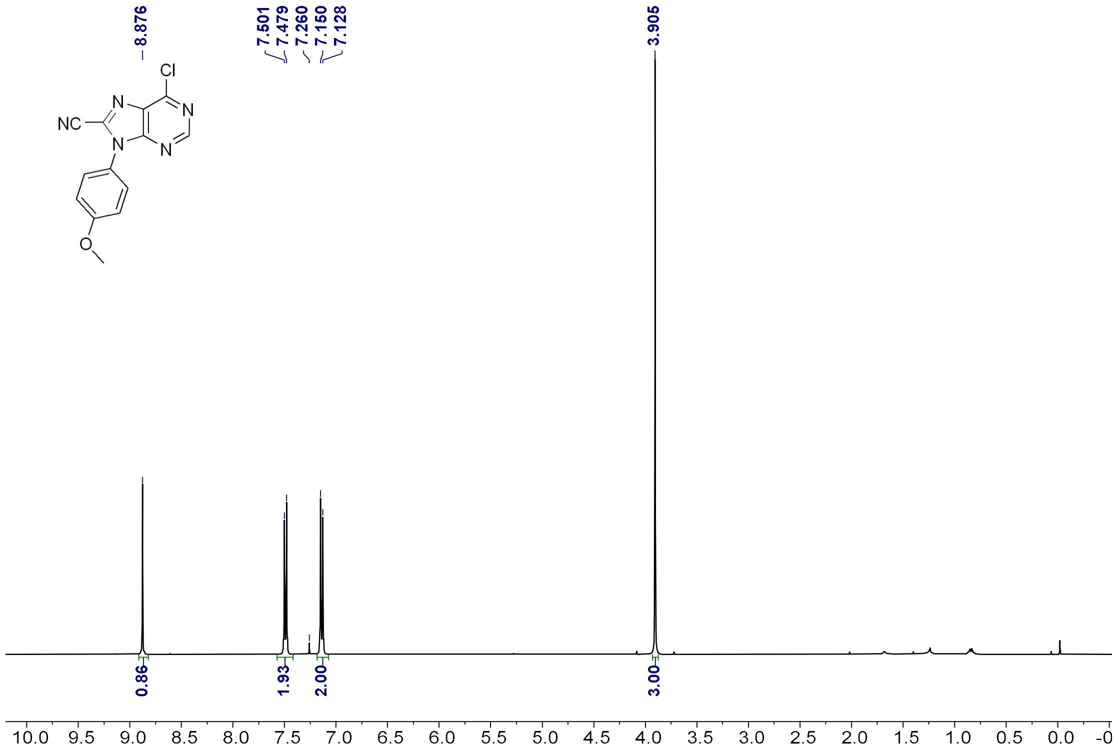

^{13}C NMR (101 MHz, $CDCl_3$)

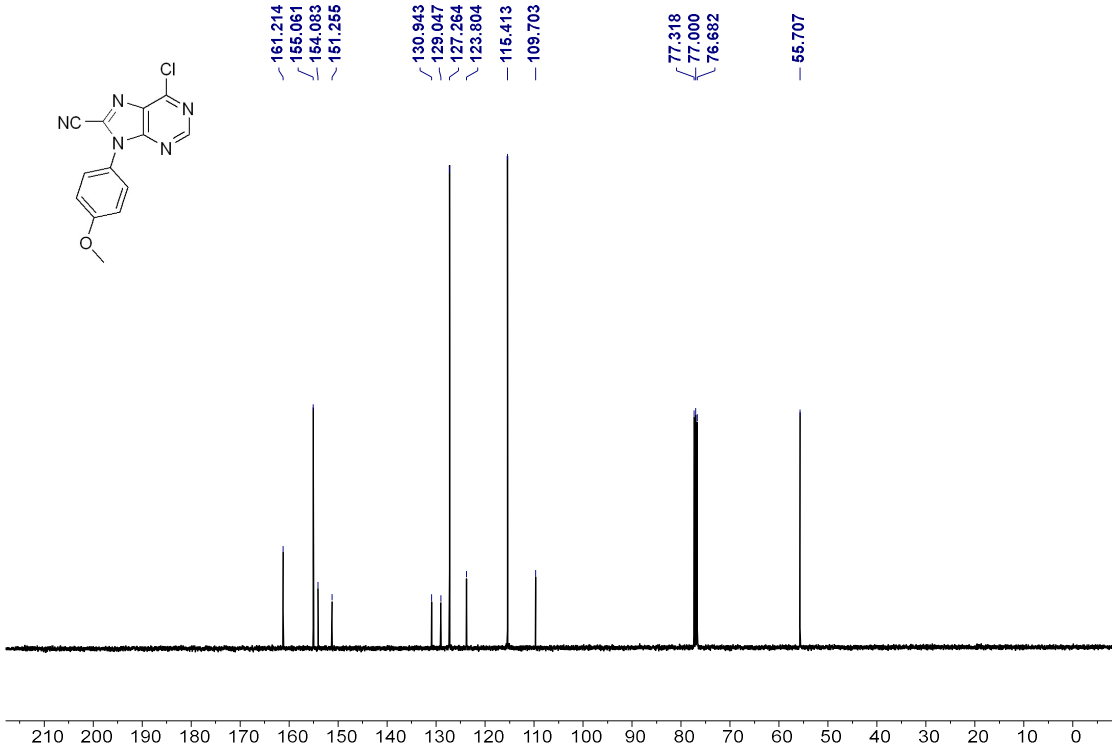

6-chloro-9-(4-(methylthio) phenyl)-9H-purine-8-carbonitrile (**2o**)

^{1}H NMR (400 MHz, $CDCl_3$)

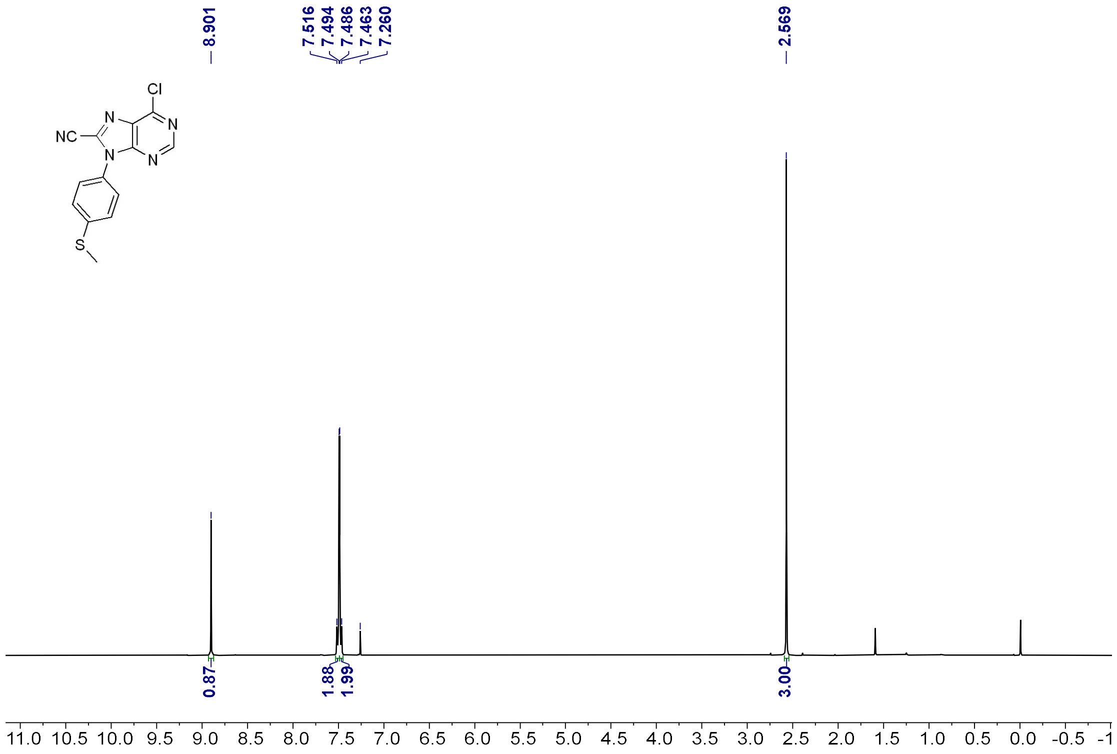

^{13}C NMR (101 MHz, $CDCl_3$)

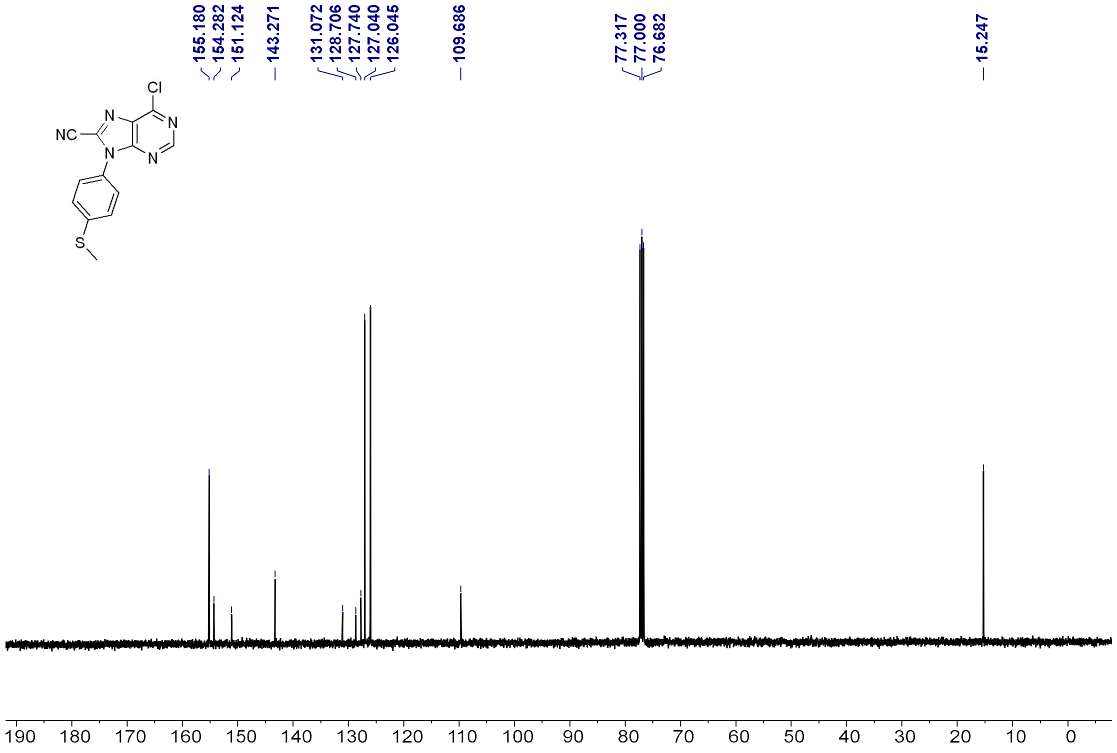

9-(4-bromophenyl)-6-chloro-9H-purine-8-carbonitrile (**2p**)

^{1}H NMR (400 MHz, $CDCl_3$)

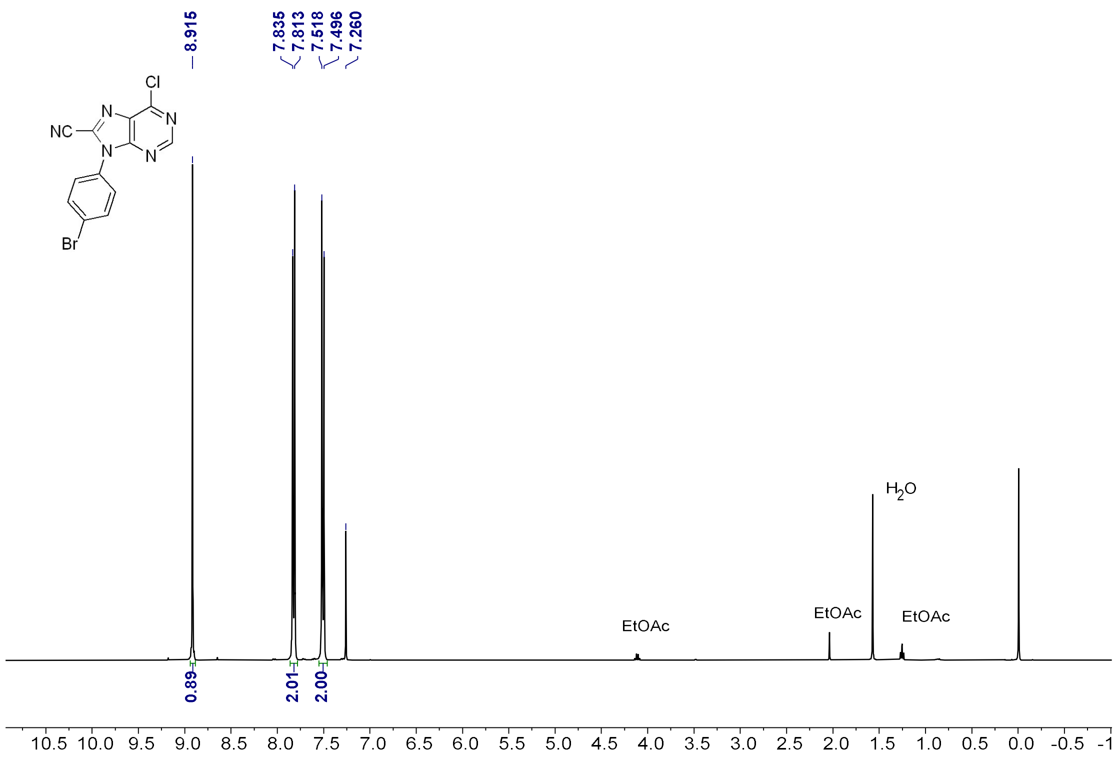

^{13}C NMR (101 MHz, $CDCl_3$)

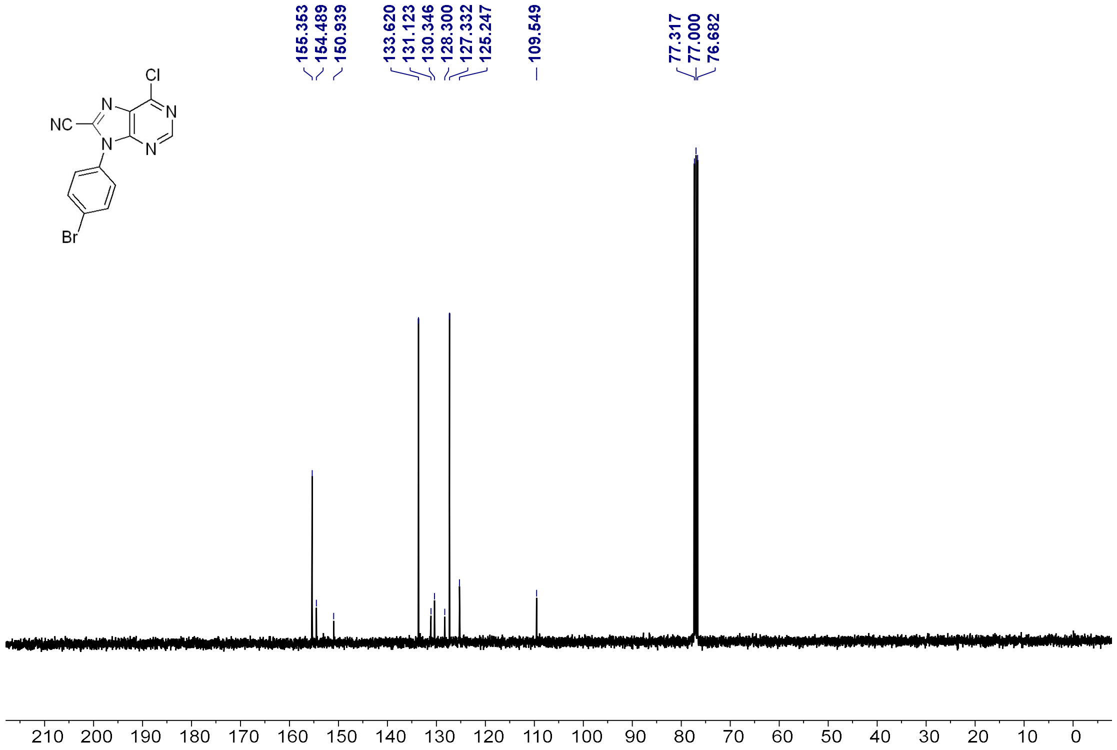

6-chloro-9-(3-chlorophenyl)-9H-purine-8-carbonitrile (**2q**)

^{1}H NMR (400 MHz, $CDCl_3$)

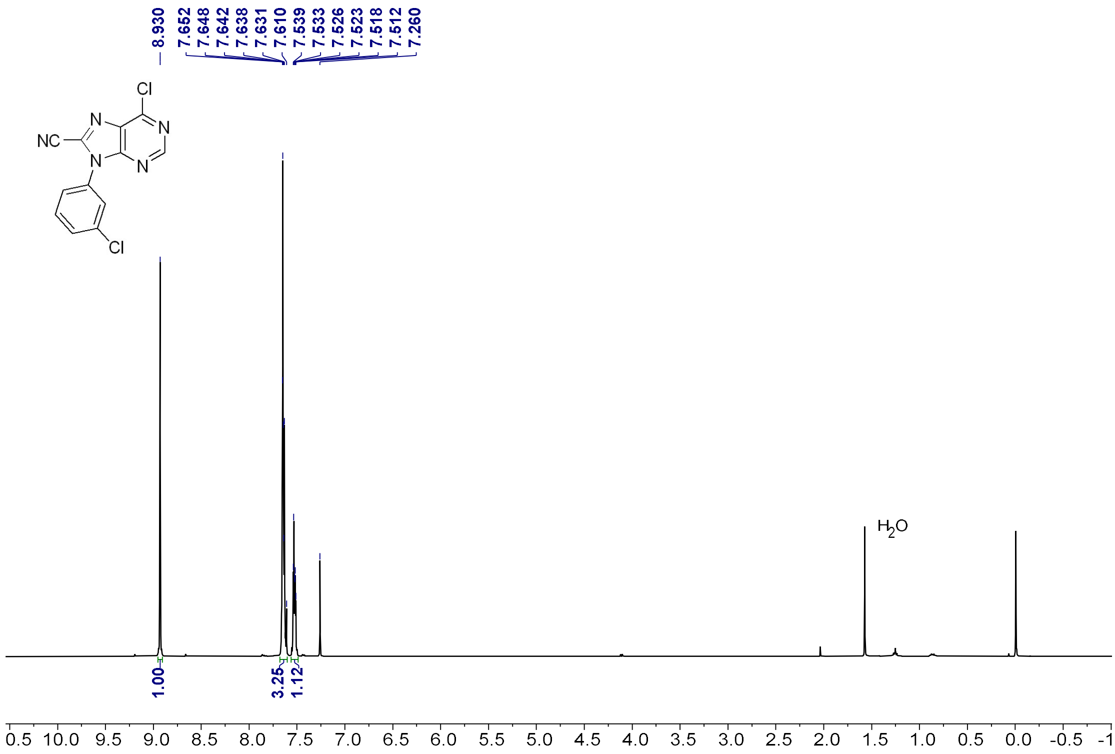

^{13}C NMR (101 MHz, $CDCl_3$)

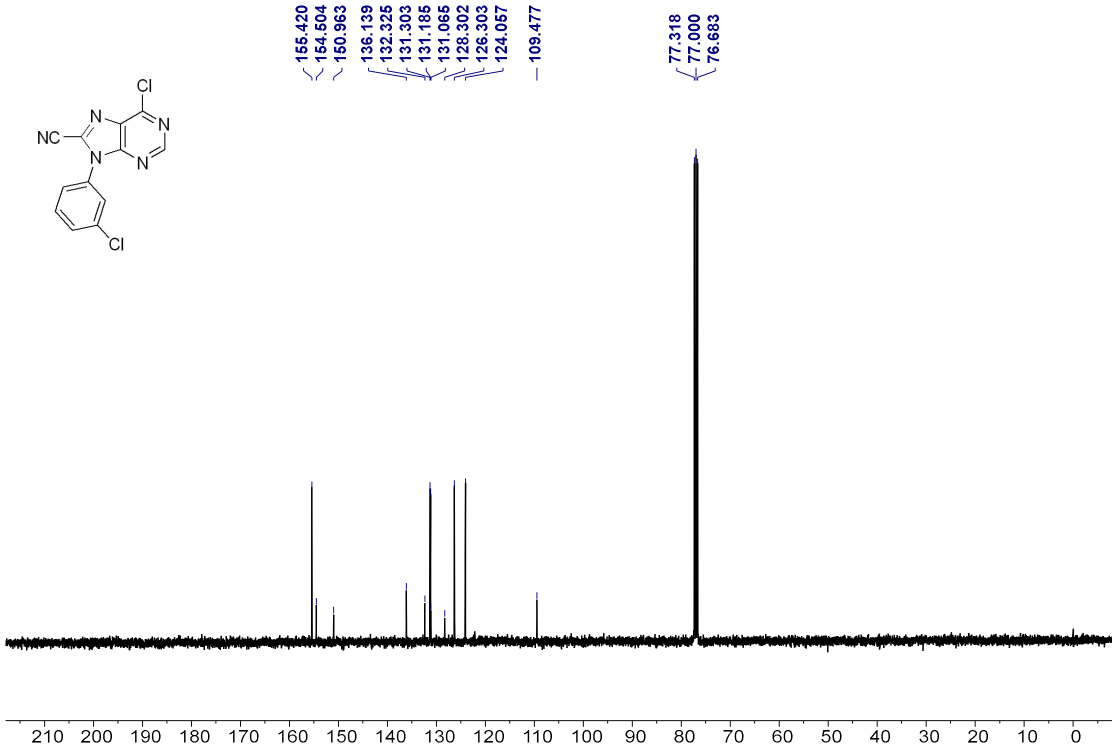

9-benzyl-2,6-dichloro-9H-purine-8-carbonitrile (**2r**)

^{1}H NMR (400 MHz, $CDCl_3$)

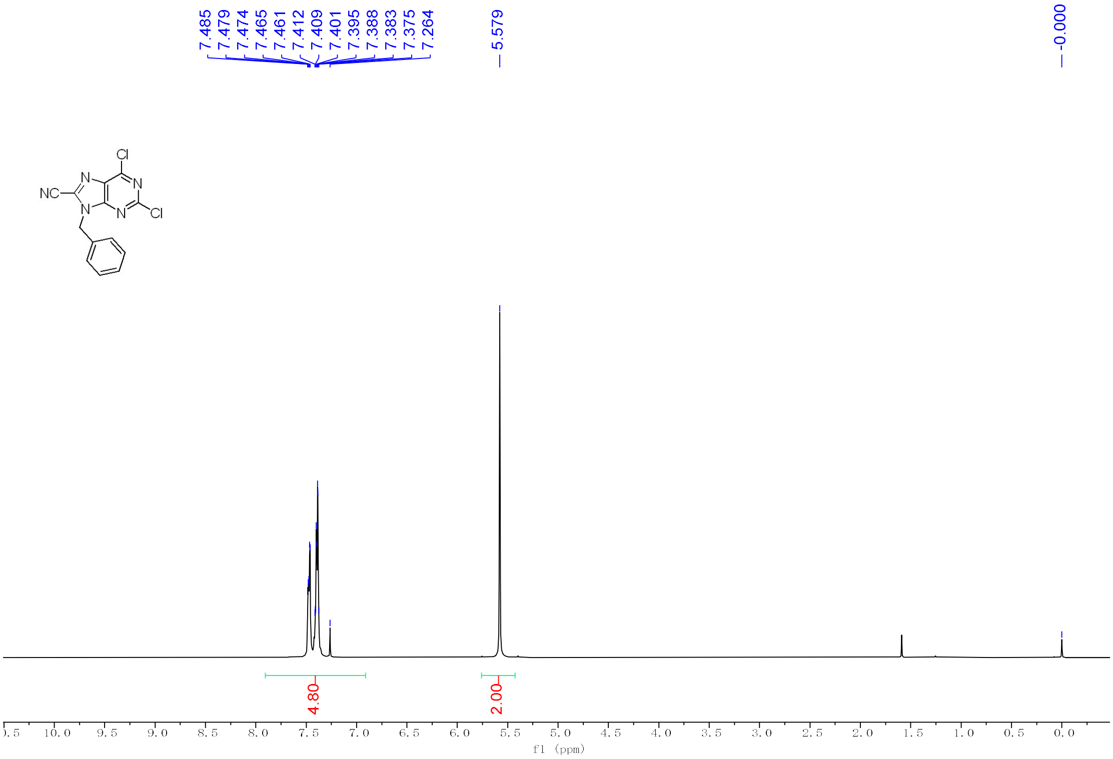

^{13}C NMR (101 MHz, $CDCl_3$)

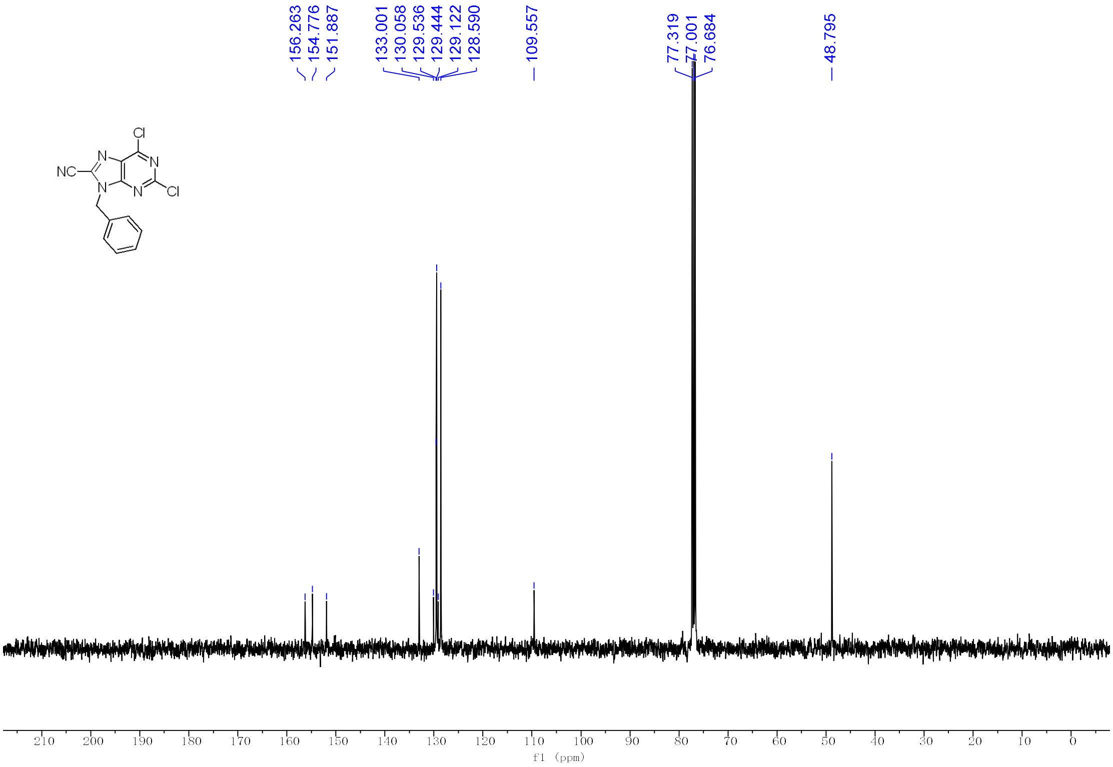

9-benzyl-6-(benzylthio)-9H-purine-8-carbonitrile (**2s**)

^{1}H NMR (400 MHz, CDCl$_3$)

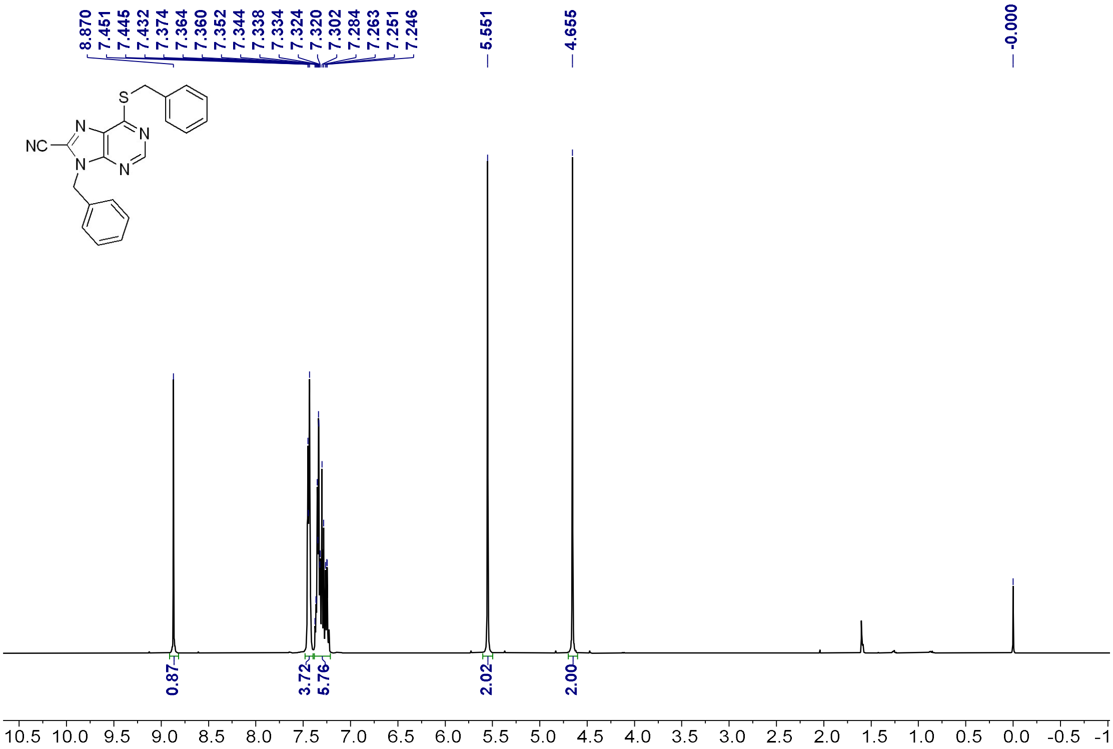

^{13}C NMR (101 MHz, CDCl$_3$)

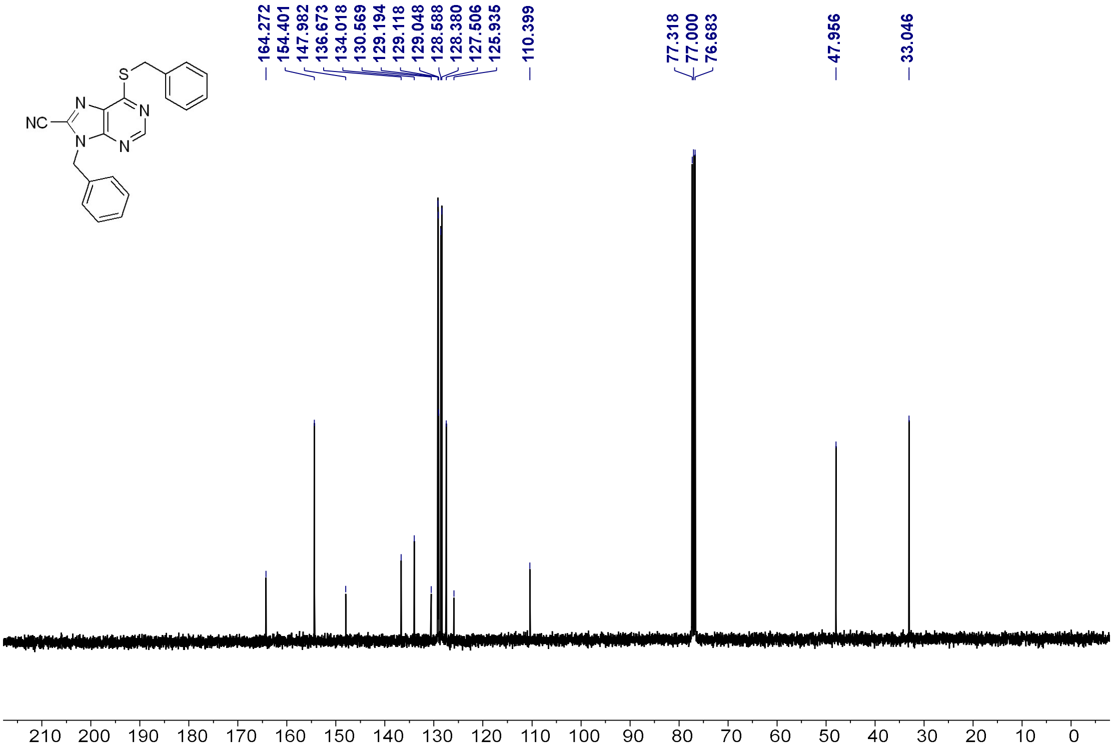

9-benzyl-6-methoxy-9H-purine-8-carbonitrile (**2t**)

^{1}H NMR (400 MHz, $CDCl_3$)

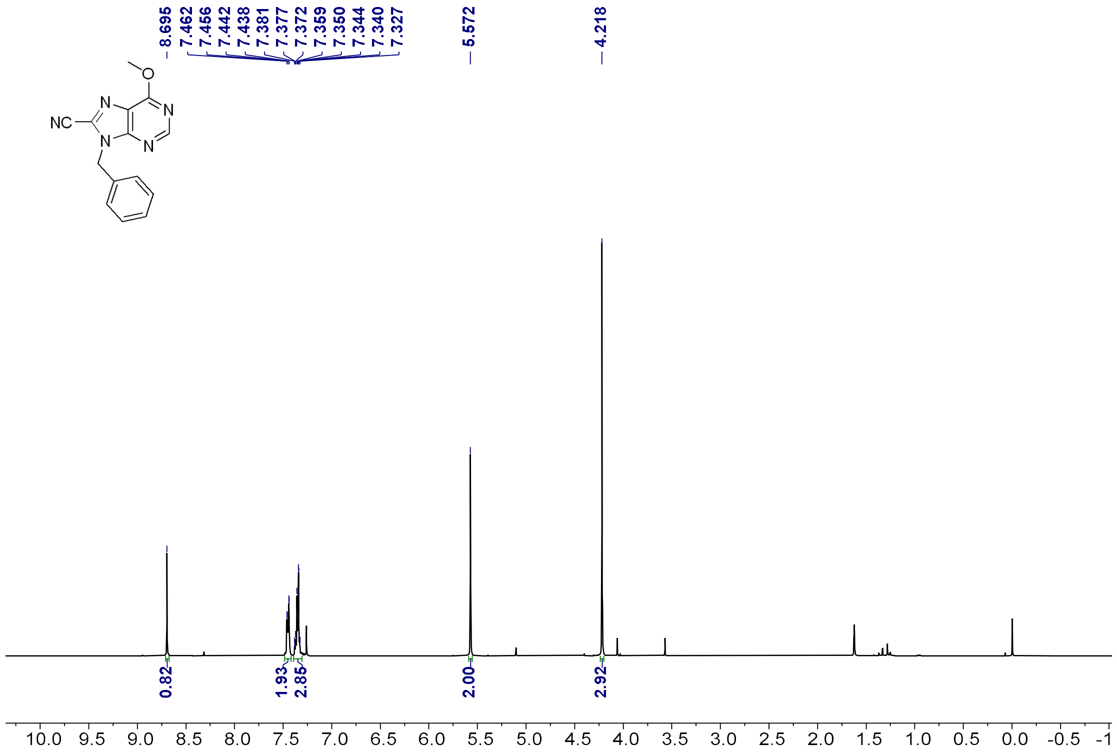

^{13}C NMR (101 MHz, $CDCl_3$)

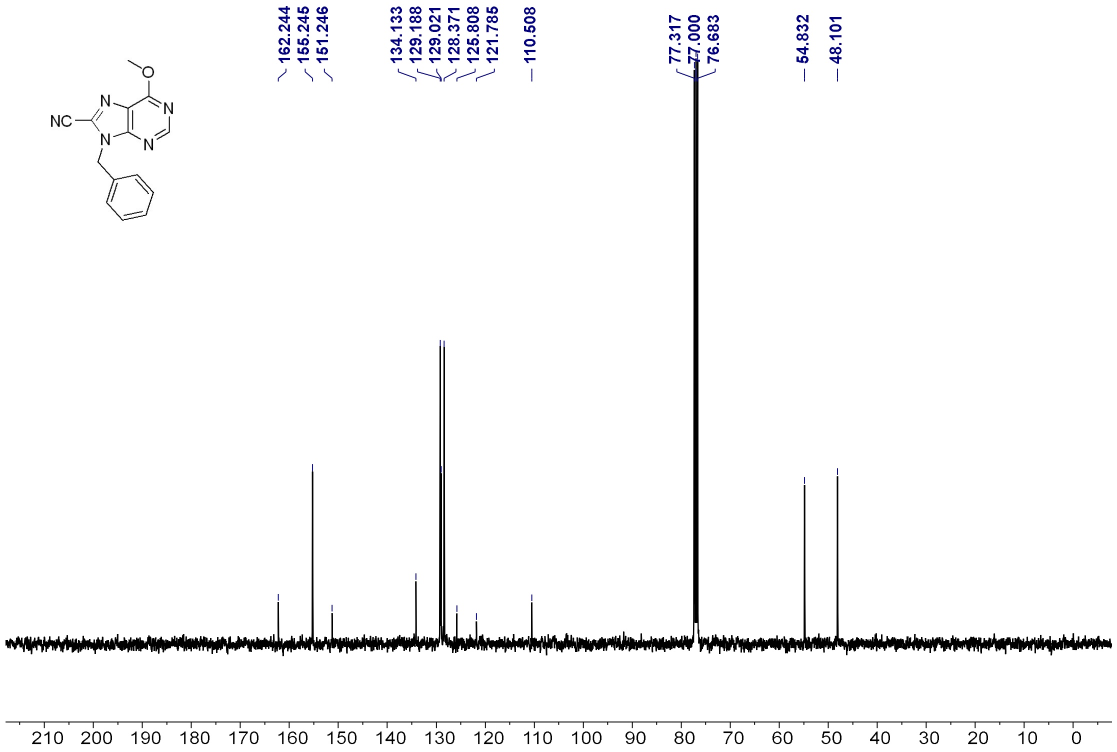

9-Benzyl-6-(p-tolyl)-9H-purine-8-carbonitrile (**2u**)

^{1}H NMR (400 MHz, $CDCl_3$)

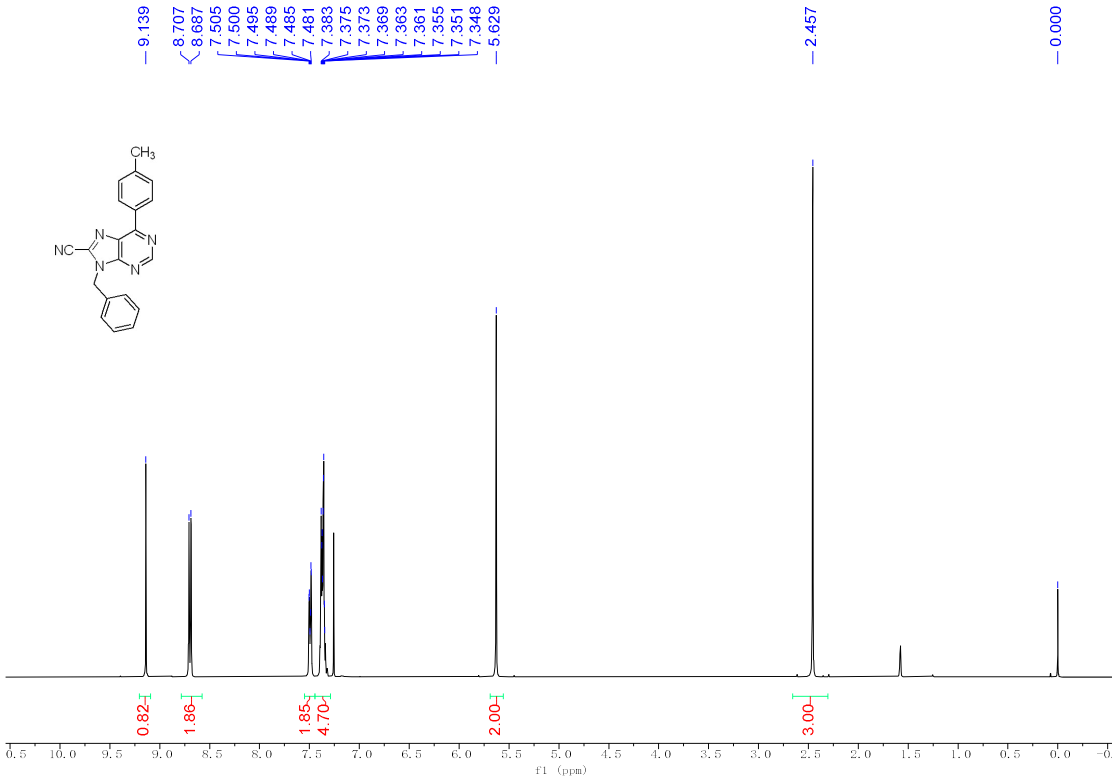

^{13}C NMR (101 MHz, $CDCl_3$)

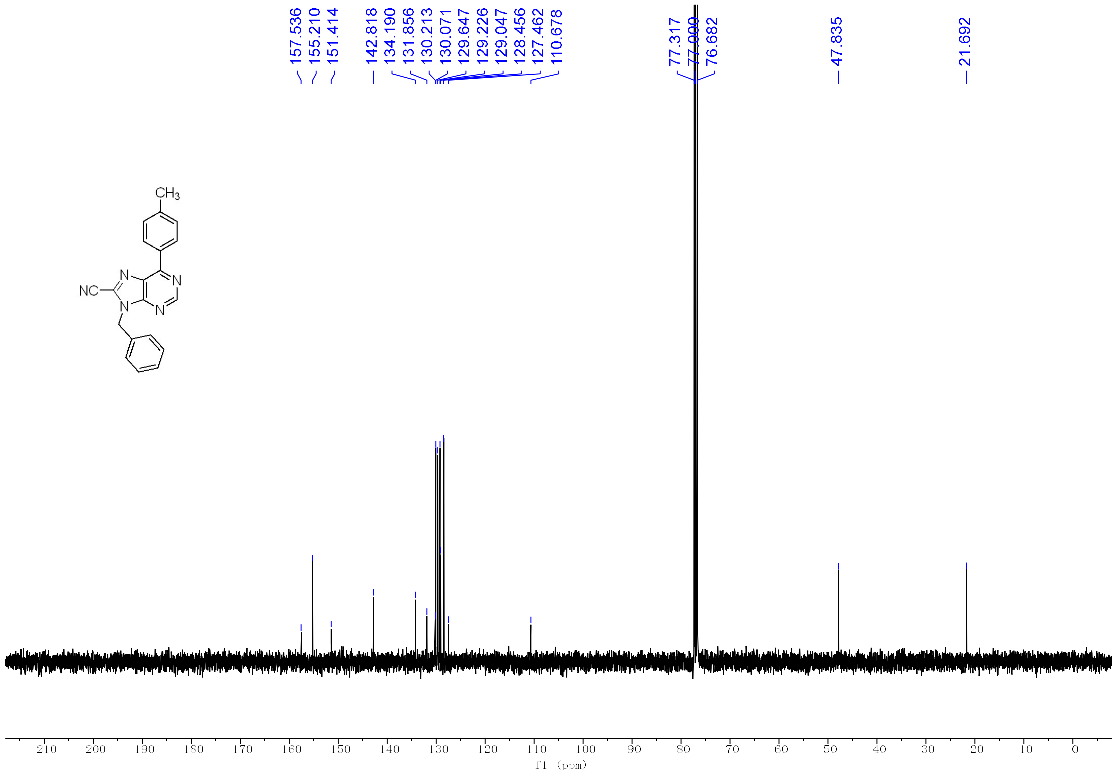

4. The structural identification of 2v and 2v′

9-benzyl-6-(diethylamino)-9H-purine-8-carbonitrile (**2v**)

^{1}H NMR (400 MHz, $CDCl_3$)

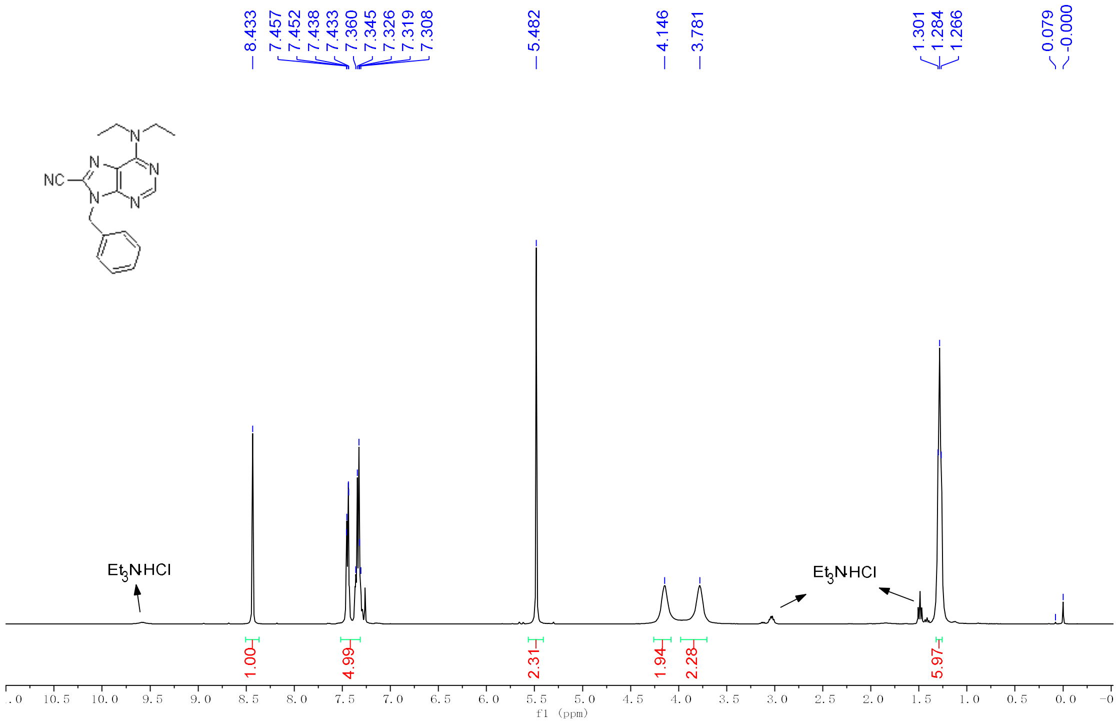

^{13}C NMR (101 MHz, $CDCl_3$)

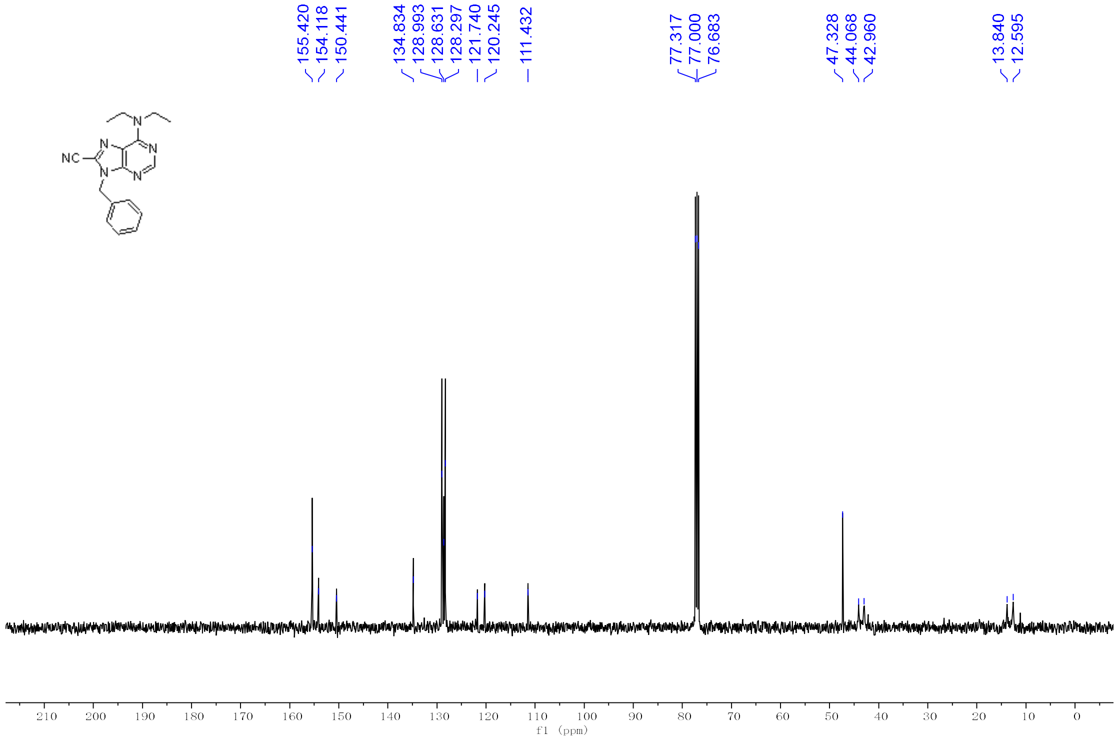

Et3N has been removed by washing with aq HCl and H, and the **2v** was test again in DMSO-d6 where a better carbon NMR data was obtained.

^{1}H NMR (400 MHz, DMSO-*d6*)

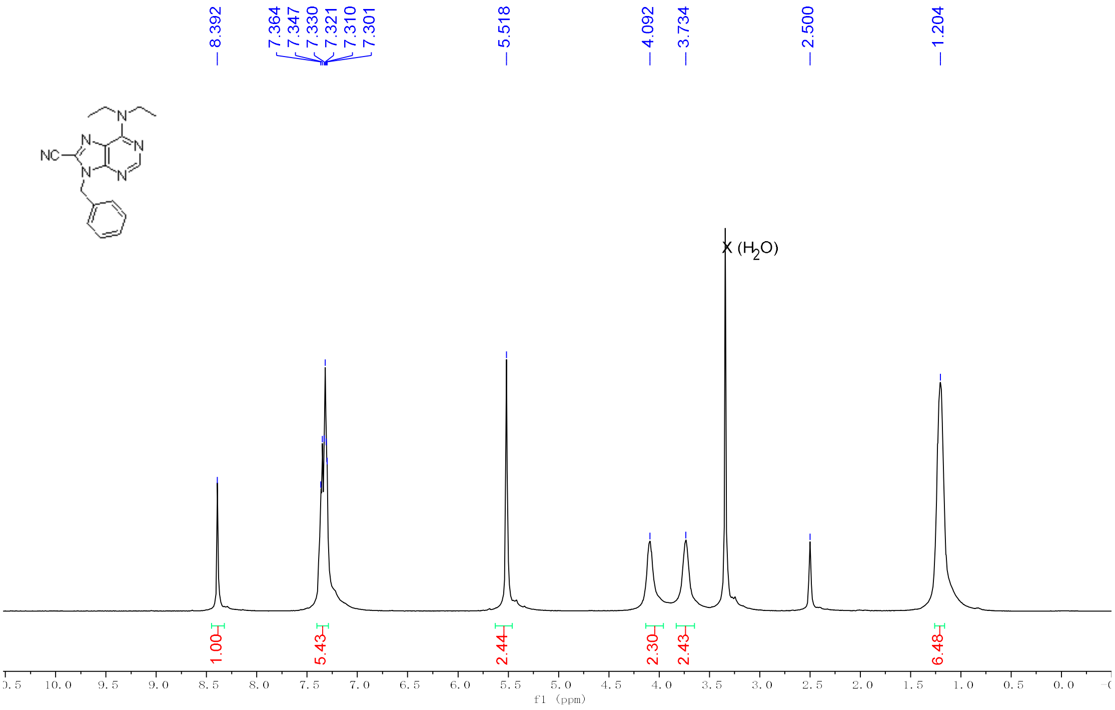

^{13}C NMR (101 MHz, DMSO-*d6*)

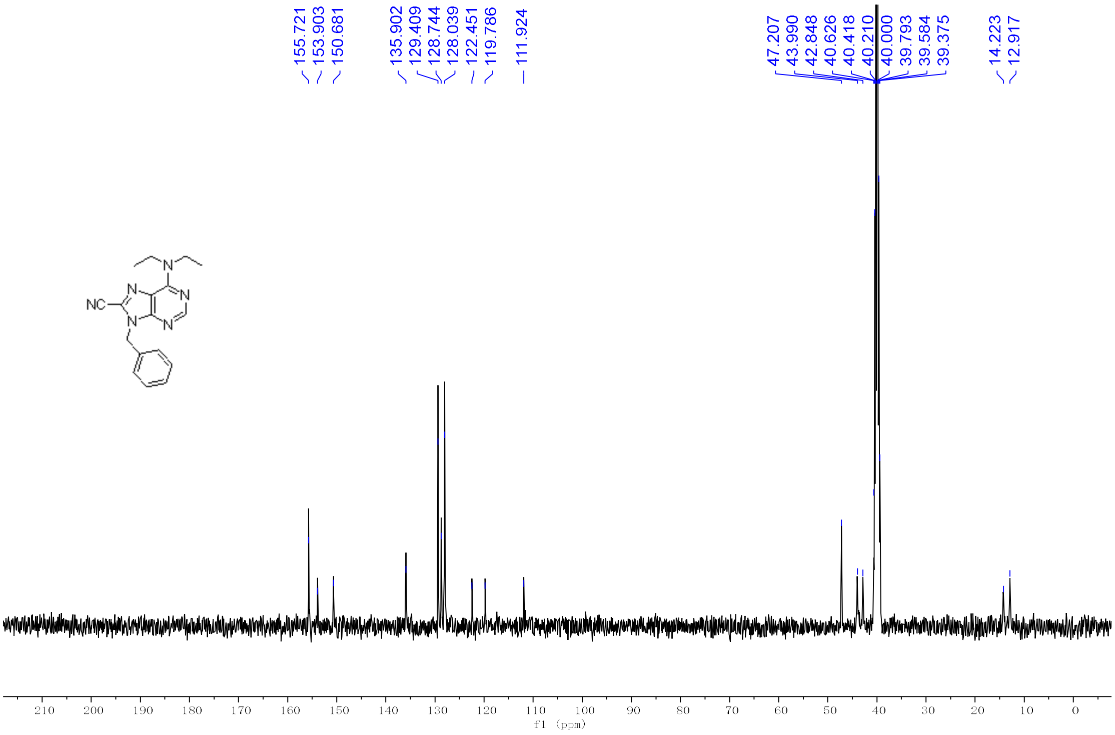

9-benzyl-6-(diethylamino)-9H-purine-2-carbonitrile (**2v′**)

^{1}H NMR (400 MHz, $CDCl_3$)

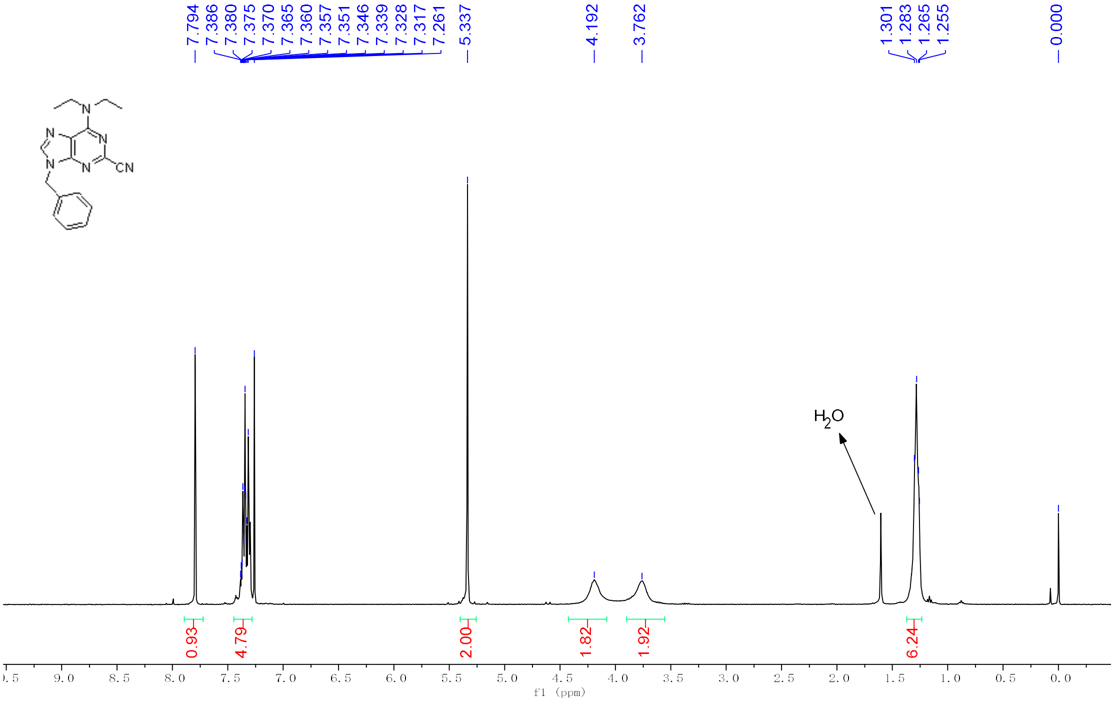

^{13}C NMR (101 MHz, $CDCl_3$)

The ^{13}C NMR spectrum was tested twice; the first one was scanned for 164 times where the alkyl carbons in Et_2N were totally absent and the second one was scanned for 1250 times (~ 90 min) where the alkyl carbons in Et_2N were appeared, but they were still broad and weak apparently..

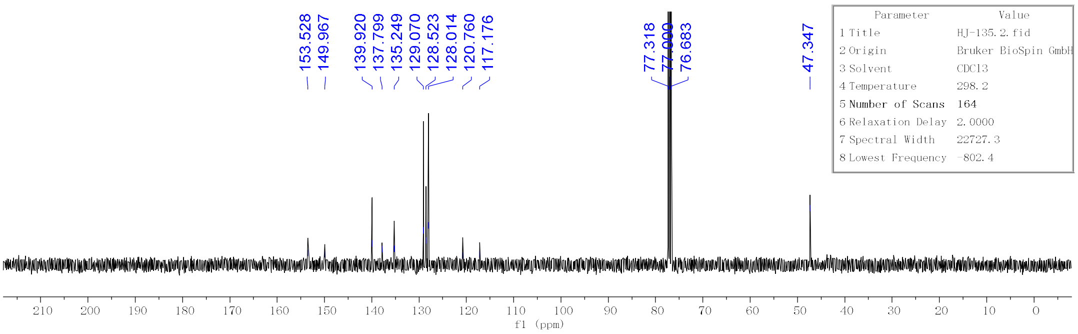

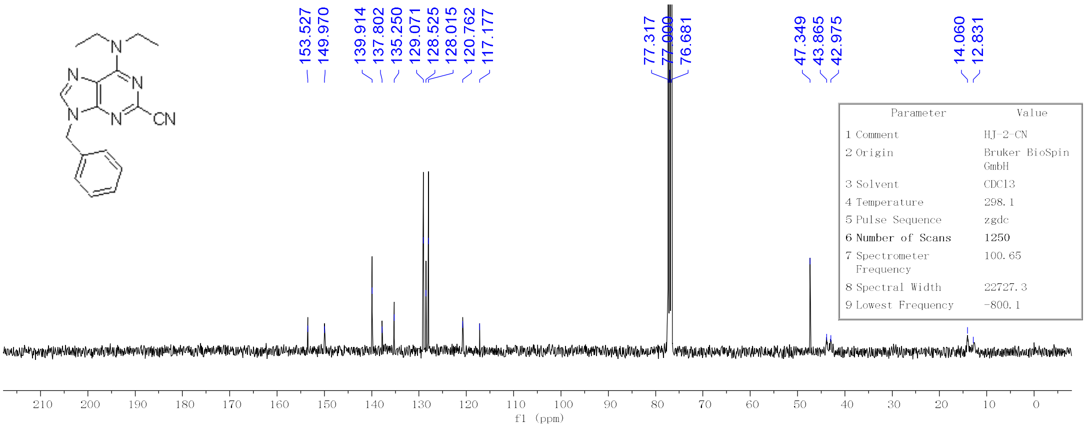

The HSQC of **2v′** was thus performed (NS = 2, 1TD = 128, ~ 10 min), where the cross peak of ^1J coupling between hydrogen and the weak carbons were obvious emerged. The HSQC spectrum highly supports the structure of **2v′**.

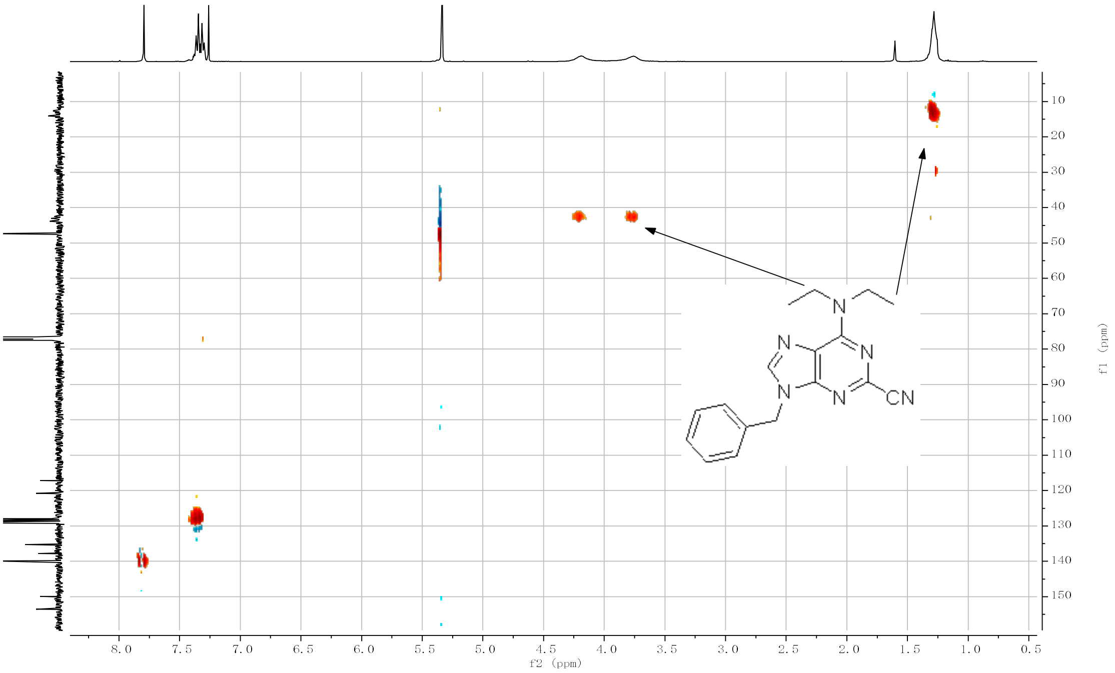

5. Copies of ^{1}H and ^{13}C-NMR spectra of 3r, 4u, 5f, 5s & 6f

9-benzyl-2,6-dichloro-7-((trifluoromethyl)sulfonyl)-8,9-dihydro-7H-purine-8-carbonitrile (**3r**)

^{1}H NMR (400 MHz, $CDCl_3$)

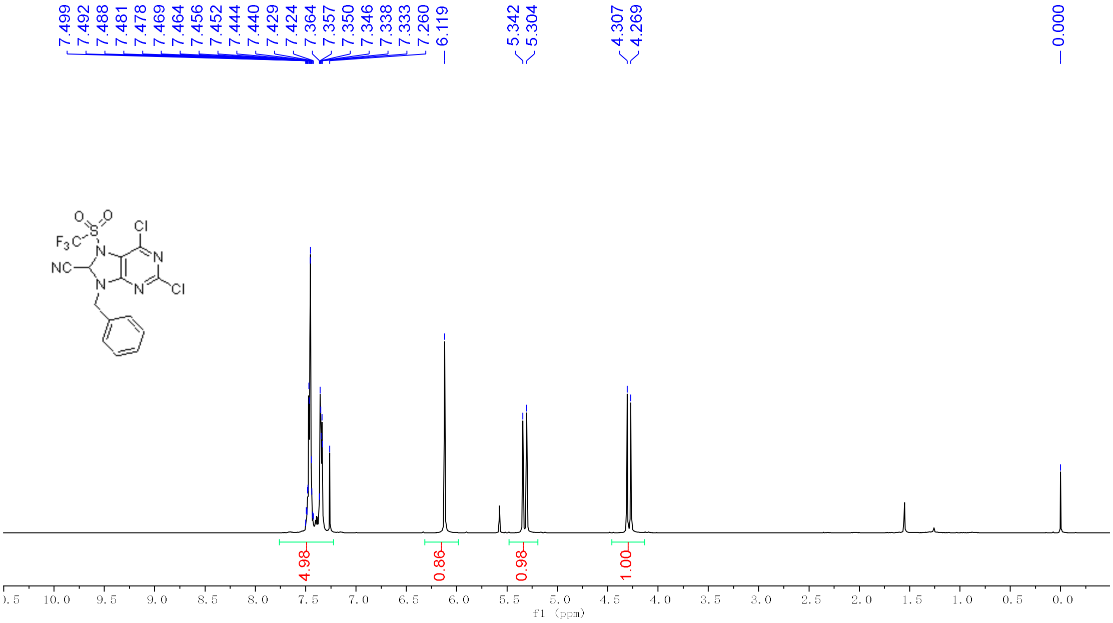

^{13}C NMR (101 MHz, $CDCl_3$)

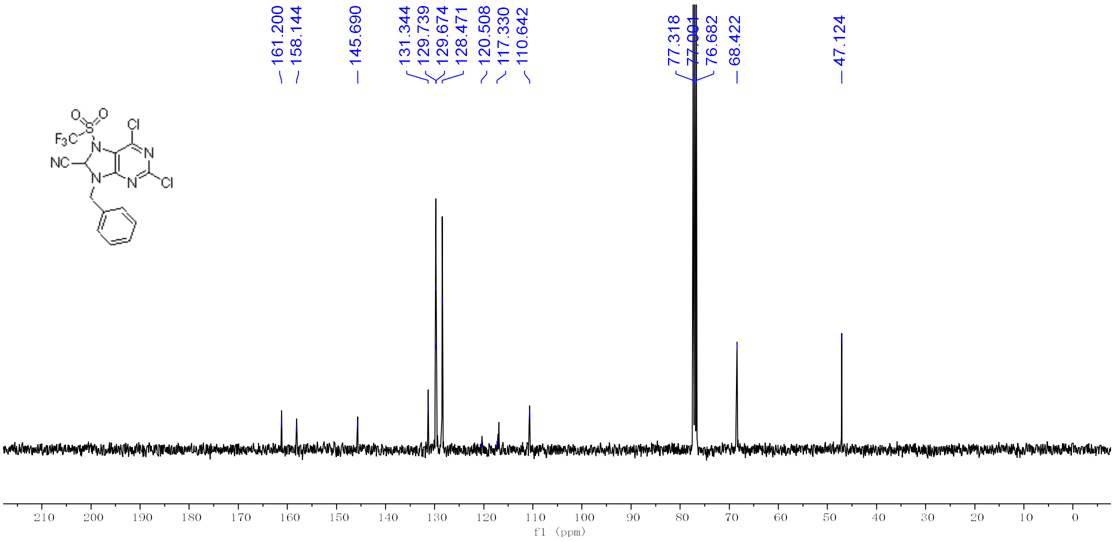

^{19}F NMR (376 MHz, $CDCl_3$)

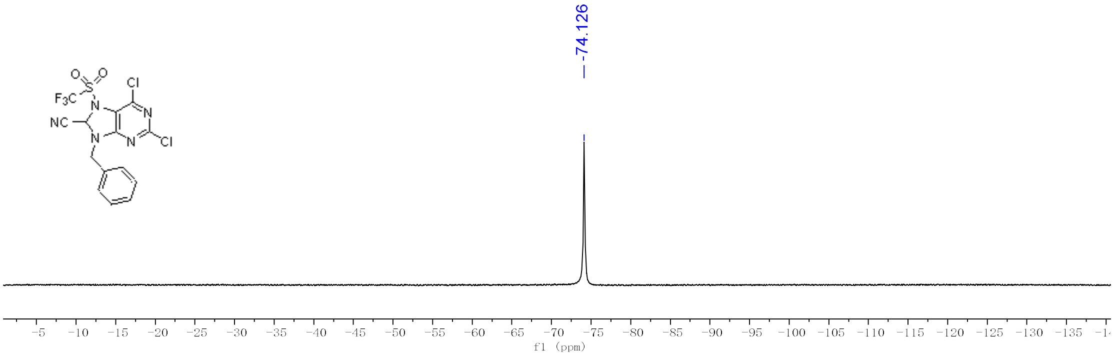

9-benzyl-6-(p-tolyl)-9H-purine-2,8-dicarbonitrile (**4u**)

^{1}H NMR (400 MHz, $CDCl_3$)

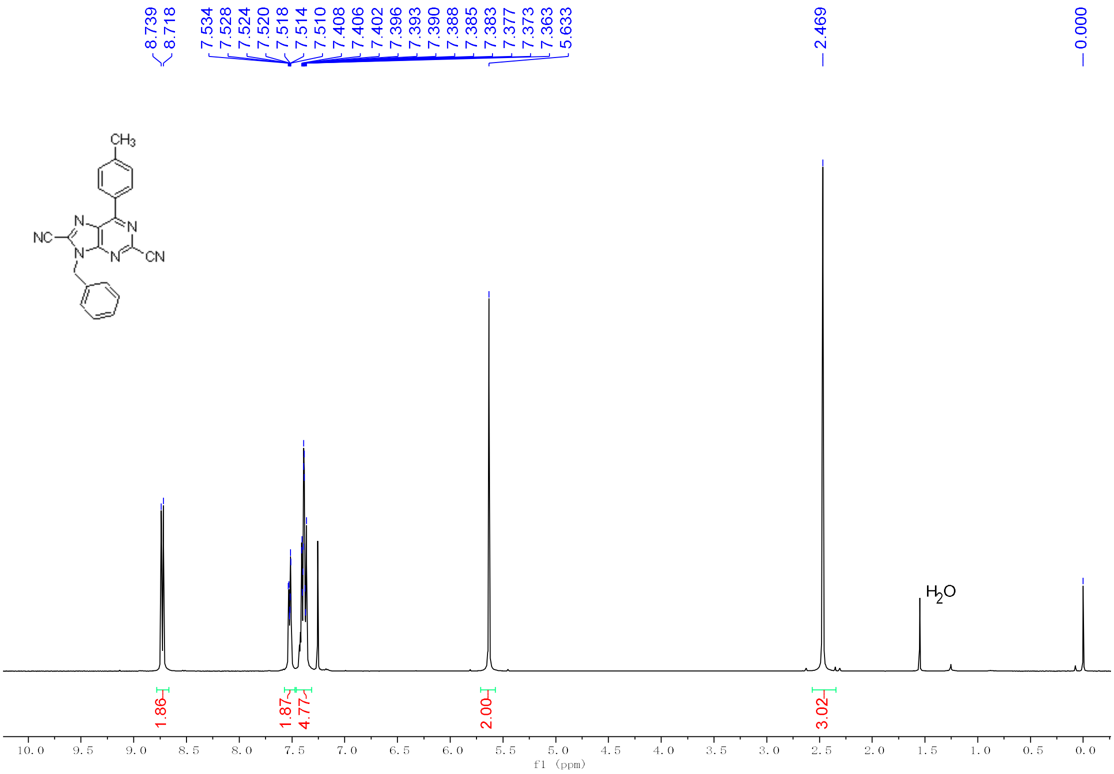

^{13}C NMR (101 MHz, $CDCl_3$)

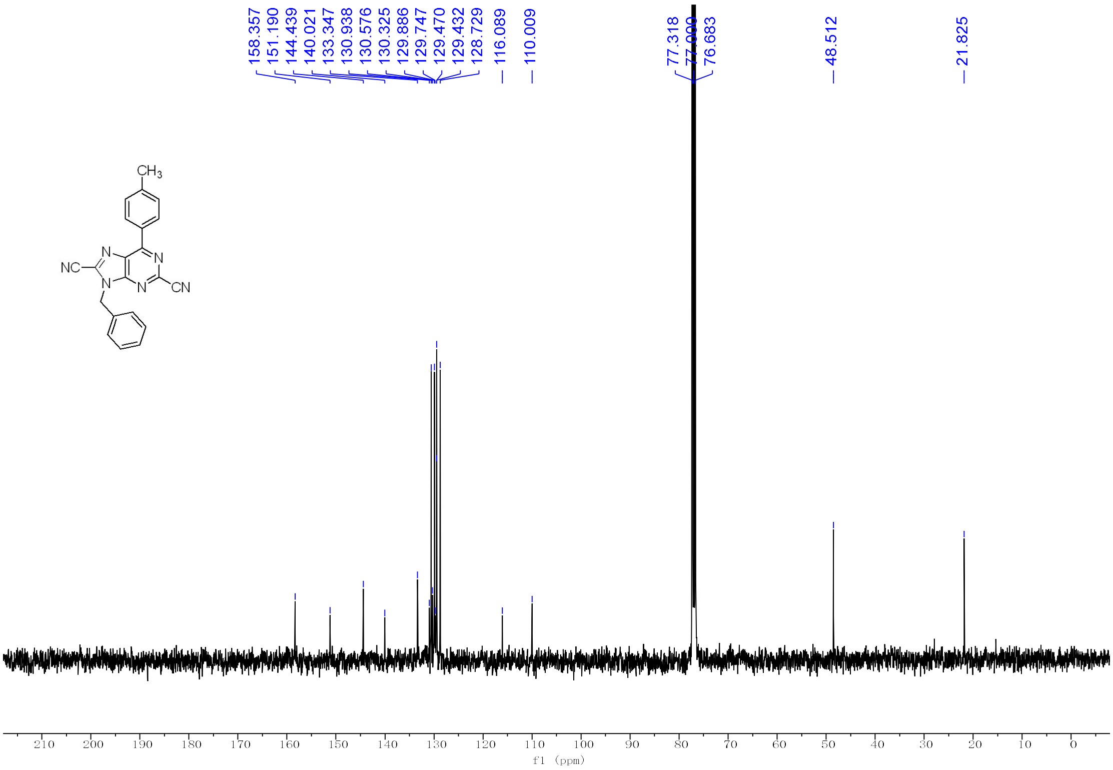

9-benzyl-6-(diethylamino)-N,N-diethyl-9H-purine-8-carboximidamide (**5f**)

^{1}H NMR (400 MHz, $CDCl_3$)

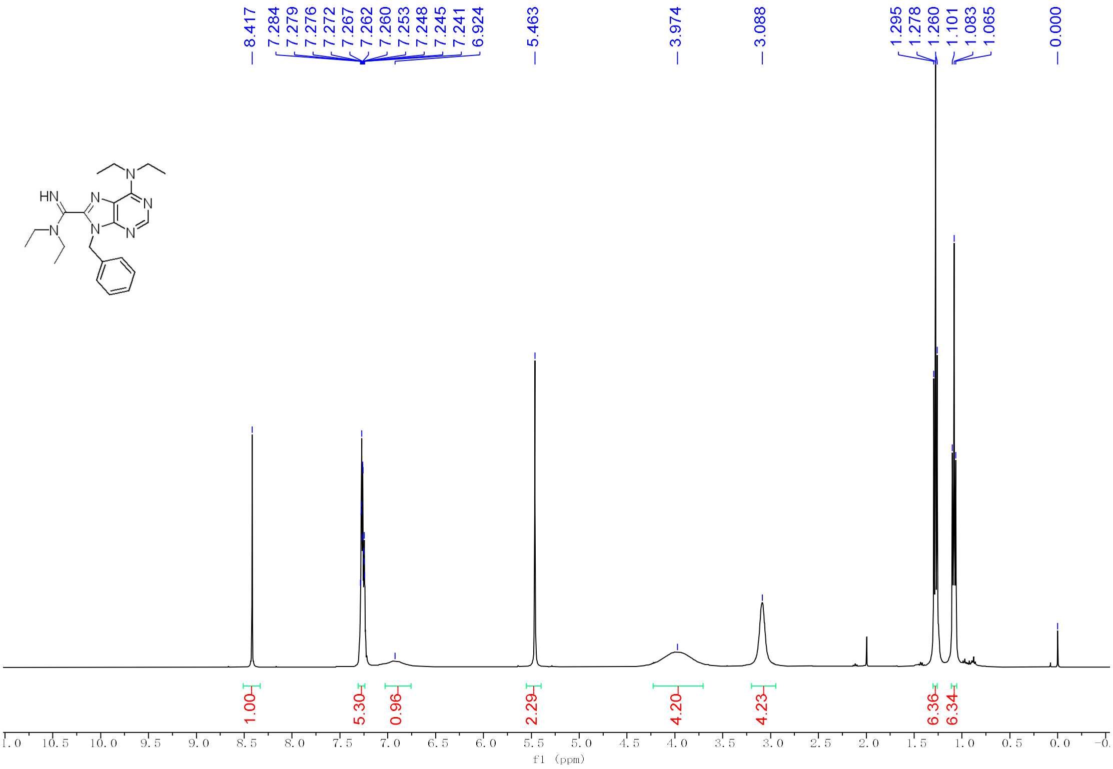

^{13}C NMR (101 MHz, $CDCl_3$)

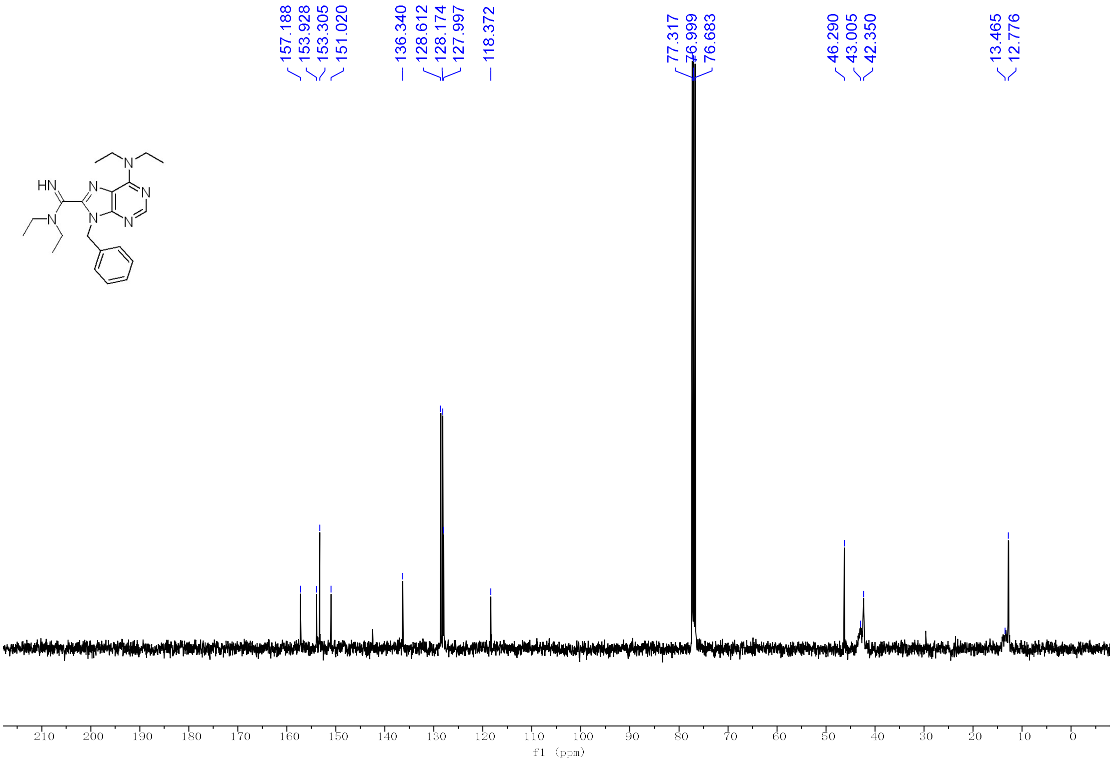

9-benzyl-6-((4-methoxyphenyl)ethynyl)-9H-purine-8-carbonitrile (**6f**)

^{1}H NMR (400 MHz, $CDCl_3$)

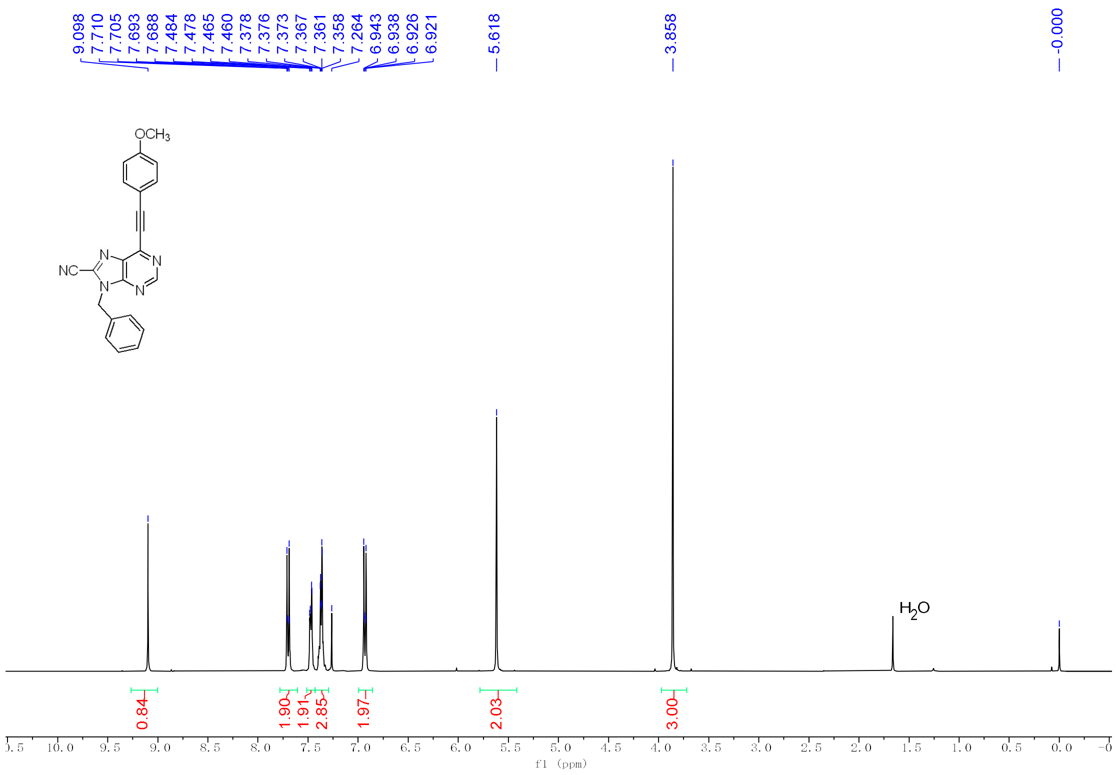

^{13}C NMR (101 MHz, $CDCl_3$)

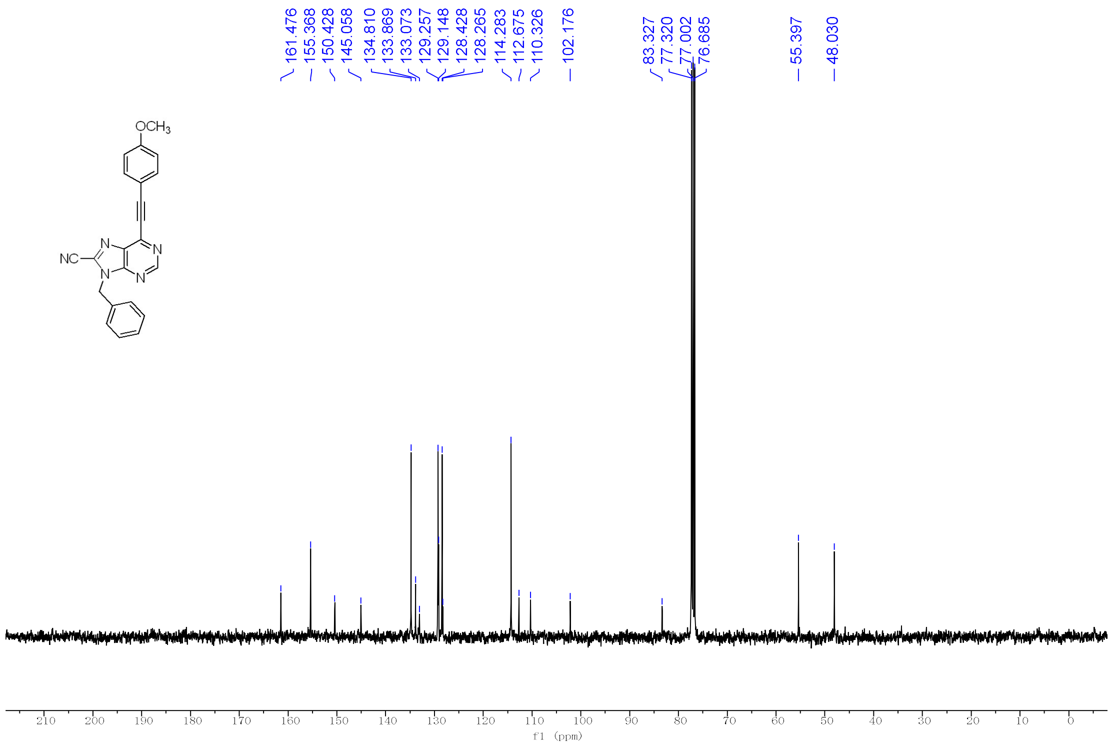

9-benzyl-6,8-bis(benzylthio)-9H-purine (**5s**)

^{1}H NMR (400 MHz, $CDCl_3$)

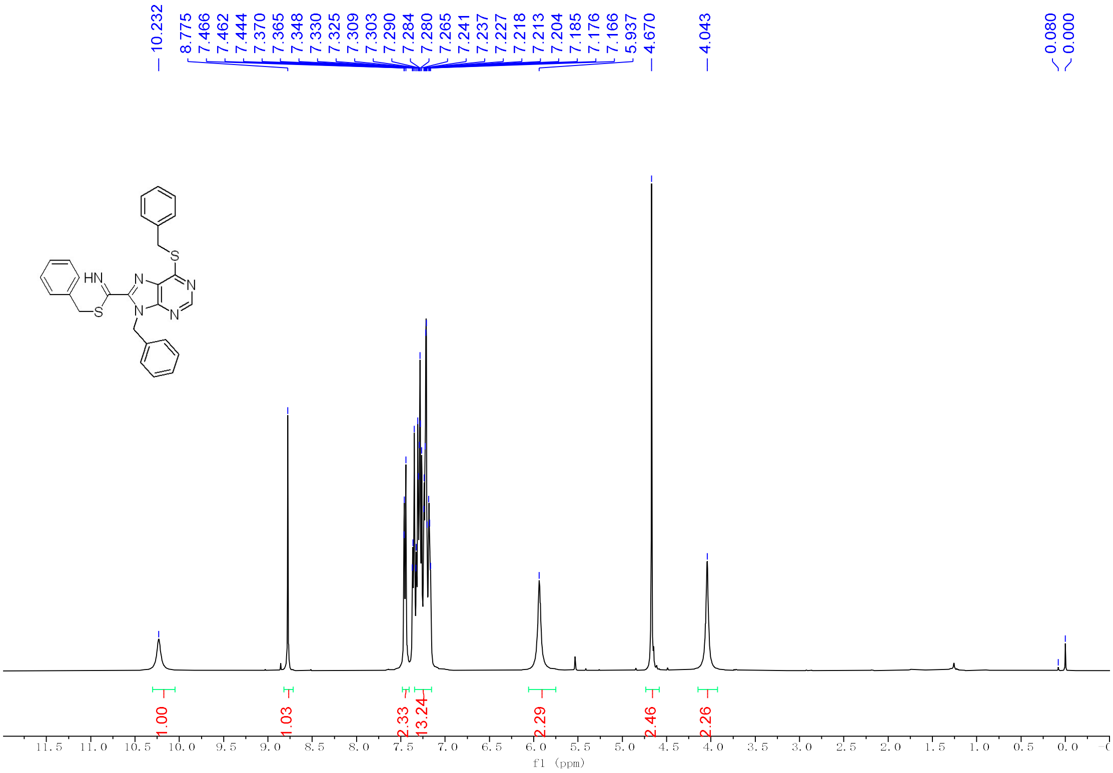

^{13}C NMR (101 MHz, $CDCl_3$)

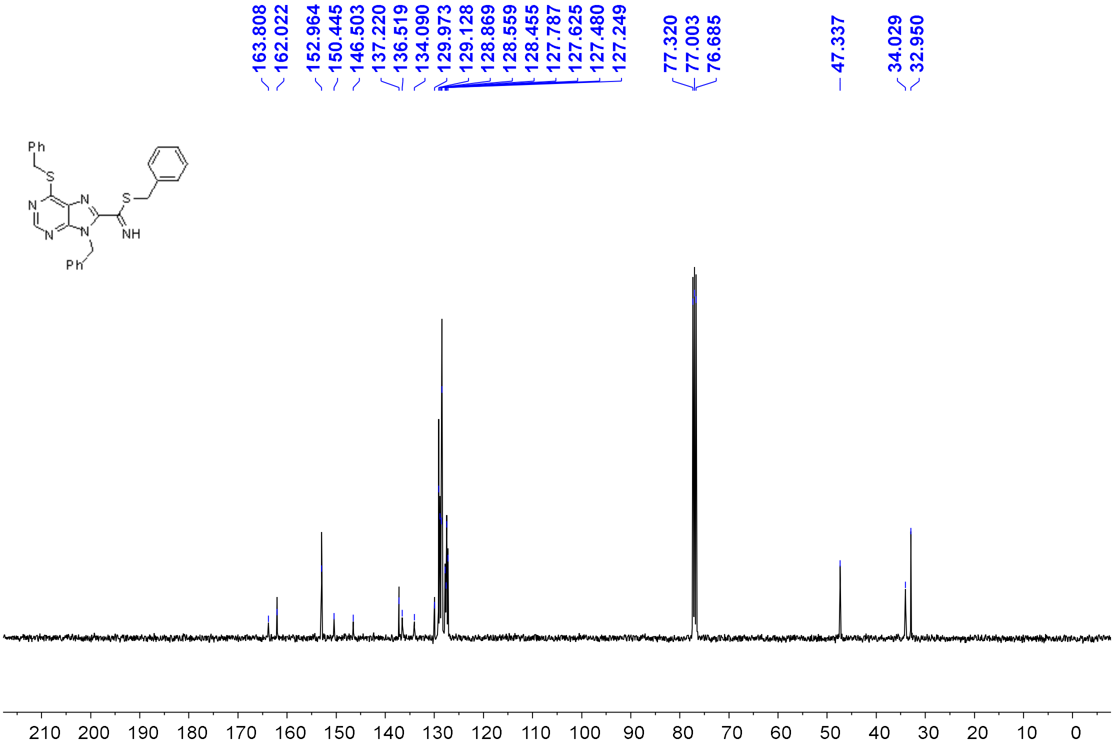

6. Copies of ¹H and ¹³C-NMR spectra of 6s, 6s', 7s, 8s &10s.

9-benzyl-6-(benzylthio)-*9H*-purine-8-carbimidate (**6s**)

¹H NMR (400 MHz, CDCl₃)

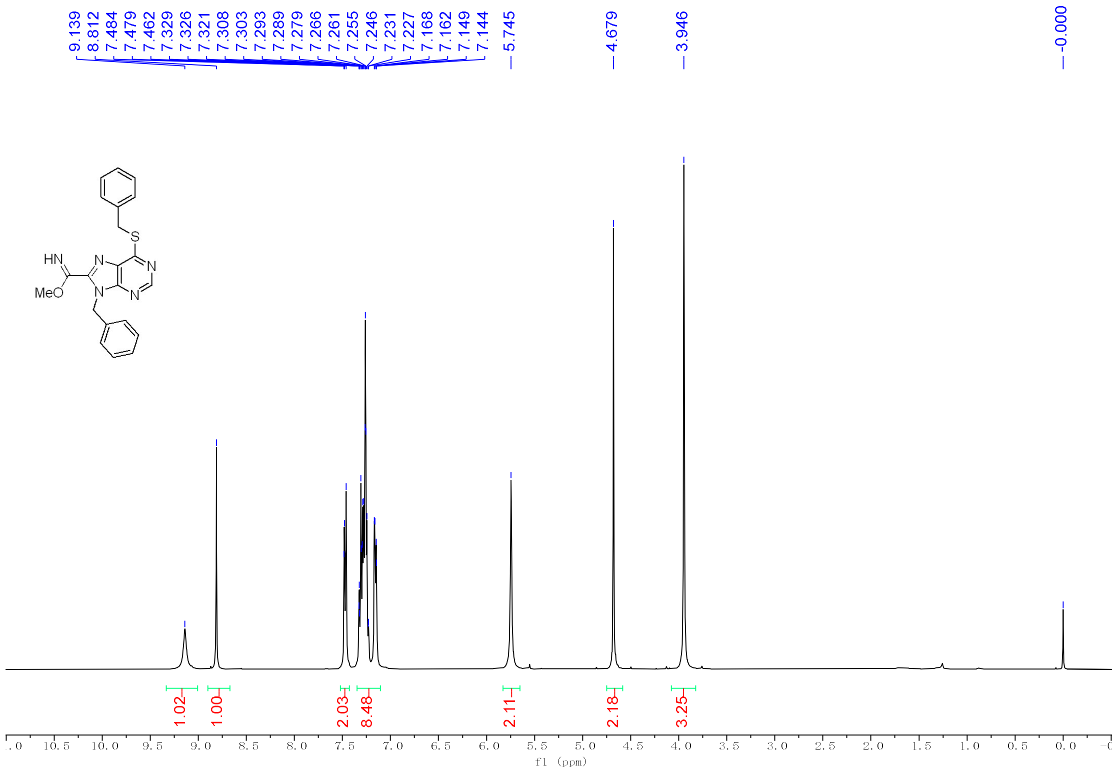

¹³C NMR (101 MHz, CDCl₃)

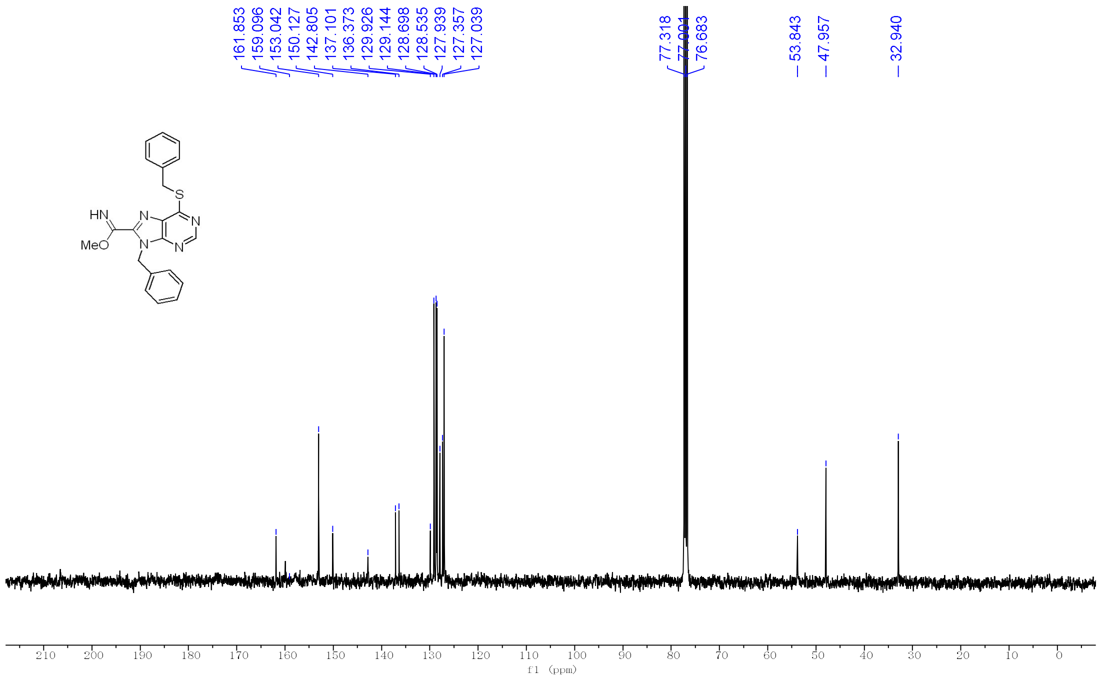

9-benzyl-6-(benzylthio)-9H-purine-8-carbimidate (**6s′**)

^{1}H NMR (400 MHz, $CDCl_3$)

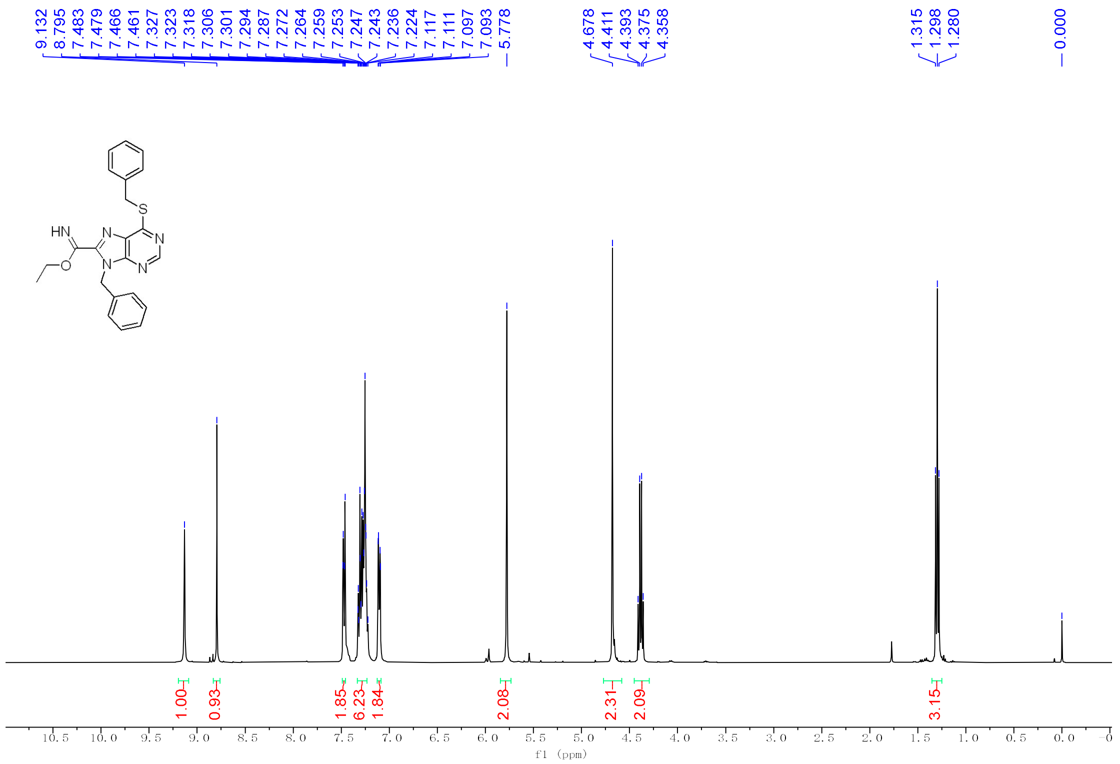

^{13}C NMR (101 MHz, $CDCl_3$)

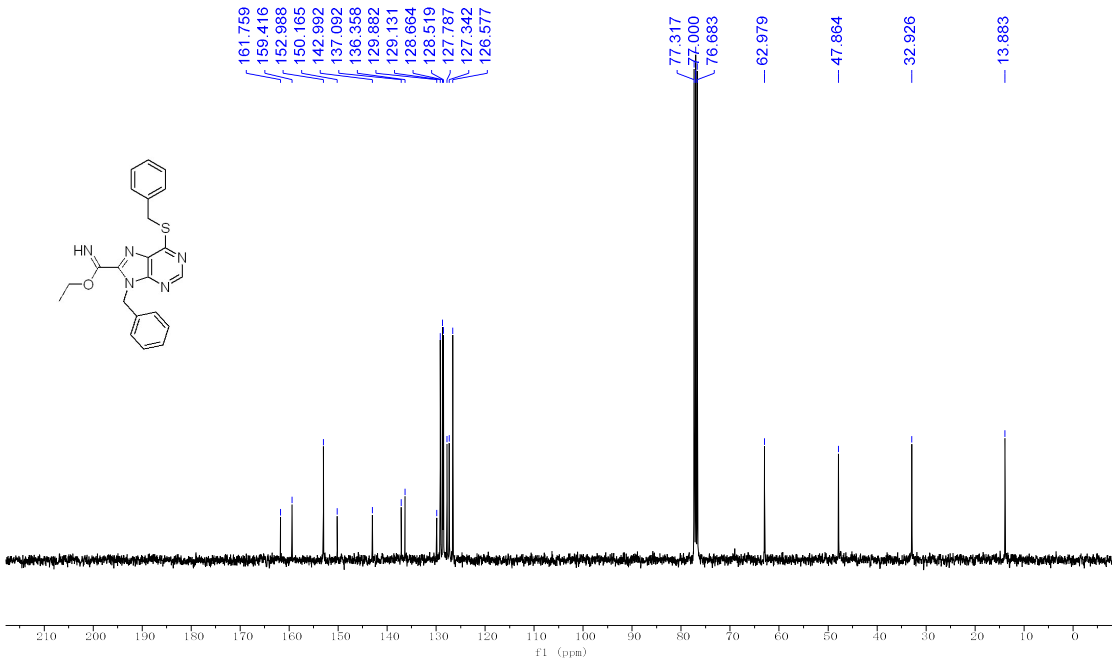

2-(9-benzyl-6-(benzylthio)-9*H*-purin-8-yl)-4,4-dimethyl-4,5-dihydrooxazole (**7s**)

^{1}H NMR (400 MHz, CDCl$_3$)

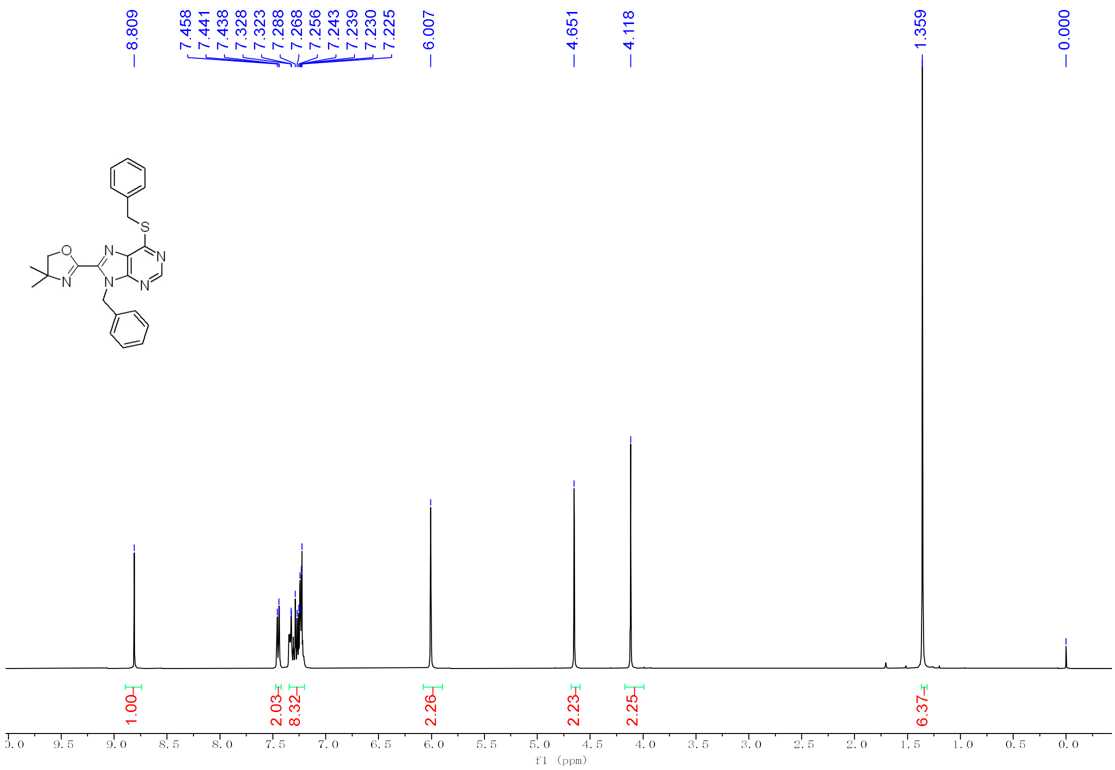

^{13}C NMR (101 MHz, CDCl$_3$)

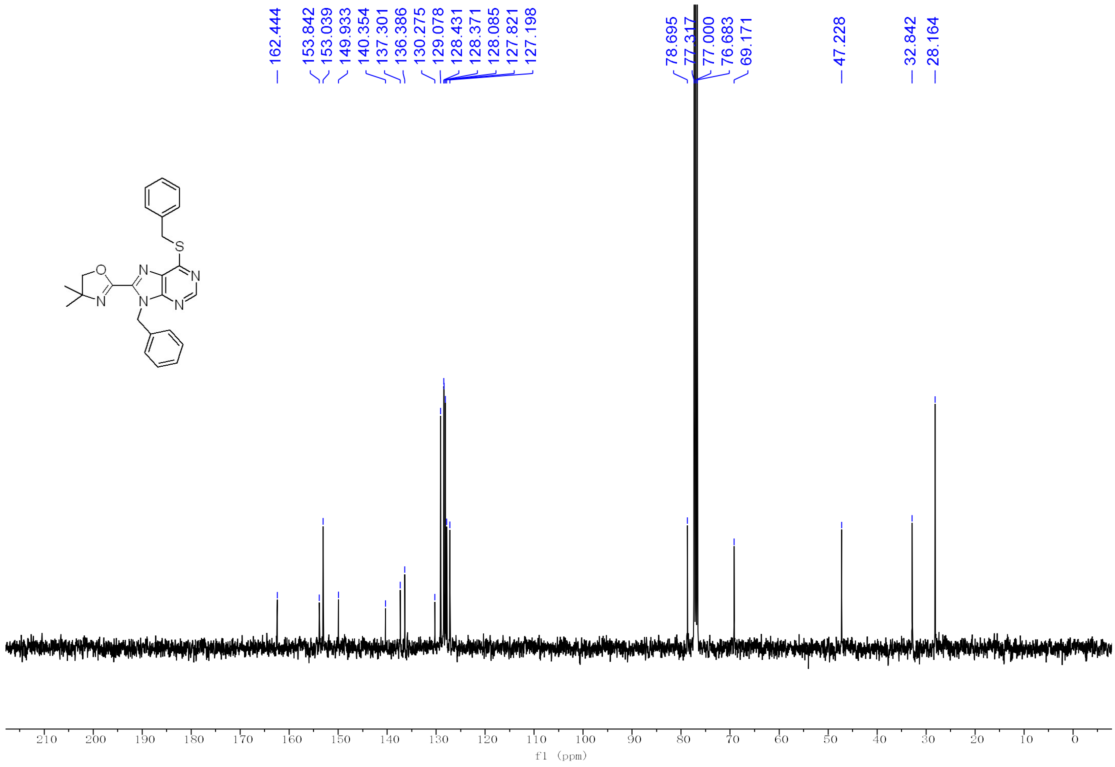

9-benzyl-6-(benzylthio)-*9H*-purine-8-carboxamide (**8s**)

^{1}H NMR (400 MHz, DMSO-*d*$_6$)

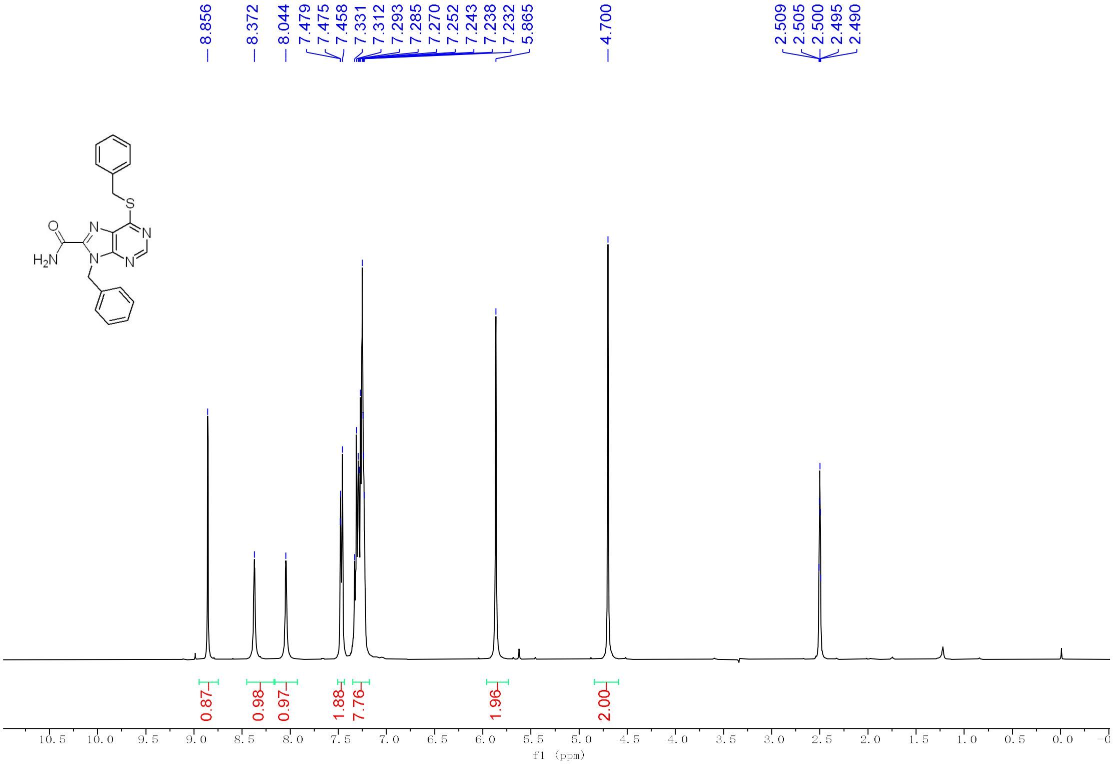

^{13}C NMR (101 MHz, DMSO-*d*$_6$)

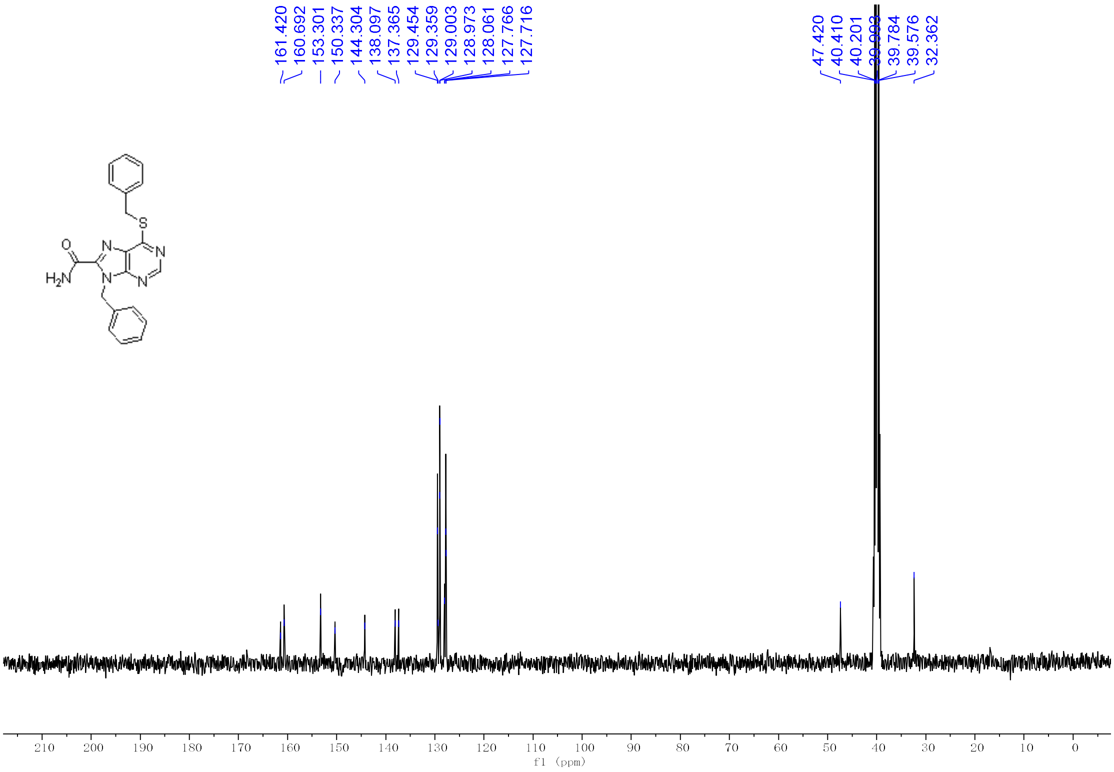

3-(9-benzyl-6-(benzylthio)-*9H*-purin-8-yl)isothiazole-4,5-dicarboxylate (**10s**)

^{1}H NMR (400 MHz, $CDCl_3$)

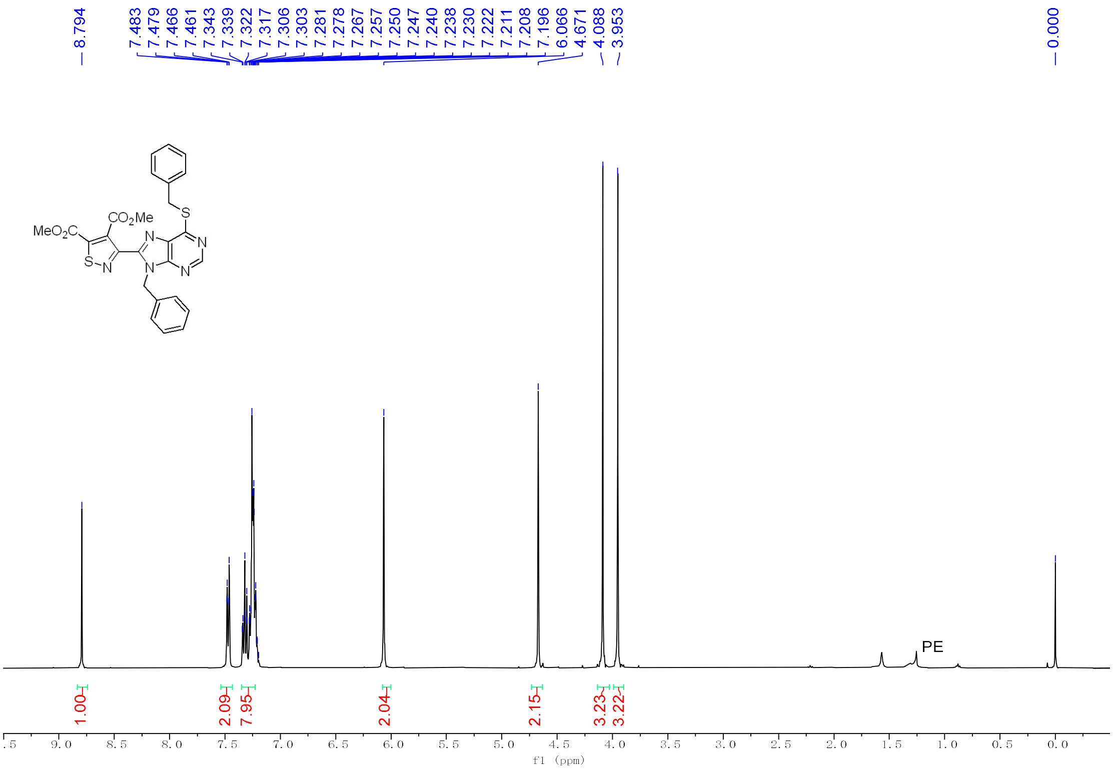

^{13}C NMR (101 MHz, $CDCl_3$)

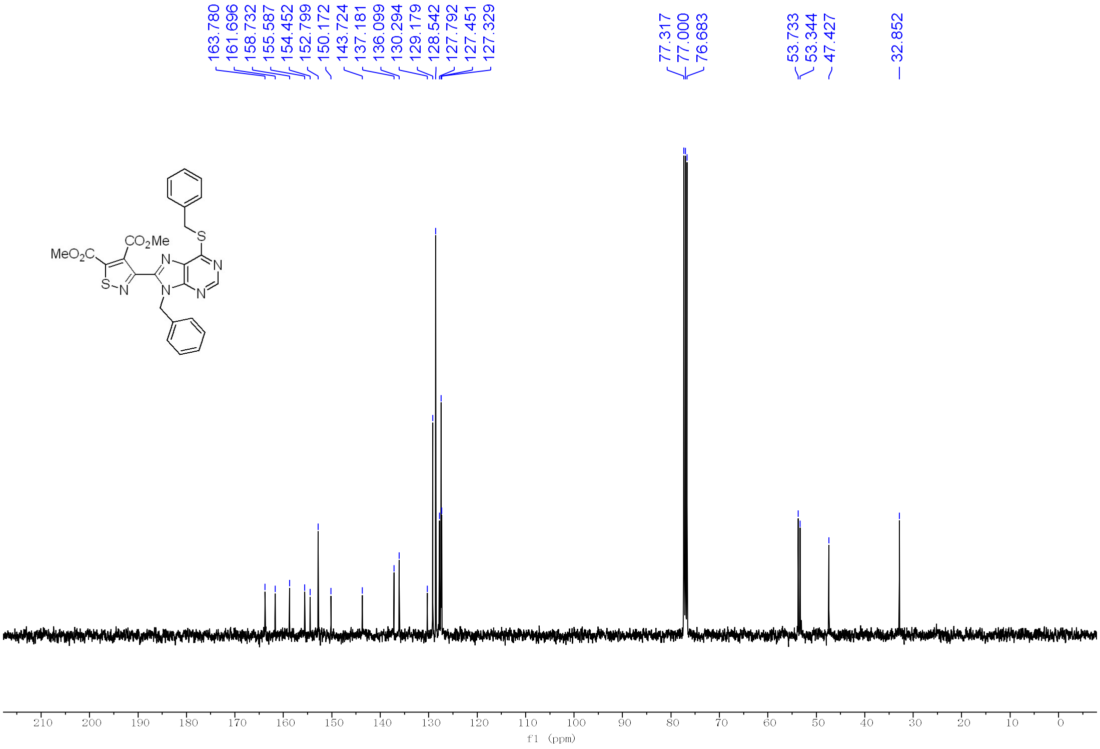

Supplement: Supplementary file 1 [file molecules-28-00914-s001.zip › final SI clean version.pdf]
